# *Bifidobacteria infantis* and human milk oligosaccharides have independent and synergistic effects on immune response and amino acid metabolism in germ-free mouse models

**DOI:** 10.1128/msystems.00392-26

**Published:** 2026-06-15

**Authors:** Bharath K. Mulakala, Michael L. Salinas, Laurie A. Davidson, Selim Romero, Quentin D. Read, Renee Fox, Tanya LeRoith, James J. Cai, Robert S. Chapkin, Sharon M. Donovan, Laxmi Yeruva

**Affiliations:** 1 Microbiome and Metabolism Research Unit, Arkansas Children’s Nutrition Center, USDA-ARS 57731, Little Rock, Arkansas, USA; 2 Texas A&M AgriLife Institute for Advancing Health Through Agriculture 686443, College Station, Texas, USA; 3 Department of Nutrition, Texas A&M University 199052, College Station, Texas, USA; 4 CPRIT Single Cell Data Science Core, Texas A&M University 14736, College Station, Texas, USA; 5 Department of Veterinary Integrative Biosciences, Texas A&M University 199062, College Station, Texas, USA; 6 USDA-ARS, Southeast Area 57579 , Raleigh, North Carolina, USA; 7 Arkansas Children's Nutrition Center 57731, Little Rock, Arkansas, USA; 8 Department of Biomedical Sciences & Pathobiology, Virginia Tech 1757, Blacksburg, Virginia, USA; 9 Department of Electrical and Computer Engineering, Texas A&M University 189673, College Station, Texas, USA; 10 Department of Food Science and Human Nutrition, University of Illinois Urbana-Champaign 242191, Urbana, Illinois, USA; Boston College, Chestnut Hill, Massachusetts, USA

**Keywords:** *Bifidobacterium longum *subsp. *infantis *ATCC 15697, human milk oligosaccharides, germ-free mice, immune modulation, amino acid metabolism

## Abstract

Early-life microbial colonization is essential for gut and immune development. Human milk oligosaccharides (HMOs) support the growth of *Bifidobacterium infantis* (BI). Here, we studied the individual and combined effects of BI and HMOs on the immune and colon transcriptomes and on serum and cecal metabolome. Germ-free mice were randomly assigned to four groups (10–14/group: HMO, BI, BI + HMO, and control [no HMO or BI]). HMO and BI + HMO groups received 5 mg/day each of 2′-fucosyllactose, lacto-N-tetraose, and 3′-sialyllactose for 14 days. BI and BI + HMO received BI ATCC 15,697 (1 × 10^9^ CFU/day) on days 1, 4, and 9. Mono-colonization with BI increased monocytes, macrophages, B cells, CD4^+^ T cells, and Treg cells in mesenteric lymph nodes (MLN) relative to controls. In the spleen, BI alone increased B cells, dendritic cells, Th17 cells, and ILC3 cells, and enriched serum amino acid metabolism pathways. Additionally, BI influenced colonocyte gene expression and modulated serum metabolites that regulate circadian rhythms. BI + HMO increased MLN Th17 cells and spleen monocytes compared with HMO alone. Collectively, the results of this study highlight the complex interplay among host-microbe-diet interactions and emphasize the importance of considering these interactions when designing strategies to modulate infant health during early life.

**IMPORTANCE:**

Early life immune and gut microbiome development are shaped by human milk (HM). One of the most important drivers of these processes is the human milk oligosaccharides (HMOs). *Bifidobacterium infantis* (BI) possesses a unique enzymatic system that enables efficient HMO uptake and intracellular metabolism, providing a competitive advantage over other microbial species in the breastfed infant gut. To date, the potential direct and synergistic effects of BI and HMO have not been fully explored. The knowledge generated herein identified the independent and synergistic effects of HMOs and BI on gut immune response, serum and cecal metabolites, and colonic gene expression.

## INTRODUCTION

Clinical and epidemiological studies support reduced upper respiratory tract, ear, and gastrointestinal infections in breastfed compared with formula-fed infants (1–6). Human milk contains bioactive components that serve non-nutritive roles including human milk oligosaccharides (HMOs), the third most abundant solid component of HM. There are >150 different HMO structures, with 2′-fucosyllactose (2′-FL), 3′-fucosyllactose (3′-FL), 6′-sialyllactose (6′-SL), lacto-N-tetraose (LNT), and lacto-N-neotetraose (LNnT) among the most abundant. Maternal genetics influence the types of HMOs produced, with non-secretor mothers producing no 2′-FL or longer-chain 2-fucosylated HMOs. Recent *in vivo* findings have documented direct effects of HMOs on the immune system (7).

Intestinal and immune system development are affected by both diet and gut microbes. HM-fed infants have higher abundances of fecal *Bifidobacteria* and *Bacteroides* than formula-fed infants (8–16). *Bifidobacteria* species produce indole lactic acid (ILA), phenyl lactic acid, and 4-hydroxyphenyllactic acid in germ-free mice (17), with ILA exhibiting anti-inflammatory effects in intestinal cells (18). In addition, supplementation with BI in the first 1–12 weeks of life in term infants increased fecal acetic acid and IgA, suggesting a role for BI in the neonatal immune response (19). Moreover, we demonstrated in germ-free mice that an infant microbial community containing BI increased cecal *Clostridium senu stricto*, *Ruminococcus gnavus*, *Cellulosilyticum* sp., and *Erysipelatoclostridium* sp., and modulated butyrate, serotonin, and indole metabolism compared with mice colonized with an infant microbial community lacking BI (20). These studies support a role for BI in metabolite production and immune response.

**Fig 1:**
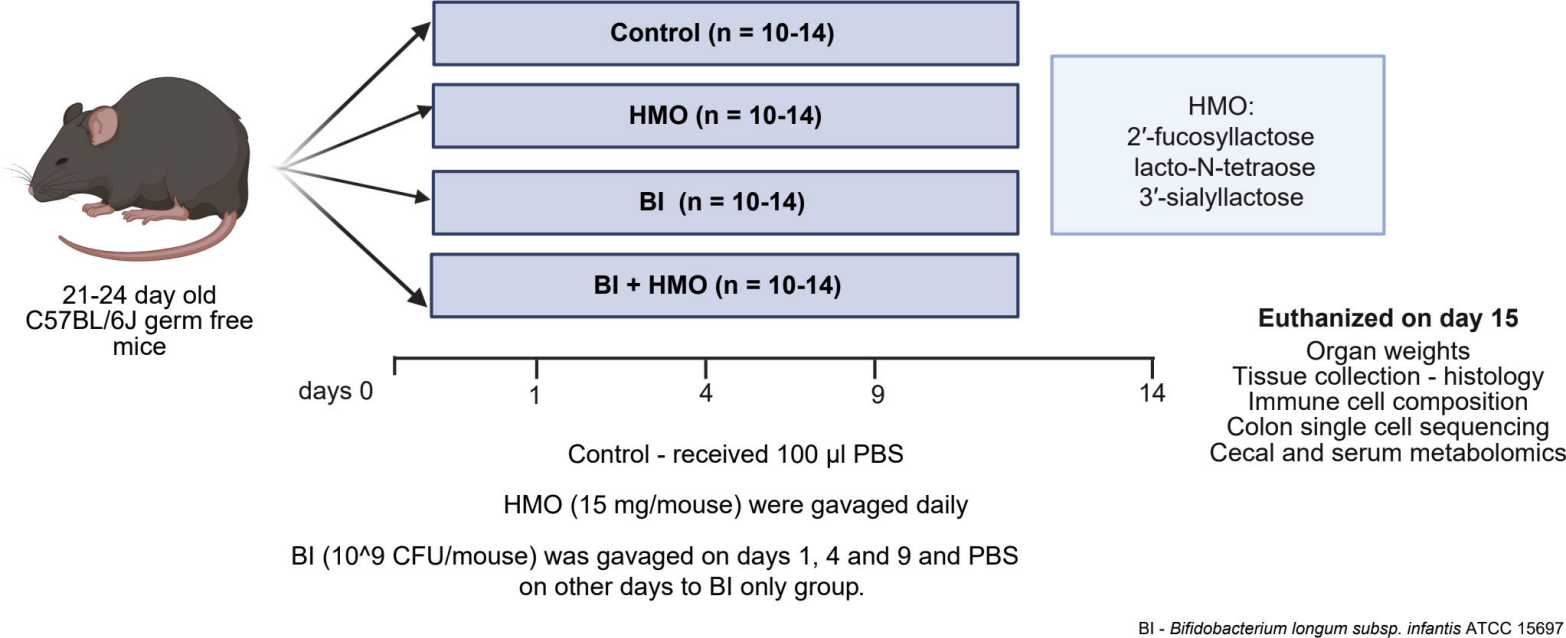
Schematic representation of the study timeline and group assignments. Treatment groups: HMO (human milk oligosaccharides) group, orally gavaged with pooled 2′-fucosyllactose, lacto-N-tetraose, and 3′-sialyllactose at 15 mg/day (5 mg/HMO) for 14 days; BI group, orally gavaged with *Bifidobacterium longum* subsp. *infantis* ATCC 15697 (1 × 10^9^ CFU/day) on days 1, 4, and 9 of the 14-day experimental period; BI + HMO group, orally gavaged with HMO for all 14 days and BI on days 1, 4, and 9 of the 14-day experimental period; control group, orally gavaged with PBS for 14 days.

HMOs are multi-functional components that serve as substrates for different *Bifidobacteria* species, preventing the adhesion of pathogens to epithelial cell surfaces (21), and promoting immune function (22). *Bifidobacteria* species vary in their genetic capacity to utilize HMOs (23–27). Among *Bifidobacteria* species, BI harbors several unique gene clusters for transporting and utilizing HMO (28–32). Although BI and HMO likely have an interactive effect on the host, there is limited information on the effects of BI in the absence or presence of HMOs on the host immune system and metabolic processes. Germ-free mice colonized with microbiota can be used to investigate direct vs indirect (e.g., mediated through the microbiome) actions of the most abundant and different classes of HMOs (i.e., 2′-FL, 3′-SL, and LNT) that are added to infant formulas to modulate infant gut microbiota, specifically *Bifidobacteria*, and host immune function (33–37). We hypothesized that BI alone or in the presence of HMOs would influence host immune cell composition, biological, and metabolic processes. Thus, we systematically dissected the role of these HMOs, alone or in combination with BI*,* in a gnotobiotic mouse model to help improve infant formula composition (Fig. 1).

## RESULTS

### *B. infantis* supplementation influences spleen, liver, cecum weights, and ileum crypt depth

The germ-free status of control and HMO-supplemented mice was confirmed by culture methods (Table S3; Fig. S1), and no growth was observed on agar plates (blood and antibiotic-free Sabouraud dextrose) or in broth (thioglycolate and tryptic soy) with samples from the HMO or control groups. Growth was observed on both agar plates and in broth with samples from the BI + HMO group, whereas growth was only observed in thioglycolate broth for the BI group. Colonization of BI in the BI group was confirmed by PCR (Table S4). Furthermore, molecular validation of the germ-free status of mice was performed using 16S, 18S, and ITS2 primers (Table S5), and the products were analyzed via electrophoresis on 2% agarose gels using SYBR Safe (Invitrogen E-Gel Power Snap plus Electrophoresis System, Thermo Fisher Scientific) (Fig. S2).

**Fig 2:**
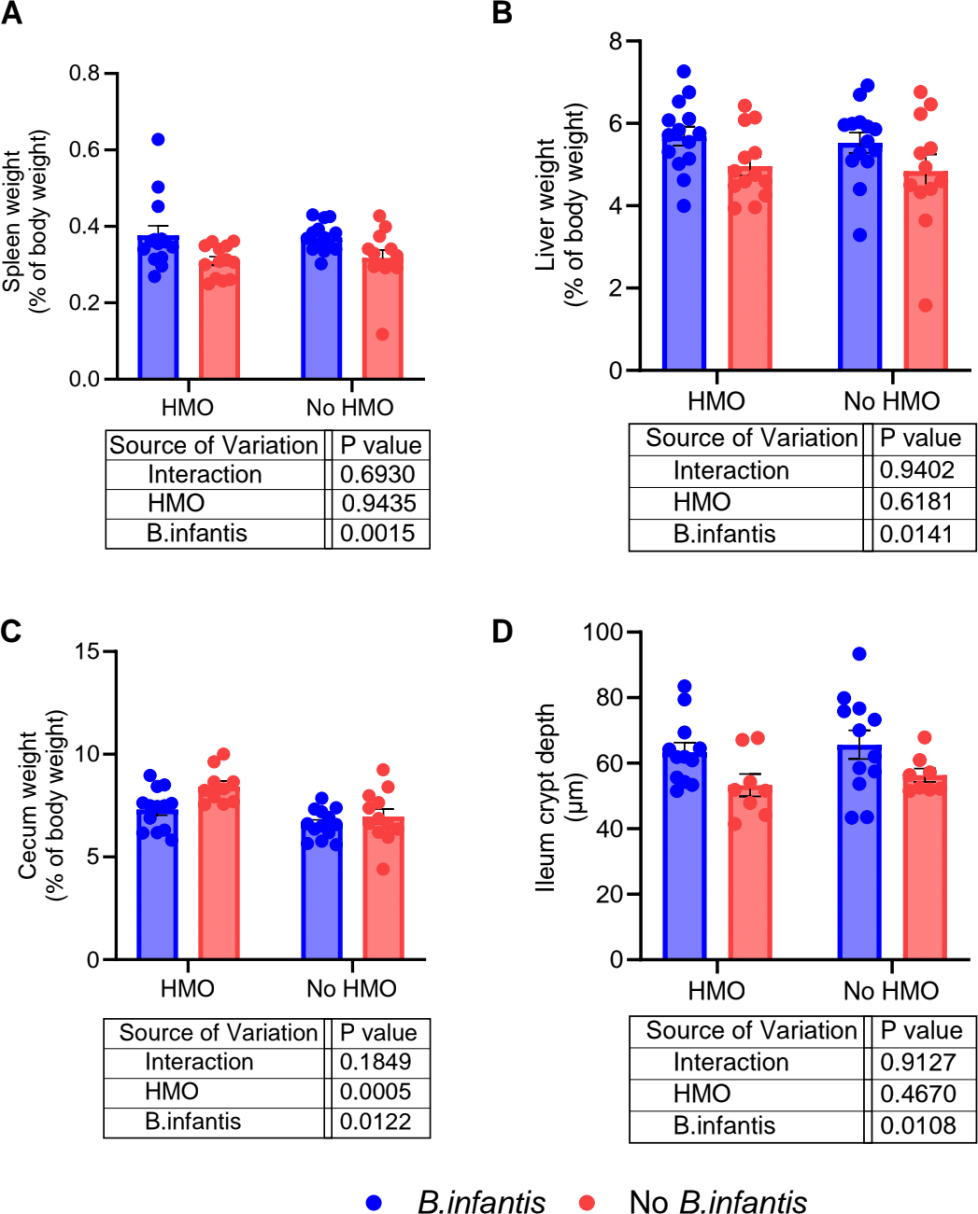
The effect of BI and HMO oral gavage on (**A**) spleen, (**B**) liver and (**C**) cecum weights (including contents) relative to body weight, and (**D**) ileum crypt depth in germ-free mice (*n* = 10–14 mice/group). The four treatment groups were as follows: HMO (human milk oligosaccharides) group, orally gavaged with pooled 2′-fucosyllactose, lacto-N-tetraose and 3′-sialyllactose at 15 mg/day (5 mg/HMO) for 14 days; BI group, orally gavaged with *Bifidobacterium longum* subsp. *infantis* ATCC 15697 (1 × 10^9^ CFU/d) on days 1, 4, and 9 of the 14-day experimental period; BI + HMO group, orally gavaged with HMO for all 14 days and BI on days 1, 4, and 9 of the 14-day experimental period; control group, orally gavaged with PBS for 14 days. Outliers were removed using the ROUT method (*Q* = 0.1%), and *P*-values were calculated using two-way ANOVA with Tukey’s multiple comparison tests in GraphPad Prism Version 10.0. Means ± SEM are plotted.

We evaluated the effects of BI, HMO, or their interaction on organ weights and gut morphology. Spleen and liver weights (% of body weight) were higher in both BI and BI + HMO groups (Fig. 2A and B) compared with those that did not receive BI (HMO and control groups). However, the cecum weight was influenced by BI and HMO supplementation status (Fig. 2C). Specifically, the cecum weight was lower in BI mice but higher in both HMO groups (HMO and BI + HMO) compared with mice that did not receive HMO (BI and control). Also, no significant impact of BI or HMO supplementation, or their interaction, was observed on gut morphology (Fig. S3) except for ileal crypt depth; BI groups exhibited increased crypt depth (Fig. 2D) compared with those that did not receive BI.

### *B. infantis* shapes spleen and MLN immune cell composition

**Fig 3:**
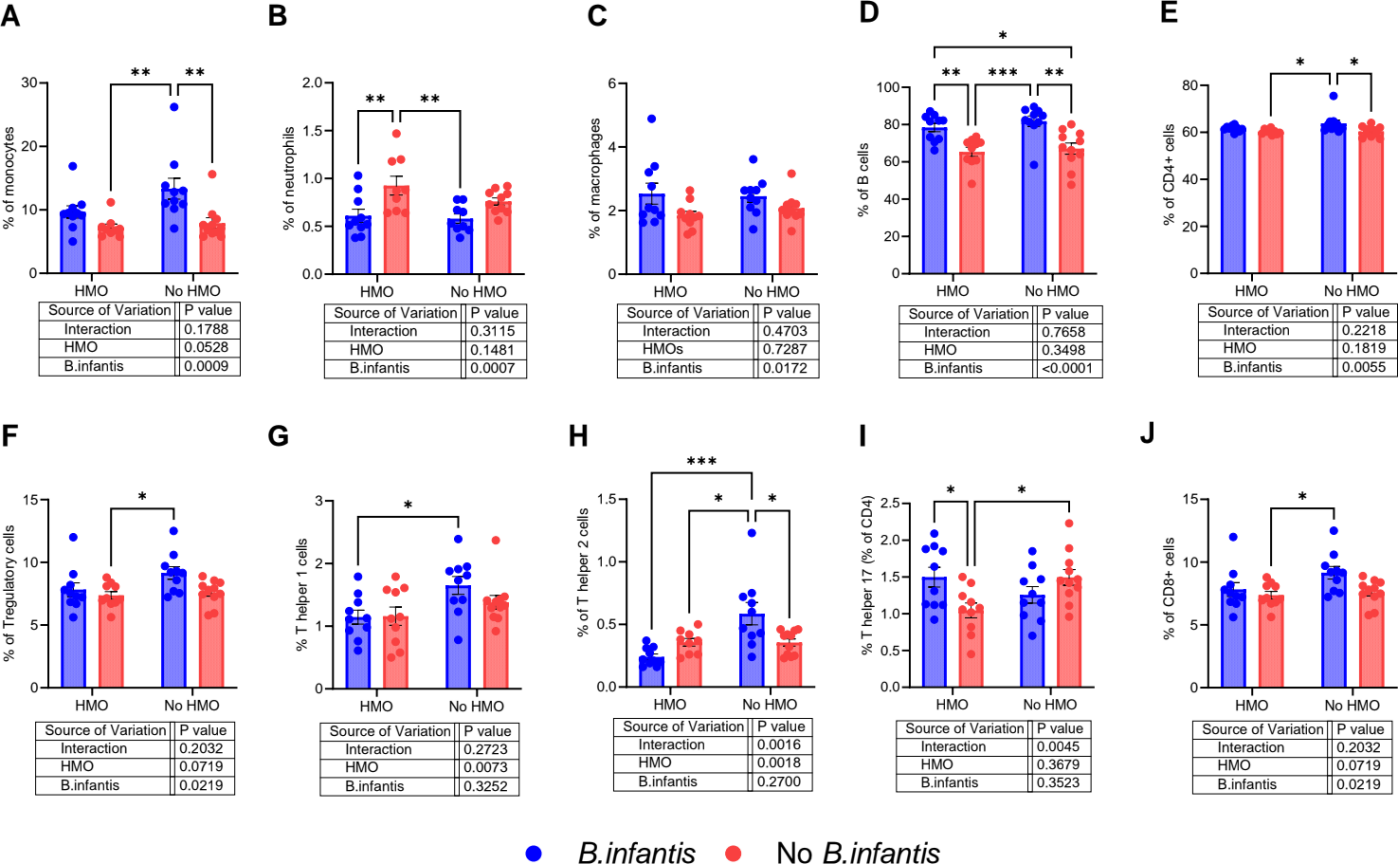
Effect of BI and HMO supplementation on mesenteric lymph node immune cell compositions in germ-free mice collected on day 35 of life (*n* = 10–14 mice/group) as assessed by flow cytometry. Percentage of (**A**) monocytes (CD90^–^/CD19^–^/CD11c^–^/LY6C^+^), (**B**) neutrophils (CD90^–^/CD19^–^/CD11c^–^/LY6G^+^), (**C**) macrophages (CD90^–^/CD19^–^/CD11c^–^/CD64^+^), (**D**) B cells (CD90^–^/CD19^+^), (**E**) CD4^+^ T cells (CD90^+^/T cell alpha-beta receptor^+^/CD4^+^), (**F**) regulatory T cells (CD90^+^/T cell alpha-beta receptor^+^/CD4^+^/FOXP3^+^), (**G**) T helper 1 cells (CD90^+^/T cell alpha-beta receptor^+^/CD4^+^/T-BET^+^), (**H**) T helper 2 cells (CD90^+^/T cell alpha-beta receptor^+^/CD4^+^/GATA3^+^), (**I**) T helper 17 cells (CD90^+^/T cell alpha-beta receptor^+^/CD4^+^/ROGAMMA^+^), and (**J**) CD8^+^ T cells (CD90^+^/T cell alpha-beta receptor^+^/CD8^+^). Treatment groups: HMO (human milk oligosaccharides) group, orally gavaged with pooled 2′-fucosyllactose, lacto-N-tetraose and 3′-sialyllactose at 15 mg/day (5 mg/HMO) for 14 days; BI group, orally gavaged with *Bifidobacterium longum* subsp. *infantis* ATCC 15697 (1 × 10^9^ CFU/d) on days 1, 4, and 9 of the 14-day experimental period; BI + HMO group, orally gavaged with HMO for all 14 days and BI on days 1, 4, and 9 of the 14-day experimental period; control group, orally gavaged with PBS for 14 days. Outliers were removed using the ROUT method (*Q* = 0.1%), and *P*-values were calculated using two-way ANOVA with Tukey’s multiple comparison tests in GraphPad Prism Version 10.0. Percent of population ± SEM are plotted (*P ≤ 0.05, **P ≤ 0.01, ***P ≤ 0.001).

**Fig 4:**
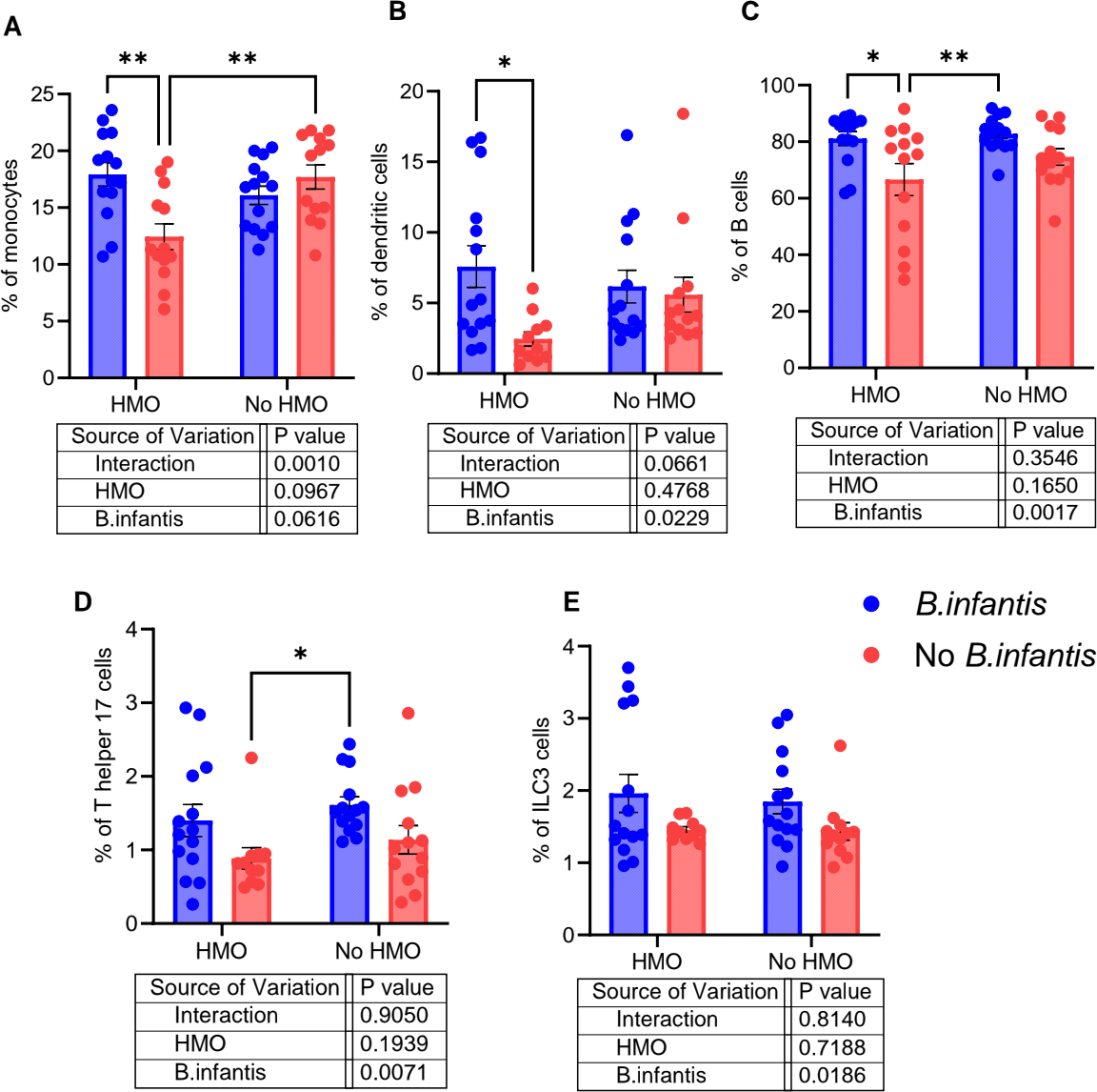
Effect of BI and HMO supplementation on spleen immune cell compositions of germ-free mice (*n* = 10–14 mice/group) as assessed by flow cytometry. Percentage of (**A**) monocytes (CD90^–^/CD19^–^/CD11c^–^/LY6C^+^), (**B**) dendritic cells (CD90^–^/CD19^–^/CD11c^+^), (**C**) B cells (CD90^–^/CD19^+^), (**D**) T helper 17 cells (CD90^+^/T cell alpha-beta receptor^+^/CD4^+^/ROGAMMA^+^), and (**E**) innate lymphoid cell type 3 (CD90^+^/T cell alpha-beta receptor^–^/ROGAMMA^+^). Treatment groups: HMO (human milk oligosaccharides) group, orally gavaged with pooled 2′-fucosyllactose, lacto-N-tetraose and 3′-sialyllactose at 15 mg/day (5 mg/HMO) for 14 days; BI group, orally gavaged with *Bifidobacterium longum* subsp. *infantis* ATCC 15697 (1 × 10^9^ CFU/day) on days 1, 4, and 9 of the 14-day experimental period; BI + HMO group, orally gavaged with HMO for all 14 days and BI on days 1, 4, and 9 of the 14-day experimental period; control group, orally gavaged with PBS for 14 days. Outliers were removed using the ROUT method (*Q* = 0.1%), and *P*-values were calculated using two-way ANOVA with Tukey’s multiple comparison tests in GraphPad Prism Version 10.0. Percent of population ± SEM are plotted (*P ≤ 0.05, **P ≤ 0.01).

To test the influence of mono-colonization of BI on immune development in the presence and absence of HMOs, the immune cell composition of the spleen and MLN was evaluated. BI supplementation primarily influenced the immune cell composition of spleen and MLN (Fig. 3 and 4). In MLN, the percentage of monocytes (% of live single cells), macrophages (% of live single cells), B cells (% of live single cells), CD4^+^ T cells (% of T cell alpha-beta receptor^+^ cells), CD8^+^ T (% of T cell alpha-beta receptor^+^ cells) cells, and regulatory T cells (% of CD4^+^ T cells) were higher in mice receiving BI compared with those that did not receive BI, while the percentage of neutrophils was lower (Fig. 3). HMO supplementation only affected MLN Th1 cells (% of CD4^+^ T cells), which were lower in mice receiving HMO (HMO and BI + HMO groups) compared with those that did not receive HMO (BI and control groups). MLN Th17 and Th2 cells were influenced by BI × HMO interactions. The percentage of Th2 cells (% of CD4^+^ T cells) was higher in the BI group than in all other groups, while the percentage of MLN Th17 cells (% of CD4^+^ T cells) was lower in the HMO group than in the BI + HMO and control groups. The BI group exhibited a higher population of regulatory T cells and Th1 cells than the HMO and BI + HMO groups, respectively. Additionally, MLN CD4^+^ T cells and monocytes were higher in the BI group than in the HMO and control groups. Finally, the percentage of B cells was higher in the BI groups than in the HMO or control groups.

In the spleen, the percentage of dendritic cells (% of live single cells), B cells, Th17 cells, and ILC3 (% of T cell alpha-beta receptor^-^ cells) was higher in BI received mice (Fig. 4) than in those that did not receive BI. Also, the percentage of splenic monocytes was lower in the HMO group than in the BI + HMO and control groups. While fewer significant BI × HMO interactions were observed, the pairwise comparisons revealed significant differences between treatment groups for specific MLN and spleen immune cell populations. The HMO group had fewer splenic B cells and a higher neutrophil count in the MLN compared with the BI and BI + HMO groups. Furthermore, HMO treatment reduced the number of splenic dendritic cells and Th17 cells than the BI + HMO and BI groups, respectively.

### Colon single-cell transcriptomics

**Fig 5:**
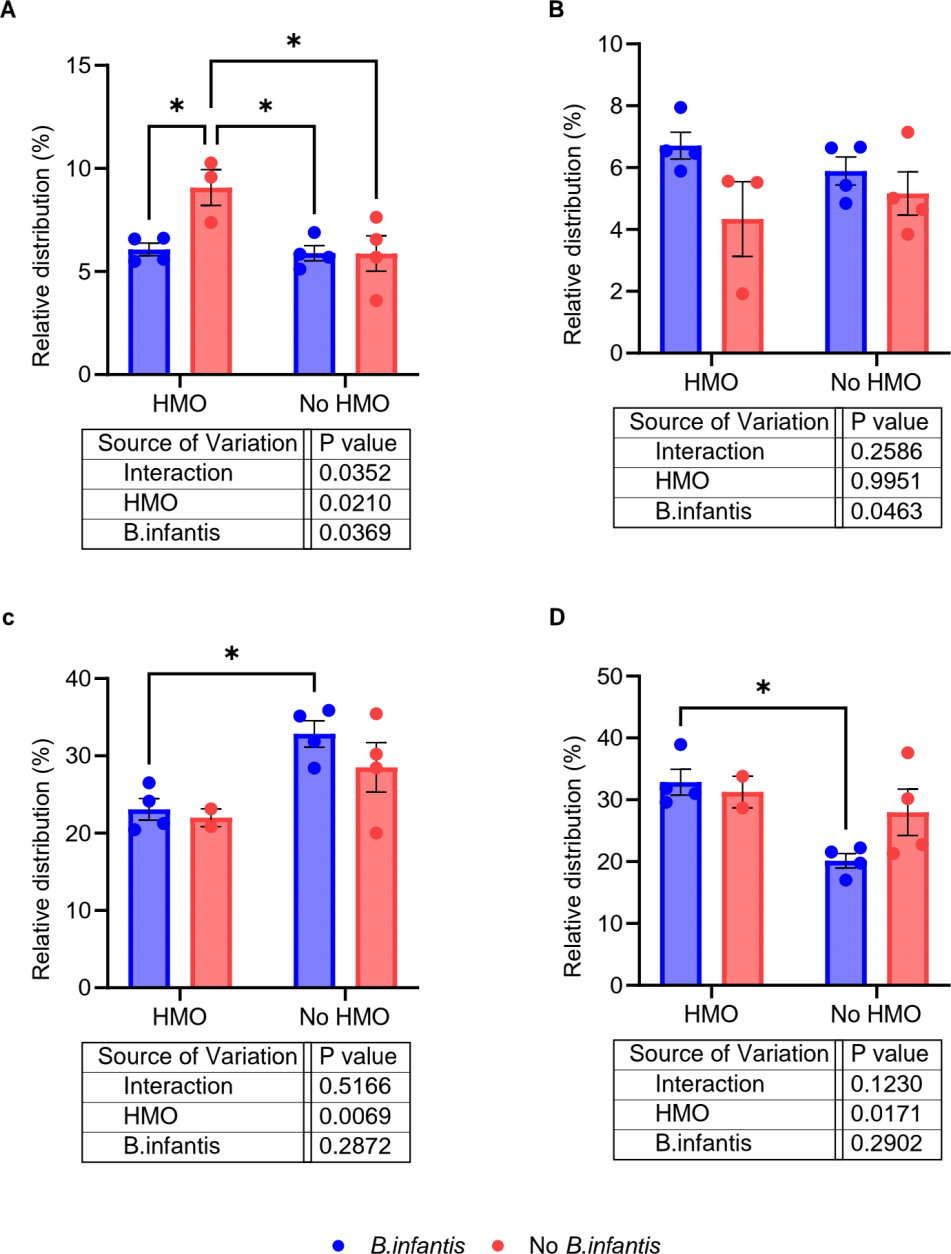
Influence of BI and HMO supplementation on the relative abundance of colon cell populations (*n* = 3–4 mice/group) as assessed by single-cell RNA sequencing. Relative abundance of (**A**) TA cells and (**B**) deep crypt secretory cells out of total colonocytes across treatment groups. Relative abundance of (**C**) ILC1 and (**D**) ILC2 out of the total ILC population across treatment groups. Treatment groups: HMO (human milk oligosaccharides) group, orally gavaged with pooled 2′-fucosyllactose, lacto-N-tetraose, and 3′-sialyllactose at 15 mg/day (5 mg/HMO) for 14 days; BI group, orally gavaged with *Bifidobacterium longum* subsp. *infantis* ATCC 15697 (1 × 10^9^ CFU/day) on days 1, 4, and 9 of the 14-day experimental period; BI + HMO group, orally gavaged with HMO for all 14 days and BI on days 1, 4, and 9 of the 14-day experimental period; control group, orally gavaged with PBS for 14 days. *P*-values were calculated using two-way ANOVA with Tukey’s multiple comparison tests in GraphPad Prism Version 10.0. Mean ± SEM are plotted (*P ≤ 0.05).

The impact of BI mono-colonization, in the presence and absence of HMOs, on colon tissue single-cell transcriptomics was assessed. There was an average of 5,169 epithelial cells (colonocytes) and 2,957 non-epithelial cells per sample across treatment groups (Table S6). Cluster analysis identified 10 major populations of colonocytes (Fig. S4A and B) and 13 major populations of non-epithelial cells (Fig. S5A and B) using specific cell markers. Within the colonocyte population, the relative abundance of major cell types did not differ between treatment groups (Fig. S4C), except for transit-amplifying (TA) cells and deep crypt secretory cells (Fig. 5A and B). The relative abundance of TA cells was higher in the HMO group than in the other groups, whereas the relative abundance of deep secretory cells was higher in mice administered BI than in those that did not receive BI. Similarly, among the non-epithelial cells, the relative abundance of the major cell types was not different between treatment groups (Fig. S5C). However, within the T cell cluster were populations of T cell subtypes, ILC subtypes, and mast cells (Fig. S6). The relative abundance of ILC1 was higher in the BI group compared with the BI + HMO group (Fig. 5C). In contrast, the relative abundance of ILC2 was lower in the BI group compared with the BI + HMO group (Fig. 5D).

Next, the pathway enrichment scoring analysis revealed that colonocytes from BI mice were enriched for amino acid-related biological processes, such as amino acid transmembrane transport, amino acid import across the plasma membrane, and neutral amino acid transport, compared with colonocytes from mice that did not receive BI. Furthermore, specific amino acid-related biological processes scored higher in colonocytes from BI mice than in those from mice not receiving BI, including alanine transport, glutamine transport, and L-glutamine import across the plasma membrane, serine transport and the serine family amino acid metabolic process, and proline transport. Additionally, the entrainment of the circadian clock biological process UCell score was higher in colonocytes from BI mice than in those from mice not receiving BI. Within the non-epithelial cells, the enrichment score of monoacylglycerol catabolic processes was lower in colonic adipocytes from BI mice compared with those that did not receive BI. Interestingly, the enrichment score of plasma lipoprotein particle assembly was higher in colonic adipocytes from BI mice compared with those that did not receive BI. Furthermore, the enrichment score of fatty acid beta oxidation was lower in colonic adipocytes from HMO mice compared with those that did not receive HMO. The list of significantly enriched biological processes across treatment groups in colonocytes and non-epithelial cells is provided in Table 1. A two-way ANOVA differential gene expression analysis across major cell types evaluated the influence of the supplementary status of BI and HMO at the gene level. Differential gene expression analysis revealed that BI-status-modulated genes involved in circadian rhythms (*Per1, Per2*, *Per3, Ciart, Nfil3, Dbp,* and *Nr1d2*) across 13 different cell subtypes in colon tissue (Table 2). BI mice exhibited higher mRNA expression of *Per1, Per2*, *Per3, Ciart, Dbp*, and *Nr1d2* than mice that did not receive BI.

**TABLE 1:** Enrichment scores of various biological processes in colonocytes and non-epithelial cells (*n* = 3–4 mice/group)^*a*^

Cell type	MSigDB pathway	BI		No BI		Adjusted *P* value		
		HMOs	No HMOs	HMOs	No HMOs	HMOs	BI	BI × HMOs
All colonocytes	GOBP_ALANIE_TRANSPORT	0.074 ^a^	0.081 ^a^	0.064 ^a^	0.071 ^a^	0.146	0.030	0.944
	GOBP_AMINO_ACID_IMPORT_ACROSS_PLASMA_MEMBRANE	0.067 ^a^	0.069 ^a^	0.063 ^a^	0.065 ^a^	0.175	0.030	0.960
	GOBP_AMINO_ACID_NEUROTRANSMITTER_REUPTAKE	0.041 ^ab^	0.053 ^a^	0.036 ^b^	0.036 ^b^	0.116	0.012	0.113
	GOBP_AMINO_ACID_TRANSMEMBRANE_TRANSPORT	0.065 ^ab^	0.066 ^a^	0.063 ^b^	0.064 ^ab^	0.115	0.003	0.639
	GOBP_ARGININE_CATABOLIC_PROCESS	0.031 ^a^	0.032 ^a^	0.039 ^a^	0.035 ^a^	0.393	0.013	0.212
	GOBP_ARGININE_METABOLIC_PROCESS	0.029 ^ab^	0.027 ^b^	0.033 ^a^	0.031 ^ab^	0.101	0.010	0.913
	GOBP_ENTRAINMENT_OF_CIRCADIAN_CLOCK	0.054 ^a^	0.057 ^a^	0.047 ^b^	0.052 ^ab^	0.023	0.002	0.567
	GOBP_CIRCADIAN_REGULATION_OF_GENE_EXPRESSION	0.089 ^ab^	0.092 ^a^	0.085 ^b^	0.091 ^a^	0.008	0.116	0.236
	GOBP_GLUTAMINE_TRANSPORT	0.091 ^ab^	0.093 ^a^	0.078 ^b^	0.083 ^ab^	0.295	0.005	0.754
Adipocytes	GOBP_L_GLUTAMINE_IMPORT_ACROSS_PLASMA_MEMBRANE	0.151 ^a^	0.158 ^a^	0.129 ^a^	0.138 ^a^	0.248	0.009	0.855
	GOBP_L_SERINE_TRANSPORT	0.163 ^ab^	0.174 ^a^	0.136 ^b^	0.148 ^ab^	0.204	0.010	0.984
	GOBP_NEUTRAL_AMINO_ACID_TRANSPORT	0.062 ^a^	0.064 ^a^	0.057 ^a^	0.060 ^a^	0.182	0.032	0.868
	GOBP_PROLINE_TRANSPORT	0.053 ^a^	0.055 ^a^	0.042 ^a^	0.050 ^a^	0.165	0.028	0.359
	GOBP_RETROGRADE_TRANS_SYNAPTIC_SIGNALING_BY_LIPID	0.014 ^a^	0.015 ^a^	0.010 ^a^	0.011 ^a^	0.554	0.009	0.972
	GOBP_SERINE_FAMILY_AMINO_ACID_METABOLIC_PROCESS	0.049 ^a^	0.053 ^a^	0.047 ^a^	0.048 ^a^	0.233	0.040	0.392
	GOBP_SERINE_TRANSPORT	0.100 ^a^	0.106 ^a^	0.085 ^a^	0.092 ^a^	0.186	0.013	0.890
	GOBP_FATTY_ACID_BETA_OXIDATION_USING_ACYL_COA_DEHYDROGENASE	0.130 ^ab^	0.179 ^a^	0.116 ^b^	0.138 ^ab^	0.018	0.052	0.312
	GOBP_MONOACYLGLYCEROL_CATABOLIC_PROCESS	0.075 ^ab^	0.080 ^a^	0.134 ^b^	0.096 ^ab^	0.238	0.014	0.116
	GOBP_PLASMA_LIPOPROTEIN_PARTICLE_ASSEMBLY	0.134 ^a^	0.142 ^a^	0.101 ^a^	0.101 ^a^	0.820	0.044	0.812

^*a*^ Treatment groups: HMO (human milk oligosaccharides) group, orally gavaged with pooled 2′-fucosyllactose, lacto-N-tetraose, and 3′-sialyllactose at 15 mg/day (5 mg/HMO) for 14 days; BI group, orally gavaged with *Bifidobacterium longum* subsp. *infantis* ATCC 15697 (1 × 10^9 CFU/day) on days 1, 4, and 9 of the 14-day experimental period; BI + HMO group, orally gavaged with HMO for all 14 day and BI on days 1, 4, and 9 of the 14-day experimental period; control group, orally gavaged with PBS for 14 day. *P*-values were calculated using two-way ANOVA with Tukey’s multiple comparison tests in GraphPad Prism Version 10.0. Mean ± SEM are plotted. Mean values in the same row with different superscripts are statistically different (*P* < 0.05).

**TABLE 2:** The average gene expression of circadian rhythm-associated genes in the colonocyte cell population was affected due to BI and HMO supplementary status (*n* = 3–4 mice/group) as assessed by single-cell RNA seq^*a*^

Cell type	Gene name	BI		No BI		Adjusted *P* value		
		HMOs	No HMOs	HMOs	No HMOs	HMOs	BI	BI × HMOs
Absorptive progenitor cells	*Nfil3*	0.017 ^a^	0.013 ^a^	0.051 ^a^	0.035 ^a^	0.525	0.031	1.000
	*Ciart*	0.009 ^a^	0.032 ^b^	0.003 ^a^	0.005 ^a^	0.270	0.028	1.000
	*Dbp*	0.198 ^ab^	0.510 ^b^	0.042 ^a^	0.091 ^ab^	0.525	0.031	1.000
Enteric glial cells	*Dbp*	0.487 ^ab^	0.644 ^b^	0.286 ^a^	0.323 ^a^	0.414	0.006	0.517
	*Per1*	0.771 ^bc^	0.948 ^c^	0.345 ^a^	0.561 ^ab^	0.328	0.006	0.897
	*Per2*	0.243 ^ab^	0.437 ^b^	0.039 ^a^	0.175 ^a^	0.107	0.007	0.818
	*Per3*	0.317 ^b^	0.304 ^b^	0.066 ^a^	0.163 ^ab^	0.800	0.007	0.511
Fibroblasts	*Dbp*	0.447 ^a^	0.804 ^a^	0.100 ^a^	0.181 ^a^	0.775	0.025	0.740
	*Per3*	0.167 ^a^	0.310 ^a^	0.049 ^a^	0.072 ^a^	0.775	0.025	0.740
	*Per2*	0.170 ^a^	0.306 ^a^	0.054 ^a^	0.108 ^a^	0.775	0.037	0.773
L-cells	*Per3*	0.323 ^a^	0.356 ^a^	0.097 ^a^	0.127 ^a^	0.982	0.009	0.979
	*Dbp*	0.368 ^ab^	0.457 ^b^	0.128 ^a^	0.220 ^ab^	0.460	0.004	0.979
Lymphatic endothelial cells	*Nr1d1*	0.209 ^a^	0.255 ^a^	0.084 ^a^	0.134 ^a^	0.687	0.026	0.994
	*Dbp*	0.192 ^ab^	0.248 ^b^	0.047 ^a^	0.079 ^ab^	0.698	0.007	0.914
	*Per3*	0.112 ^a^	0.092 ^a^	0.025 ^a^	0.050 ^a^	0.951	0.024	0.743
Microfold cells	*Dbp*	0.567 ^ab^	0.899 ^b^	0.212 ^a^	0.296 ^a^	0.168	0.003	0.406
	*Per1*	0.874 ^ab^	1.072 ^b^	0.351 ^a^	0.573 ^ab^	0.231	0.008	0.922
	*Nr1d1*	0.640 ^a^	0.858 ^a^	0.287 ^a^	0.470 ^a^	0.197	0.015	0.922
	*Ciart*	0.056 ^a^	0.164 ^a^	0.003 ^a^	0.009 ^a^	0.197	0.017	0.406
Pericytes	*Dbp*	0.364 ^ab^	0.530 ^b^	0.152 ^a^	0.211 ^a^	0.361	0.006	0.718
	*Per2*	0.202 ^ab^	0.362 ^b^	0.034 ^a^	0.156 ^a^	0.078	0.004	0.768
Smooth muscle cells	*Per2*	0.256 ^ab^	0.402 ^b^	0.055 ^a^	0.133 ^ab^	0.832	0.012	0.873
	*Per3*	0.166 ^ab^	0.260 ^b^	0.036 ^a^	0.103 ^ab^	0.832	0.012	0.968
	*Dbp*	0.630 ^ab^	0.817 ^b^	0.141 ^a^	0.294 ^ab^	0.832	0.007	0.991
Tuft cells	*Dbp*	0.180 ^a^	0.247 ^a^	0.050 ^a^	0.120 ^a^	0.343	0.025	0.991
	*Nr1d1*	0.382 ^a^	0.445 ^a^	0.175 ^a^	0.207 ^a^	0.860	0.025	0.991
T cells	*Dbp*	0.127 ^a^	0.147 ^a^	0.079 ^a^	0.080 ^a^	0.982	0.027	0.576
Vascular endothelial cells	*Dbp*	0.218 ^a^	0.433 ^a^	0.073 ^a^	0.130 ^a^	0.669	0.038	0.994
Plasma B cells	*Per1*	0.471 ^a^	0.542 ^a^	0.160 ^a^	0.188 ^a^	0.771	0.008	0.820
Macrophages	*Dbp*	0.061 ^a^	0.113 ^a^	0.015 ^a^	0.044 ^a^	0.248	0.036	0.589

^*a*^ Treatment groups: HMO (human milk oligosaccharides) group, orally gavaged with pooled 2′-fucosyllactose, lacto-N-tetraose, and 3′-sialyllactose at 15 mg/day (5 mg/HMO) for 14 days; BI group, orally gavaged with *Bifidobacterium longum* subsp. *infantis* ATCC 15697 (1 × 10^9^ CFU/day) on days 1, 4, and 9 of the 14-day experimental period; BI + HMO group, orally gavaged with HMO for all 14 days and BI on days 1, 4, and 9 of the 14-day experimental period; control group, orally gavaged with PBS for 14 days. Adjusted *P*-values were calculated using two-way ANOVA with Tukey’s multiple comparison tests in R studio version 4.4.1. Mean values in the same row with different superscripts are statistically different (*P* < 0.05).

### *B. infantis* influences serum and cecal metabolic profiles

To investigate the influence of BI on host metabolomics, we analyzed serum and cecal metabolic profiles across treatment groups. In serum, the abundance of 318 metabolites was significantly altered by BI, with 219 and 99 metabolites higher and lower, respectively, in BI mice than in those that did not receive BI (Table S7). Next, pathway analysis revealed that highly abundant metabolites in BI mice were significantly enriched in (i) butanoate metabolism, (ii) alanine, aspartate, and glutamate metabolism, (iii) arginine biosynthesis, (iv) arginine and proline metabolism, (v) one carbon pool by folate, (vi) sphingolipid metabolism, (vii) glycine, serine, and threonine metabolism, (viii) valine, leucine, and isoleucine biosynthesis, and (ix) histidine metabolism (Table 3). Interestingly the abundance of different types of triglycerides, diacylglycerols, and cholesteryl esters was lower in the serum of BI mice compared with those that did not receive BI. In serum, 104 and 43 metabolites were lower and higher, respectively, in HMO mice than in those that did not receive HMO (Table S8). Pathway analysis of low-abundance metabolites in HMO mice showed enrichment in three amino acid metabolic pathways (valine, leucine, and isoleucine biosynthesis; arginine biosynthesis; and valine, leucine, and isoleucine degradation; FDR < 0.05), while highly abundant metabolites included triglycerides. Interestingly, the abundance of 114 of 147 metabolites altered by HMO supplementation was also affected by BI. However, the abundance of these metabolites was differentially modulated by HMO and BI. For example, the metabolites associated with amino acid metabolic pathways (valine, leucine, and isoleucine biosynthesis; arginine biosynthesis; and valine, leucine, and isoleucine degradation) were higher in BI mice compared with those that did not receive BI. In contrast, they were lower in HMO mice than in mice that did not receive HMO.

**TABLE 3:** Enriched pathways of upregulated serum metabolites in BI mice (BI and BI + HMO groups) compared with those not receiving BI (HMO and control groups) (*n* = 10–14 mice/group) as identified by KEGG pathway in MetaboAnalyst 6.0 software with default parameters

Pathway	Number of hits	FDR adjusted *P*-value
Butanoate metabolism	15	<0.001
Alanine, aspartate, and glutamate metabolism	28	<0.001
Arginine biosynthesis	14	<0.001
Arginine and proline metabolism	36	0.002
One carbon pool by folate	26	0.011
Sphingolipid metabolism	32	0.030
Glycine, serine, and threonine metabolism	34	0.036
Valine, leucine, and isoleucine biosynthesis	8	0.039
Histidine metabolism	16	0.039

Interestingly, 45 serum metabolites were altered by BI × HMO interactions. Specifically, 42 metabolites were differentially regulated between the BI + HMO and BI groups, with all 42 metabolites more abundant in the BI group (Table 4). Pathway enrichment analysis revealed enrichment of glyoxylate and dicarboxylate metabolism and the citric acid cycle in the BI group (FDR < 0.05).

**TABLE 4:** Serum metabolites that are differentially regulated due to BI × HMO interactions (*n* = 10–14 mice/group)^*a*^

Metabolite	Class	HMD ID	BI		No BI		Adjusted *P*-value, BI × HMO
			HMO	No HMO	HMO	No HMO	
2-Oxoisocaproic acid	Organic acids	HMDB0000695	0.012 ^b^	1.369 ^c^	−0.738 ^a^	−0.643 ^a^	0.028
3-Deoxyglucosone	Organic acids	HMDB0005876	−0.291 ^a^	1.205 ^b^	−0.436 ^a^	−0.479 ^a^	0.033
Acetic acid	Short chain fatty acid	HMDB0000042	−0.232 ^a^	1.125 ^b^	−0.267 ^a^	−0.626 ^a^	0.029
C0	Acylcarnitines	HMDB0000062	−0.006 ^b^	1.201 ^c^	−0.400 ^ab^	−0.795 ^a^	0.028
C2	Acylcarnitines	HMDB0000201	0.048 ^b^	1.265 ^c^	−0.624 ^a^	−0.689 ^a^	0.038
C3OH	Acylcarnitines	HMDB0013125	−0.003 ^b^	1.397 ^c^	−0.651 ^a^	−0.744 ^a^	0.028
*cis*-Aconitic acid	Amino acid related	HMDB0000072	−0.386 ^ab^	0.516 ^b^	0.488 ^b^	−0.618 ^a^	0.029
Citric acid	Organic acids	HMDB0000094	−0.378 ^a^	0.911 ^b^	0.057 ^ab^	−0.589 ^a^	0.028
Glyceric acid	Organic acids	HMDB0000139	−0.264 ^a^	1.318 ^b^	−0.680 ^a^	−0.374 ^a^	0.040
Isocitric acid	Organic acids	HMDB0000193	−0.552 ^a^	−0.030 ^a^	1.083 ^b^	−0.501 ^a^	0.028
LysoPC a C24:0	Glycerophospholipids	HMDB0010405	0.167 ^b^	1.358 ^c^	−0.842 ^a^	−0.683 ^a^	0.032
LysoPC a C26:0	Glycerophospholipids	HMDB0029205	0.228 ^b^	1.285 ^c^	−0.741 ^a^	−0.772 ^a^	0.038
LysoPC a C26:1	Glycerophospholipids	HMDB0029220	0.045 ^b^	1.320 ^c^	−0.654 ^a^	−0.711 ^a^	0.028
LysoPC a C28:1	Glycerophospholipids	HMDB0029221	0.004 ^b^	1.343 ^c^	−0.672 ^a^	−0.675 ^a^	0.028
N2-acetyl-ornithine	Amino acid derivatives	HMDB0003357	−0.301 ^ab^	1.409 ^c^	−0.849 ^a^	−0.259 ^b^	0.033
N-acetyl-histidine	Amino acid derivatives	HMDB0032055	−0.009 ^a^	1.168 ^b^	−0.475 ^a^	−0.684 ^a^	0.045
PC aa C24:0	Glycerophospholipids	HMDB0341514	0.147 ^b^	1.234 ^c^	−0.583 ^a^	−0.798 ^a^	0.033
PC aa C28:1	Glycerophospholipids	HMDB0007867	0.176 ^b^	1.333 ^c^	−0.676 ^a^	−0.833 ^a^	0.028
PC aa C36:2	Glycerophospholipids	HMDB0000593	0.230 ^b^	1.231 ^c^	−0.657 ^a^	−0.804 ^a^	0.045
PC aa C40:2	Glycerophospholipids	HMDB0008688	0.001 ^a^	1.141 ^b^	−0.420 ^a^	−0.722 ^a^	0.040
PC aa C42:0	Glycerophospholipids	HMDB0008058	0.105 ^b^	1.278 ^c^	−0.617 ^a^	−0.766 ^a^	0.029
PC aa C42:2	Glycerophospholipids	HMDB0008092	0.030 ^b^	1.307 ^c^	−0.654 ^a^	−0.683 ^a^	0.030
PC ae C30:2	Glycerophospholipids	HMDB0013410	0.065 ^b^	1.255 ^c^	−0.558 ^ab^	−0.762 ^a^	0.029
PC ae C32:2	Glycerophospholipids	HMDB0013411	0.239 ^b^	1.354 ^c^	−0.830 ^a^	−0.763 ^a^	0.028
PC ae C36:0	Glycerophospholipids	HMDB0013406	0.149 ^b^	1.249 ^c^	−0.607 ^a^	−0.792 ^a^	0.032
PC ae C36:1	Glycerophospholipids	HMDB0013414	0.085 ^b^	1.173 ^c^	−0.451 ^ab^	−0.807 ^a^	0.032
PC ae C36:2	Glycerophospholipids	HMDB0013418	0.046 ^b^	1.176 ^c^	−0.465 ^ab^	−0.758 ^a^	0.036
PC ae C38:1	Glycerophospholipids	HMDB0013416	0.077 ^b^	1.215 ^c^	−0.492 ^ab^	−0.800 ^a^	0.029
PC ae C38:3	Glycerophospholipids	HMDB0013439	0.062 ^b^	1.258 ^c^	−0.544 ^ab^	−0.776 ^a^	0.028
PC ae C40:2	Glycerophospholipids	HMDB0013437	0.159 ^b^	1.274 ^c^	−0.686 ^a^	−0.746 ^a^	0.038
PC ae C40:3	Glycerophospholipids	HMDB0013445	−0.018 ^b^	1.206 ^c^	−0.409 ^ab^	−0.780 ^a^	0.028
PC ae C40:4	Glycerophospholipids	HMDB0013442	0.106 ^b^	1.260 ^c^	−0.659 ^a^	−0.708 ^a^	0.045
PC ae C40:5	Glycerophospholipids	HMDB0013444	0.104 ^b^	1.307 ^c^	−0.642 ^a^	−0.770 ^a^	0.028
PC ae C42:4	Glycerophospholipids	HMDB0013448	0.065 ^b^	1.297 ^c^	−0.570 ^a^	−0.792 ^a^	0.028
PC ae C42:5	Glycerophospholipids	HMDB0013451	0.072 ^b^	1.187 ^c^	−0.422 ^ab^	−0.836 ^a^	0.028
PC ae C44:3	Glycerophospholipids	HMDB0013449	0.157 ^b^	1.302 ^c^	−0.738 ^a^	−0.721 ^a^	0.038
PC ae C44:6	Glycerophospholipids	HMDB0013450	0.087 ^b^	1.309 ^c^	−0.680 ^a^	−0.716 ^a^	0.030
SM C16:1	Sphingomyelins	HMDB0240613	0.281 ^b^	1.299 ^c^	−0.826 ^a^	−0.754 ^a^	0.049
SM C26:0	Sphingomyelins	HMDB0011698	−0.047 ^a^	1.216 ^b^	−0.469 ^a^	−0.701 ^a^	0.030
SM(OH) C22:2	Sphingomyelins	HMDB0013467	0.166 ^b^	1.314 ^c^	−0.752 ^a^	−0.727 ^a^	0.034
Tartaric acid	Organic acids	HMDB0000956	−0.345 ^a^	1.178 ^b^	−0.288 ^a^	−0.545 ^a^	0.028
Uric acid	Organic acids	HMDB0000289	−0.067 ^ab^	0.719 ^b^	0.200 ^b^	−0.852 ^a^	0.031
Uridine	Organic acids	HMDB0000296	−0.131 ^a^	1.311 ^b^	−0.669 ^a^	−0.510 ^a^	0.038
Valeric acid	Organic acids	HMDB0000892	0.177 ^b^	1.080 ^c^	−0.404 ^ab^	−0.854 ^a^	0.049
Xanthine	Nucleobases	HMDB0000292	0.164 ^b^	−1.139 ^a^	0.199 ^b^	0.776 ^b^	0.028

^*a*^ Values represent the normalized abundance levels and may contain negative values because of normalization (median and pareto scaling). All data processing and normalization were performed using MetaboAnalyst 6.0 software with default parameters. Treatment groups: HMO (human milk oligosaccharides) group, orally gavaged with pooled 2′-fucosyllactose, lacto-N-tetraose, and 3′-sialyllactose at 15 mg/day (5 mg/HMO) for 14 days; BI group, orally gavaged with *Bifidobacterium longum* subsp. *infantis* ATCC 15697 (1 × 10^9^ CFU/day) on days 1, 4, and 9 of the 14-day experimental period; BI + HMO group, orally gavaged with HMO for all 14 d and BI on days 1, 4, and 9 of the 14-day experimental period; control group, orally gavaged with PBS for 14 days. Adjusted *P*-values were calculated using two-way ANOVA with Tukey’s multiple comparison tests in R studio version 4.4.1. Mean values in the same row with different superscripts are statistically different (*P* < 0.05). HMD, Human Metabolome Database.

In the cecum, a total of 279 metabolites were identified across treatment groups, of which 23 metabolite abundances were significantly altered due to BI supplementation. The abundance of 14 and 9 metabolites was lower and higher, respectively, in the BI mice compared with those that did not receive BI (Table 5). Pathway analysis of low abundance metabolites revealed enrichment of glycine, serine, and threonine metabolism in the cecum, with a trending significance (FDR = 0.07). Only six metabolites were affected due to HMO supplementation. For example, higher levels of 3-hydroxytetradecenoylcarnitine (C14:1-OH), C14:2 acylcarnitine, C18 acylcarnitine, and C5 acylcarnitine were observed in HMO mice compared with those that did not receive HMO, while the abundance of citrulline and ornithine was lower. No BI × HMO interactions were detected.

**TABLE 5:** Cecal metabolites that are differentially regulated in BI mice (BI and BI + HMO groups) compared with those not receiving BI (HMO and control groups) (*n* = 10–14 mice/group)^*a*^

Metabolite	Compound class	BI		No BI		Adjusted *P*-value		
		HMOs	No HMOs	HMOs	No HMOs	HMOs	BI	BI × HMOs
Upregulated								
2-Hydroxy-3-methylvaleric acid	Organic acids	0.915 ^b^	0.404 ^ab^	−0.695 ^a^	−0.623 ^a^	0.791	<0.001	0.590
2-Oxoisocaproic acid	Organic acids	0.923 ^b^	0.147 ^ab^	−0.569 ^a^	−0.501 ^a^	0.780	0.005	0.515
GCA	Organic acids	0.993 ^b^	0.125 ^ab^	−0.552 ^a^	−0.566 ^a^	0.714	0.003	0.515
Hypoxanthine	Nucleobases	0.300 ^ab^	0.780 ^b^	−0.701 ^a^	−0.380 ^ab^	0.776	0.005	0.848
Indolelactic acid	Indole derivatives	0.926 ^b^	0.421 ^b^	−0.671 ^a^	−0.676 ^a^	0.791	<0.001	0.590
PC aa C32:2	Glycerophospholipids	0.327 ^ab^	0.809 ^b^	−0.680 ^a^	−0.456 ^a^	0.780	0.003	0.762
PC aa C38:6	Glycerophospholipids	−0.018 ^ab^	1.015 ^b^	−0.479 ^a^	−0.518 ^a^	0.708	0.008	0.515
PC aa C40:6	Glycerophospholipids	0.027 ^ab^	0.928 ^b^	−0.464 ^a^	−0.490 ^a^	0.725	0.017	0.515
Xanthine	Nucleobases	0.784 ^b^	0.175 ^ab^	−0.644 ^a^	−0.315 ^ab^	0.841	0.021	0.515
Downregulated								
Asparagine	Amino acid	−0.663 ^a^	−0.559 ^a^	0.974 ^b^	0.248 ^ab^	0.780	<0.001	0.515
Cystathionine	Amino acid	−0.559 ^a^	−0.388 ^a^	0.709 ^a^	0.239 ^a^	0.837	0.025	0.590
Glutamine	Amino acid	−0.652 ^a^	−0.663 ^a^	1.107 ^b^	0.209 ^ab^	0.667	<0.001	0.515
Glycine	Amino acid	−0.516 ^a^	−0.564 ^a^	0.986 ^b^	0.095 ^ab^	0.714	0.004	0.515
Histidine	Amino acid	−0.585 ^a^	−0.522 ^a^	0.941 ^b^	0.166 ^ab^	0.780	0.003	0.515
Inosine	Nucleoside	−0.964 ^a^	−0.450 ^a^	0.748 ^b^	0.667 ^b^	0.791	<0.001	0.590
LysoPC a C18:1	Glycerophospholipids	−0.571 ^a^	−0.317 ^a^	0.648 ^a^	0.240 ^a^	0.914	0.048	0.590
LysoPC a C18:2	Glycerophospholipids	−0.663 ^a^	−0.342 ^ab^	0.684 ^b^	0.322 ^ab^	0.968	0.015	0.590
LysoPC a C26:1	Glycerophospholipids	−0.327 ^a^	−0.617 ^a^	0.723 ^a^	0.222 ^a^	0.780	0.025	0.822
Methionine sulfoxide	Amino acid -related	−0.548 ^a^	−0.577 ^a^	1.047 ^b^	0.078 ^ab^	0.667	0.002	0.515
PC aa C28:1	Glycerophospholipids	−0.420 ^a^	−0.539 ^a^	0.595 ^a^	0.364 ^a^	0.820	0.025	0.900
Threonine	Amnio acid	−0.532 ^a^	−0.485 ^a^	1.118 ^b^	−0.102 ^a^	0.398	0.004	0.515
Uridine	Pyridine	−0.993 ^a^	−0.758 ^a^	0.980 ^b^	0.770 ^b^	0.968	<0.001	0.565
Xanthosine	Nucleobases	−0.359 ^a^	−0.572 ^a^	0.280 ^a^	0.651 ^a^	0.914	0.031	0.595

^*a*^ Values represent the normalized abundance levels and may contain negative values due to normalization (median and pareto scaling). All data processing and normalization were performed using MetaboAnalyst 6.0 software with default parameters. Treatment groups: HMO (human milk oligosaccharides) group, orally gavaged with pooled 2′-fucosyllactose, lacto-N-tetraose, and 3′-sialyllactose at 15 mg/day (5 mg/HMO) for 14 days; BI group, orally gavaged with *Bifidobacterium longum* subsp. *infantis* ATCC 15697 (1 × 10^9^ CFU/d) on days 1, 4, and 9 of the 14 d experimental period; BI + HMO group, orally gavaged with HMO for all 14 days and BI on days 1, 4, and 9 of the 14-day experimental period; control group, orally gavaged with PBS for 14 days. Adjusted *P*-values were calculated using two-way ANOVA with Tukey’s multiple comparison tests in R studio version 4.4.1. Mean values in the same row with different superscripts are statistically different (*P* < 0.05). HMD, Human Metabolome Database.

## DISCUSSION

Interactions between diet and the gut microbiome play a major role in early infant growth and development (38–40). However, studies on the interactive effects of HMOs and BI in shaping the immune system and metabolic functions during early life are lacking. This preclinical study revealed that BI and HMO exert distinct individual effects on the immune cell composition of the MLN and spleen, the large intestinal transcriptome, and the cecal and serum metabolic profiles. Interestingly, we observed unique BI × HMO interactions in each compartment that differed from the individual effects. For example, the relative abundance of 36 serum metabolites was significantly impacted due to the BI × HMO interaction (Table 4). Although the list of metabolites in Table 4 was also affected by the main effects of BI and/or HMO, the metabolites were interpreted as a BI × HMO interaction because the main effects might misrepresent the actual relationship.

In a recent study, germ-free mice with a BI-positive microbial community exhibited increased villi height and small intestine crypt depth compared with the group with BI-negative microbiota (20). Herein, BI-treated mice exhibited increased ileal crypt depth, suggesting a direct influence of BI on gut morphology and host nutrient absorption. However, in microbially colonized piglets, BI supplementation decreased (*P* = 0.001) ileal crypt depth (32), suggesting the influence of BI on gut morphology is dependent on species. Similarly, colonized piglets fed formula with 2′-FL, *B. infantis* strain Bi-26 (BI-26), or 2′-FL + BI-26 reported that 2′-FL increased (*P* = 0.040) and BI-26 decreased (*P* = 0.001) ileal crypt depth (29). Our novel findings demonstrate that mono-colonization of BI induces host responses independent of HMO supplementation.

Previous studies have shown that BI and HMOs individually influenced the immune cell composition, particularly in the spleen. Germ-free mice mono-colonized with BI at days 7 and 10 of life exhibited a higher percentage of neutrophils, memory, and naïve CD4^+^ T cells in the spleen at day 14 of age compared with the control group (41). In another study, germ-free mice administered a pool of HMOs for 14 days exhibited a higher percentage of CD4^+^ T cells in the spleen at 50 days of age (7). Therefore, it was unsurprising to observe the individual effects of BI and HMO on the spleen and MLN immune cell composition in the current study. Nyangahu et al. (41) reported that mono-colonization of BI in neonatal mice decreased splenic inflammatory monocytes (Ly6C-high) compared with control (41). In the current study, mice given BI exhibited a higher percentage of monocytes in MLN, while the increase in the splenic monocyte population approached statistical significance. Furthermore, the BI + HMO group had a higher proportion of splenic monocytes than the HMO group, suggesting that BI can directly influence local and systemic monocyte populations. Further characterization of BI effects on Ly6C-high and Ly6C-low monocytes in the spleen and MLN might help understand the role of BI in the inflammatory response. In conventional piglets fed formula with 2′-FL, BI-26, or 2′-FL + BI-26, serum immune cell populations were unaffected by treatment. However, serum cytokines, interleukin (IL)−1RA, IL-1β, IL-12, and IL-18 were all higher (*P* < 0.05) in the 2′-FL group than in the piglets fed formula alone. Additionally, peripheral blood mononuclear cells (PBMC) isolated from piglets fed 2′-FL and stimulated *ex vivo* with lipopolysaccharide secreted higher concentrations of interleukin-1 receptor antagonist (IL-1RA) and tended to secrete more IFN-γ than piglets fed formula alone (29), suggesting immune responsiveness is altered following HMO and/or BI inoculation.

BI impacts metabolite abundances and alters immune response (18). For example, in *an in vitro* study, BI metabolite indole-3-lactic acid reduced IL-8 responses, a neutrophil attractant, in a human intestinal cell line (18). This may explain why mice that received BI exhibited a lower proportion of neutrophils in MLN and a higher cecal concentration of indoleacetic acid. Our laboratory reported that a BI*-*positive microbial community increased percentages of splenic dendritic and B cells in a germ-free mouse model (20). In the current study, mice treated with BI or BI + HMO exhibited a higher proportion of splenic dendritic and B cells, suggesting that BI’s immunomodulatory effect on systemic dendritic and B cell populations is independent of other gut commensals.

Recent studies have shown that BI modulates immune responses by influencing T cell subsets. For example, mono-colonization with BI enhanced antigen-specific CD4^+^ T cells to Bacille Calmette-Guerin vaccine in germ-free mice (41). In another study, BI protected against inflammation in the 2,4,6-trinitrobenzene sulfonic acid (TNBS)-induced colitis model in colonized mice by suppressing MLN Th1 and Th17 responses and promoting regulatory T cell responses (42). Additionally, an increase in specific IgG, IgG1, and IgM antibody responses against pneumococcal conjugate vaccine in germ-free mice colonized with *Bifidobacterium* was observed (43). Therefore, it is not surprising to observe the influence of BI on T cell subsets in the current study. Fu et al. reported that colonized mice receiving BI*-*modulated shrimp tropomyosin-induced allergen exhibited an induction of regulatory T cells via dendritic cells in MLN and spleen (44). Konieczna et al. argued that monocyte-derived dendritic cells are responsible for the differentiation of CD4^+^ T cells into regulatory T cells (45). However, in the present study, mice treated with BI did not exhibit a higher proportion of dendritic cells in MLN. In contrast, they exhibited a higher proportion of monocytes, CD4^+^ T cells, and regulatory T cells in MLN, further supporting the association of BI and regulatory T cell polarization. It was shown that PBMCs from colonized piglets co-administered with HMO (2′-FL) and BI exhibited a distinct anti-inflammatory cytokine profile following stimulation with LPS, compared with the effects of each component alone (29). Similarly, in our study, co-administration of BI and HMO resulted in unique immunomodulatory effects on immune cell populations in the MLN and spleen, distinct from individual effects, particularly on CD4^+^ T cell subsets in MLN. This is noteworthy because dendritic cells in gut mucosa present antigen to naive CD4^+^ T cells to differentiate into effector T-helper cells (Th1, Th2, Th17, and T-regs) (46). This protects the host from external pathogens and allergens, as well as balancing immune responses.

A recent study has shown that stool from breastfed infants colonized with BI EVC001 suppressed the polarization of naive CD4^+^ T cells from a healthy adult donor to Th2 cells *in vitro* (47). Similarly, in the current study, BI + HMO-treated mice exhibited a significantly lower proportion of MLN Th2 cells than the BI group. Zuo et al. reported BI feeding at high doses (6 × 10^8^ CFU/day for 3 weeks) increased MLN Th1 and Th17 cells in the colonized mouse gut (42). However, in the same study, BI ameliorated intestinal inflammation by reducing the Th1 and Th17 responses after colitis induction, indicating that BI modulation of Th1 and Th17 is context dependent. In another study, oral administration of BI CGMCC313-2 (0.2 mL/day) modulated inflammatory responses in mice with allergic asthma by increasing and decreasing Th1- and Th2-related cytokine levels in lung tissue, respectively (48). Based on past literature, it is evident that BI can modulate CD4^+^ T cell subsets. In the current study, BI + HMO treatment resulted in a lower proportion of MLN Th1 than in the BI group and a higher proportion of Th17 than the HMO group. Interestingly, the relative abundance of colon ILC1, the innate counterpart to Th1, and ILC2, the innate counterpart to Th2 cells, was higher and lower, respectively, in the BI group compared with the BI + HMO group, highlighting the unique immunomodulatory effects of BI in the presence of HMO. Furthermore, the results of this study highlight the need to evaluate the effects of BI on immune responses in the presence of HMO, particularly in relation to vaccine responses. Rosa et al. reported that oral administration of pooled HMO from human milk to germ-free mice for 14 days reduced the percentage of splenic monocytes and neutrophils and increased MLN plasma cells on day 28 (7). In another study, sialylated HMO (3′-SL and 6′-SL) enhanced Th1 and Th17 responses while reducing Th2 responses in an intestinal cell line model (49). In the same study, no effect of fucosylated (2′-FL and 3′-FL) and neutral (LNT and LNT2) HMOs on immune responses was reported compared with the control. In the current study, we observed that mice given a mixture of HMO (2′-FL, LNT, and 3′-SL) exhibited a lower proportion of MLN Th1 cells. The difference in the results may be due to the HMO composition. The results of this study support the direct immunomodulatory effects of HMO on systemic and gut-related immune cell populations and suggest that these effects may depend on their composition.

A strong association between gut microbiota and circadian rhythms has been reported (50, 51). However, the mechanisms underlying these associations are largely unknown (50). Tian et al. reported that feeding *B. breve* alleviated the circadian rhythm disturbance induced in sleep-deprived colonized mice (51). Feeding of *B. breve* increased fecal GABA and isovaleric abundance, decreased serum purine metabolites, and normalized overexpression of circadian clock genes, e.g., *Per1* and *Per2*, in sleep-deprived models. In the current study, BI modulated the expression of *Per1, Per2,* and *Per3,* across 13 different cell populations within the colon tissue, and serum abundances of GABA, serotonin, and adenosine in BI mice, highlighting the role of BI in circadian clock. However, further studies are needed to understand these modulatory effects on host health.

A recent study reported that feeding *Bifidobacterium* to germ-free mice increased the serum abundance of indole derivatives including indole-3-lactic acid (ILA) (41). Additionally, colonizing germ-free mice with BI-positive fecal samples from breastfed infants resulted in higher abundances of indoxyl sulfate in cecal contents (20). In line with previous results, mice administered BI exhibited higher abundances of indole derivatives such as indole, ILA, and indole-3-propionic acid. Numerous studies have shown that gut bacteria-derived ILA can modulate brain function and immune responses via aryl hydrocarbon receptors (52, 53). Although we did not evaluate behavioral changes, a strong influence of BI on the MLN and spleen immune cell composition was observed. These may further support the association of gut-derived metabolites with immune responses. In a targeted cecal metabolomics study (361 metabolites screened), neonatal germ-free mice administered BI responded by enhancing amino acid, glycerophospholipid, and unsaturated fatty acid biosynthetic pathways (41). In the present study, integration of cecal and serum targeted metabolomics (600 metabolites/matrix screened) revealed enrichment of amino acid-related pathways in BI mice. Enrichment was observed only in the serum, not in the cecal contents. Interestingly, we observed the downregulation of glycine, serine, and threonine metabolic pathways, reaching significance in the cecal contents of mice that received BI, suggesting a transfer of local cecal changes to systemic circulation. The shift in amino acid metabolism enrichment from cecum to serum strongly supports the BI-mediated systemic metabolic effects. These results suggest that BI modulates host amino acid metabolism by converting amino acids, such as tryptophan, into bioactive metabolites and alters the host metabolite profile. Furthermore, our colon single-cell analysis revealed the enrichment of amino acid-related biological processes in the colonocytes of BI mice, suggesting that BI modulates host amino acid metabolism.

Bo et al. reported that feeding *Bifidobacterium pseudolongum* reduced plasma triglycerides by 12% in obese colonized mice (54). In an *in vitro* adipogenesis assay, *Bifidobacterium longum* subsp. *infantis* YB0411 decreased triglyceride accumulation in adipocytes (55). In a multi-omic study, feeding of fermented milk containing *Bifidobacterium longum* 070103 reduced serum low-density lipoprotein cholesterol, total cholesterol, and triglycerides in high-fat diet-induced colonized mice (56). In comparison, probiotic supplementation containing three bifidobacterial species (*B. animalis* subspecies *lactis* MB 2409, *B. bifidum* MB 109B, and *B. longum* subspecies *longum* BL04) reduced low-density lipoprotein cholesterol and total cholesterol, not triglycerides, in dyslipidemic children (57). However, Aoki et al. reported that feeding of *Bifibacterium* species to obese colonized mice did not affect blood triglycerides (58). The discrepancy in the results of the studies above may be attributed to the strain-specific effect of *Bifibacterium* or dose on triglycerides or a dependency on other gut commensals. In our study, we observed a low UCell enrichment score for monoacylglycerol catabolic processes in colonic adipocytes and reduced levels of serum triglycerides and diacylglycerols in BI mice. This suggests that BI contributes to lower serum triglyceride levels by increasing triglyceride uptake and storage in adipocytes. Given that the effects of bifidobacteria on triglycerides are strain-specific and context-dependent, the influence of BI on triglycerides in obese models may be beneficial and translate to human health.

Herein, the effects of HMO on the serum metabolites were opposite to that of BI. Amino acid metabolic pathways and different types of triglycerides were downregulated in BI mice. In contrast, the same pathways and triglycerides were upregulated in the HMO mice. Although the reasons remain unknown, unfermented HMO may adhere to lectin receptors of host cells (59) and trigger metabolic shifts. Furthermore, a unique BI × HMO interaction was observed with respect to the serum metabolome profile, with 45 metabolites differentially regulated between the BI group and the BI + HMO group. Pathway enrichment analysis revealed that glyoxylate and dicarboxylate metabolism, and the citric acid cycle pathway, were enriched in the serum of the BI group compared with the BI + HMO group. This dichotomous response may be related to carbon source availability. For example, organisms rely on alternate carbon sources, such as amino acids or host-derived metabolites, for growth. To efficiently utilize carbon compounds, glyoxylate and dicarboxylate pathways can contribute to energy production and upregulate the citric acid cycle to provide intermediate metabolites for energy production. Thus, in the presence of HMOs, the organism shifts metabolically toward fermentative pathways. This may explain the enrichment of glyoxylate and dicarboxylate metabolism and citric acid cycle in the BI group compared with the BI + HMO group, highlighting BI adaptation to different environments.

In summary, mono-colonization with BI elicited distinct host responses across different organ sites compared with the effects of HMO supplementation and BI × HMO interactions. One possible reason is that germ-free mouse models lack competitive microbiota, likely allowing BI to colonize and robustly drive metabolic and immune modulatory outcomes. While BI and HMO exerted distinct effects on host responses, BI × HMO interactions induced unique non-additive host responses, highlighting the complex interplay of microbiota and diet interaction on host health. Overall, the results of this study highlight the importance of considering host-microbe-diet interaction effects when designing strategies to modulate infant health during early life.

Our study has several limitations. Using the germ-free mouse model eliminates microbial competition and cross-feeding and consequently enhances the effect of BI. The model may also mask the effects of HMOs on BI within diverse microbial environments. Furthermore, germ-free mice have an underdeveloped immune system compared with conventional mice, which may restrict the generalization of the findings. Though BI alone and in combination with HMOs had effects in this mouse model, those effects may not be specific to BI and may result from the administration of any bacteria, regardless of genera/species/strain, in these mice. Finally, although we evaluated all three different classes of HMO, using the full spectrum of HMOs might provide more translational insights into host development.

## MATERIALS AND METHODS

Healthy, C57BL/6J male and female germ-free mice, weaned at 21 days, were randomly assigned to four groups (*n* = 10–14/group: HMO, BI, BI + HMO and control [no HMO or BI]) after blocking for male/female ratio and weights across the groups. HMO and BI + HMO groups were orally gavaged with three HMO (2′-fucosyllactose, lacto-N-tetraose and 3′-sialyllactose; DSM-Firmenich, Maastricht, Netherlands) at 15 mg/day (5 mg/HMO) for 14 days. BI and BI + HMO groups were orally gavaged with BI ATCC 15697 (1 × 10^9^ CFU/day) on days 1, 4, and 9 of the 14-day experimental period, while the control group received sterile 1× PBS (100 µL) throughout the experimental period. At the end of the study period, mice were exposed to isoflurane (1%–5%) until unconscious, and blood was collected via retroorbital bleeding before euthanasia by cervical dislocation. Spleen and MLN were processed for immune cell composition. Formalin-fixed tissues were used for histomorphometric analyses, while colon samples were taken for single cell RNA sequencing. Fecal samples were used to measure BI colonization by PCR (60). Serum and cecal contents were used for metabolomics analyses. For detailed methods and statistical analyses, see the supplemental methods.

## Data Availability

Single-cell RNA sequencing data are publicly available in the Gene Expression Omnibus (GEO) repository accession GSE310711. The tool used for data analysis is available at https://github.com/jamesjcai/scGEAToolbox.

## SUPPLEMENTAL MATERIAL

Figure S1 msystems.00392-26-s0001.pdf Confirmation of germ-free status of treatment groups.

Figure S2 msystems.00392-26-s0002.pdf Molecular validation of germ-free status in the treatment groups.

Figure S3 msystems.00392-26-s0003.pdf Effect of BI and HMO supplementation on body weight, organ weight, and gut tissue morphology.

Figure S4 msystems.00392-26-s0004.pdf Effect of BI and HMO supplementation on colonocyte populations.

Figure S5 msystems.00392-26-s0005.pdf Effect of BI and HMO supplementation on non-epithelial cell populations.

Figure S6 msystems.00392-26-s0006.pdf Effect of BI and HMO supplementation on T cell, mast cell, and ILC populations.

Supplemental Text msystems.00392-26-s0007.docx Supplemental methods.

Legends msystems.00392-26-s0008.pdf Supplemental figure legends.

Supplemental Tables msystems.00392-26-s0009.docx Tables S1 to S8.

ASM does not own the copyrights to Supplemental Material that may be linked to, or accessed through, an article. The authors have granted ASM a non-exclusive, world-wide license to publish the Supplemental Material files. Please contact the corresponding author directly for reuse.
