# Supplementary material for: Bifidobacteria infantis and human milk oligosaccharides have independent and synergistic effects on immune response and amino acid metabolism in germ-free mouse models
Source: mSystems. 2026 Jun 15;11(7):e00392-26. doi: 10.1128/msystems.00392-26 (PMC13386997; doi:10.1128/msystems.00392-26)
Supplement: Figure S1 — Confirmation of germ-free status of treatment groups. [file msystems.00392-26-s0001.pdf]

	Control	HMO	HMO + BI	BI
Blood agar	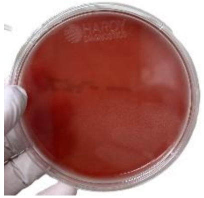	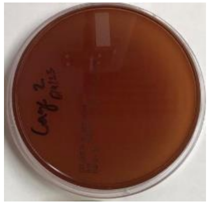	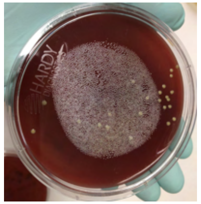	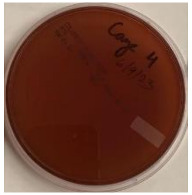
Sabouraud agar	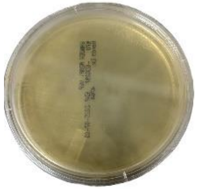	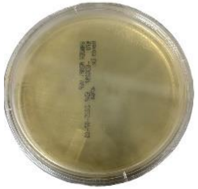	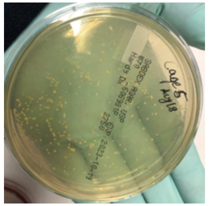	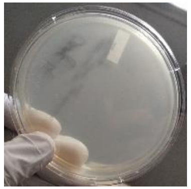
Tryptic soy broth (left) & Thioglycolate broth (right)	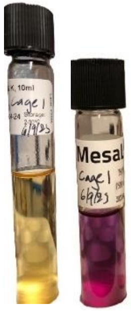	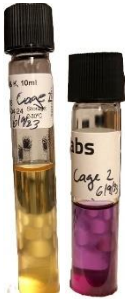	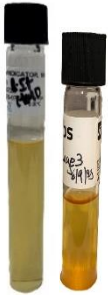	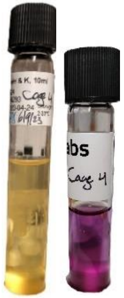
