# Supplementary figures and images for: Bifidobacteria infantis and human milk oligosaccharides have independent and synergistic effects on immune response and amino acid metabolism in germ-free mouse models
Source: mSystems. 2026 Jun 15;11(7):e00392-26. doi: 10.1128/msystems.00392-26 (PMC13386997; doi:10.1128/msystems.00392-26)

A

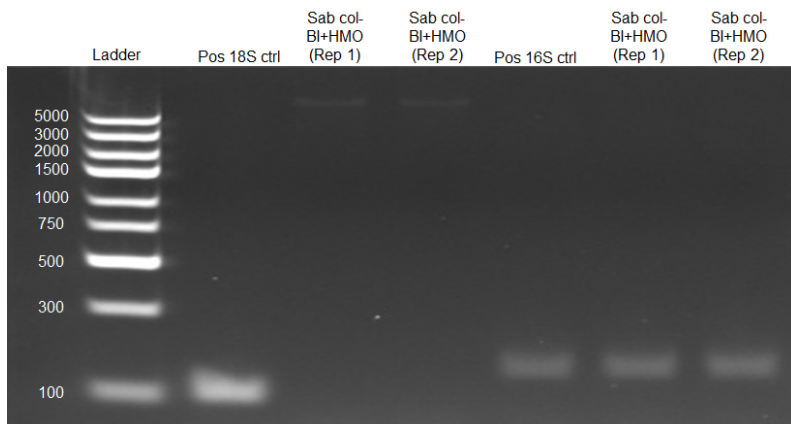

B

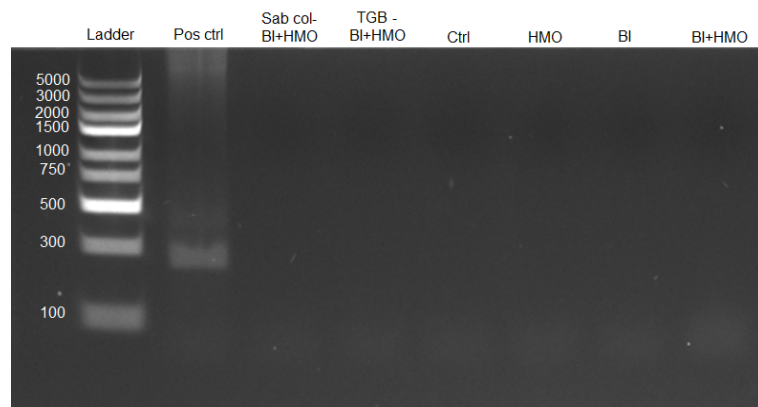

C

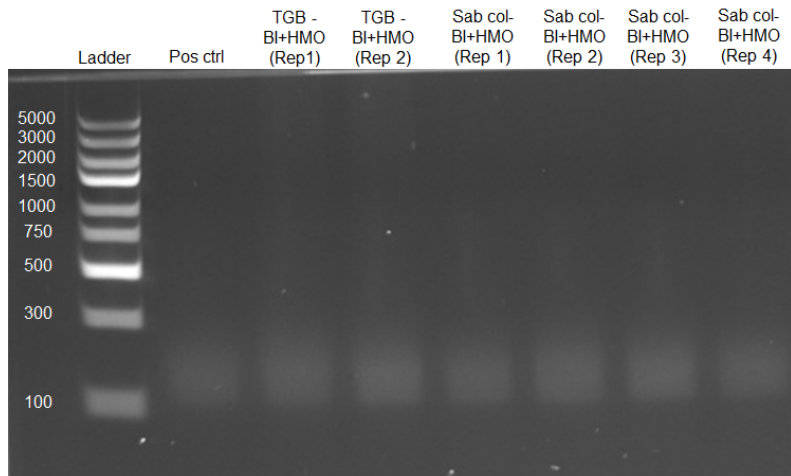

D

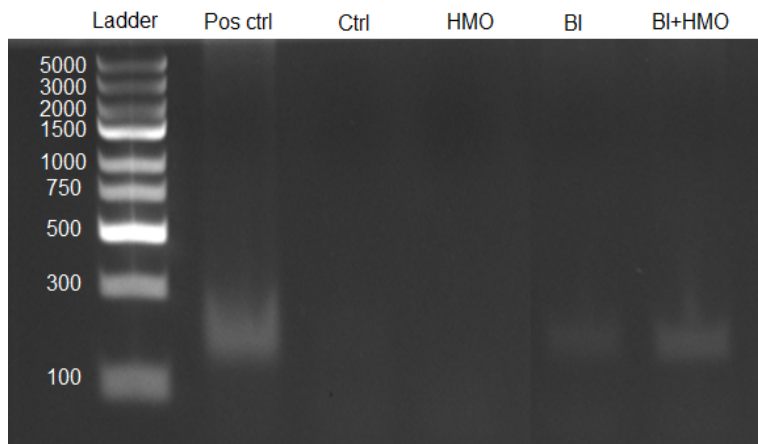

Supplement: Figure S2 — Molecular validation of germ-free status in the treatment groups. [file msystems.00392-26-s0002.pdf]

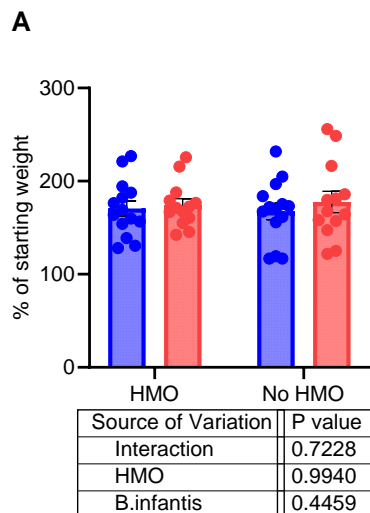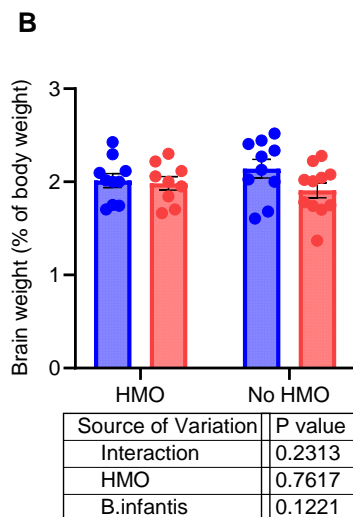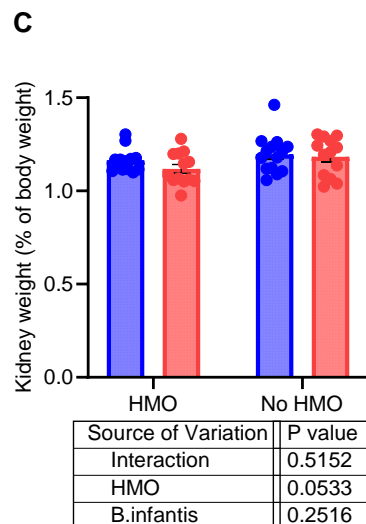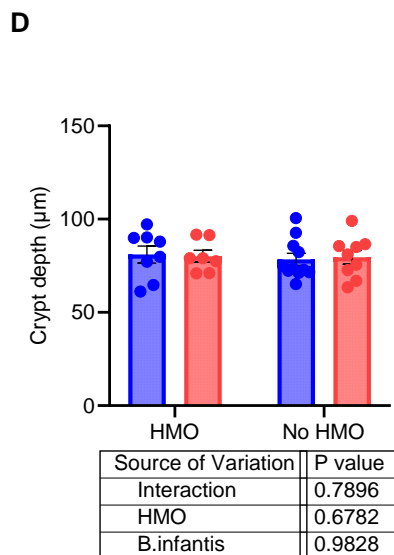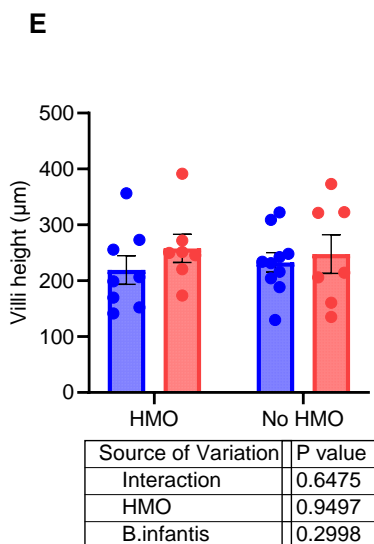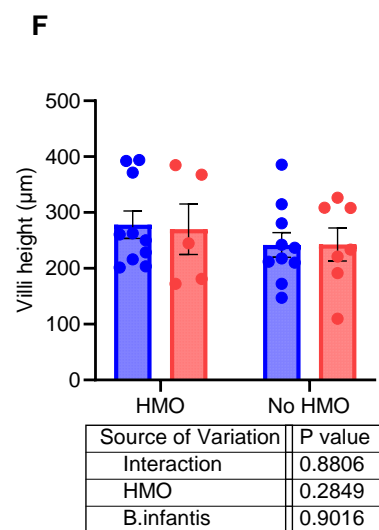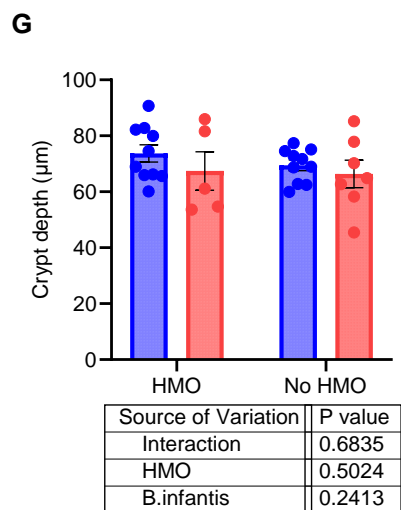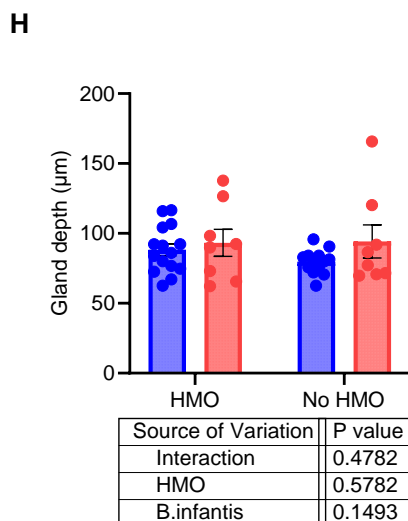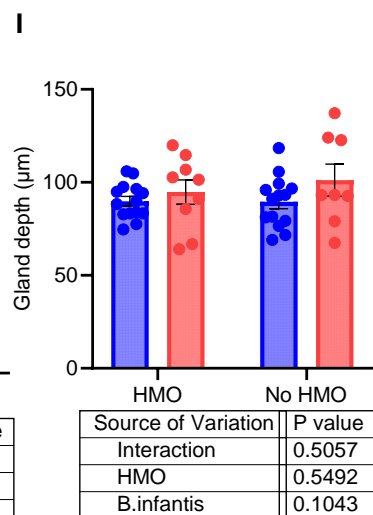

Supplement: Figure S3 — Effect of BI and HMO supplementation on body weight, organ weight, and gut tissue morphology. [file msystems.00392-26-s0003.pdf]

**A** Epithelial cells

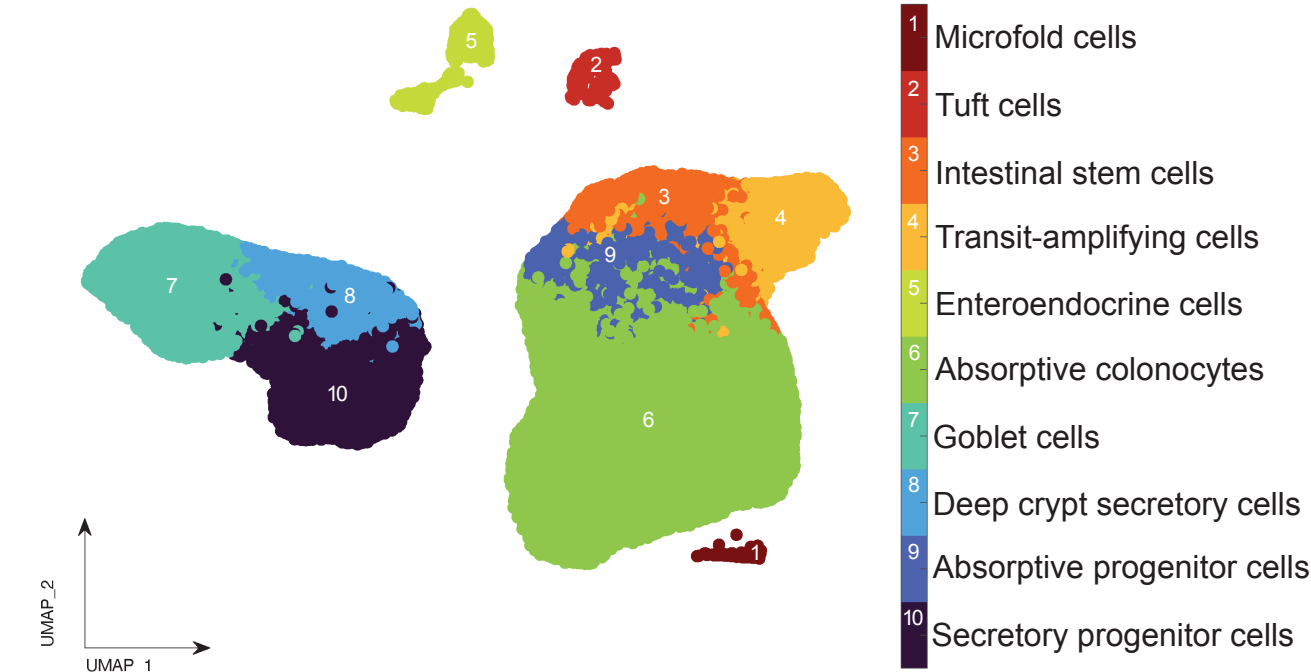

**B**

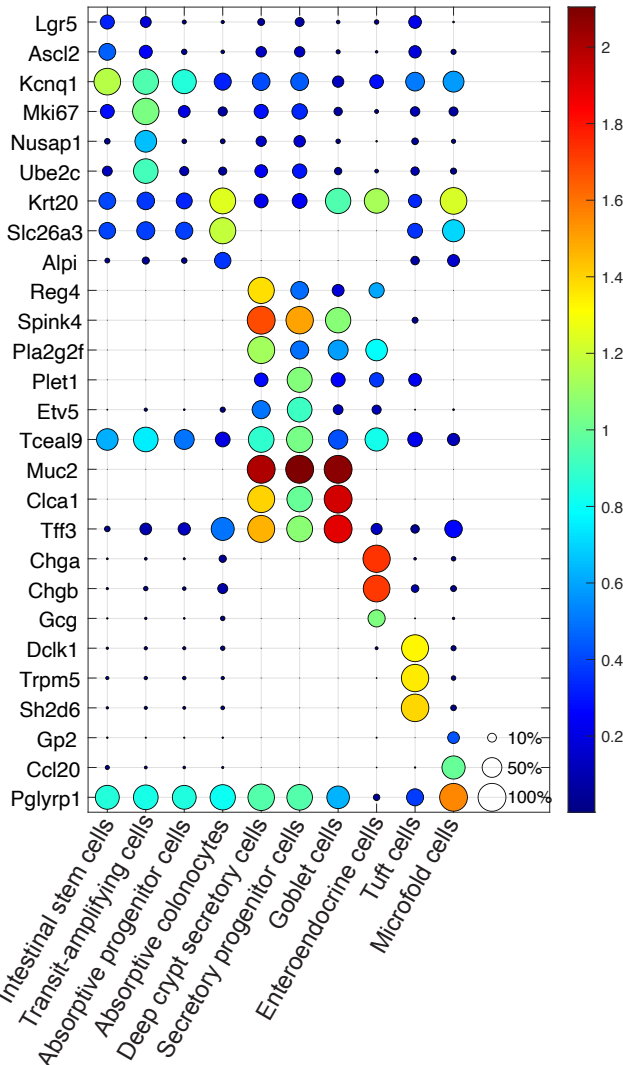

**C**

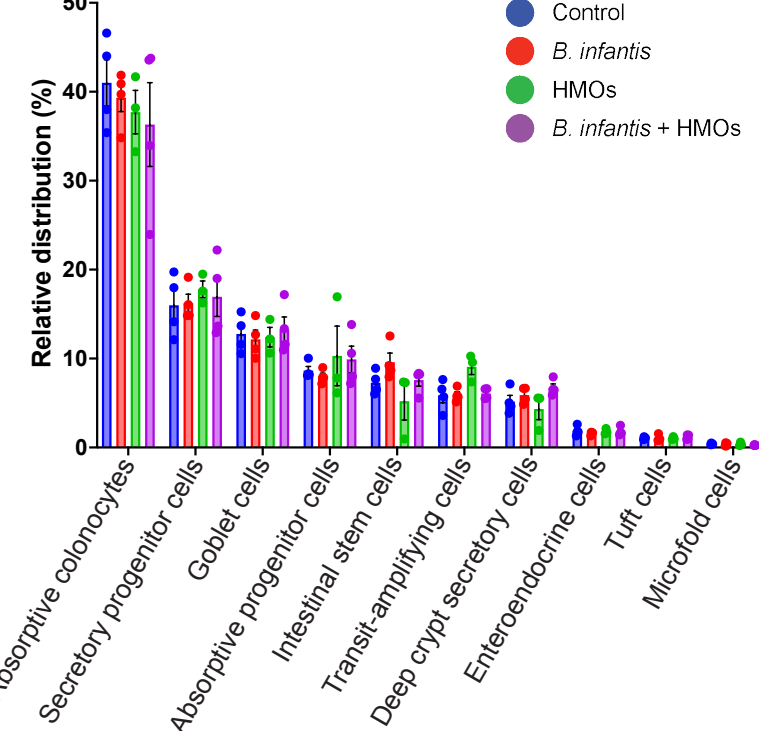

| Source of Variation | P value     |
|---------------------|-------------|
| Interaction         | 0.8190      |
| Cell type           | ****<0.0001 |
| Treatment           | >0.9999     |

Supplement: Figure S4 — Effect of BI and HMO supplementation on colonocyte populations. [file msystems.00392-26-s0004.pdf]

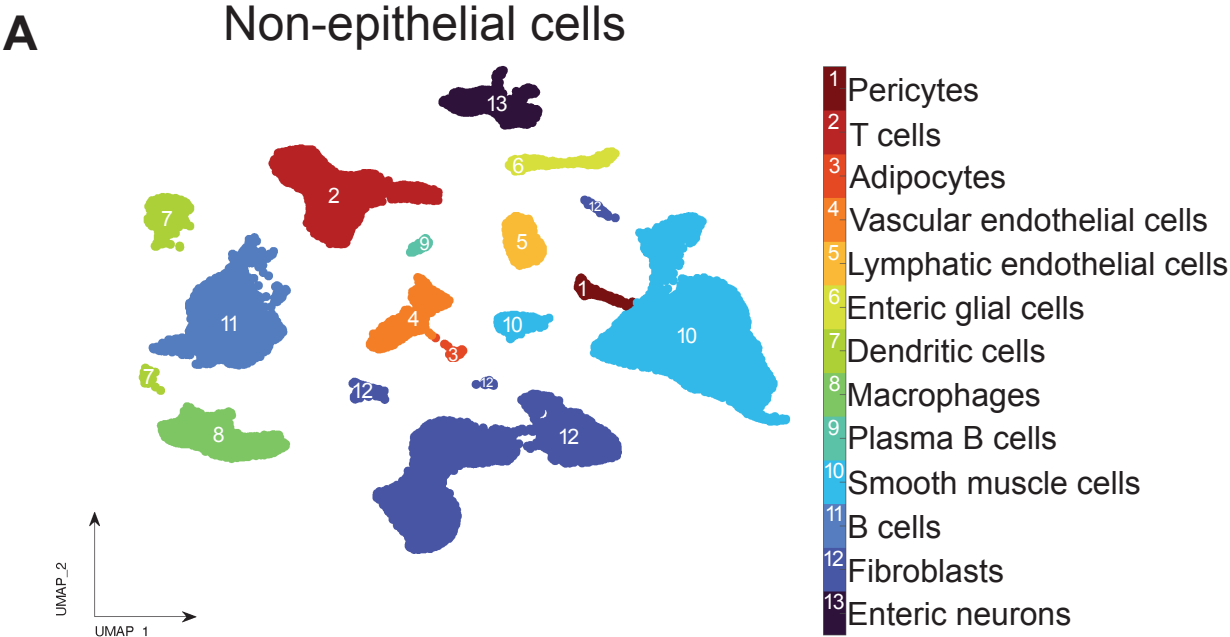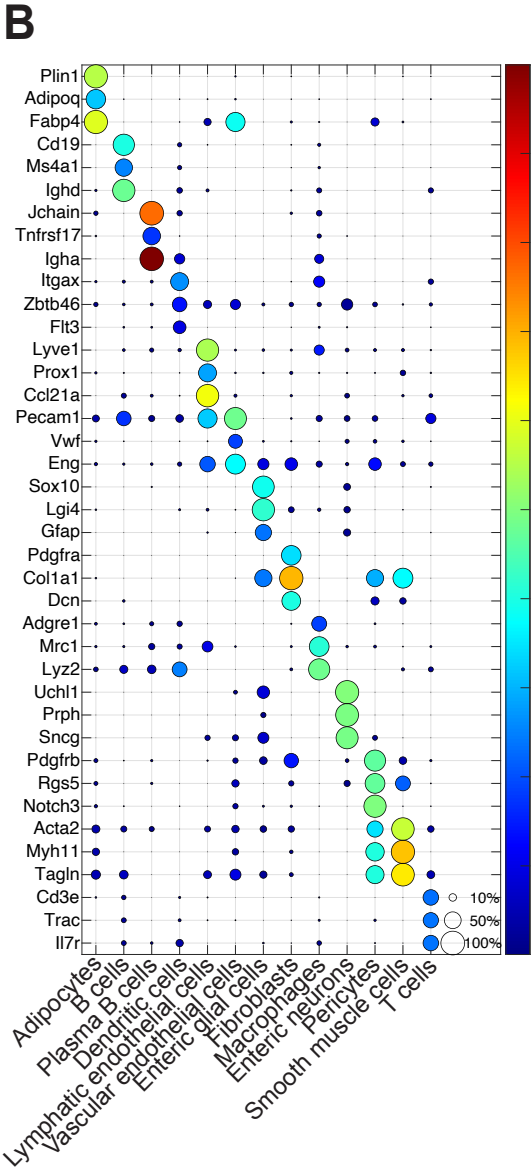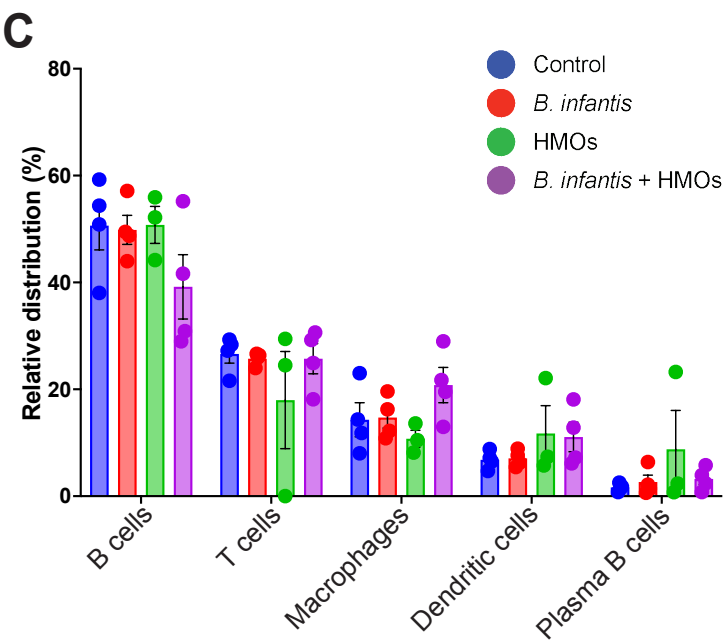

| Source of Variation | P value     |
|---------------------|-------------|
| Interaction         | 0.0943      |
| Cell type           | ****<0.0001 |
| Treatment           | >0.9999     |

Supplement: Figure S5 — Effect of BI and HMO supplementation on non-epithelial cell populations. [file msystems.00392-26-s0005.pdf]

A T cell subtypes

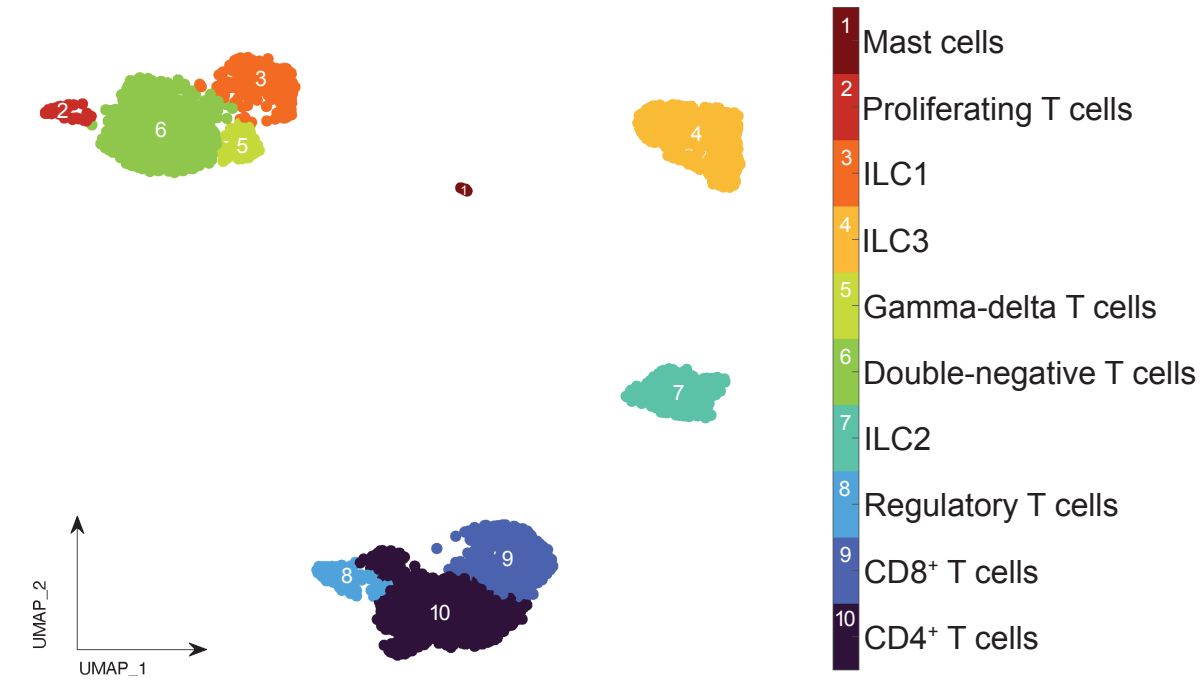

B

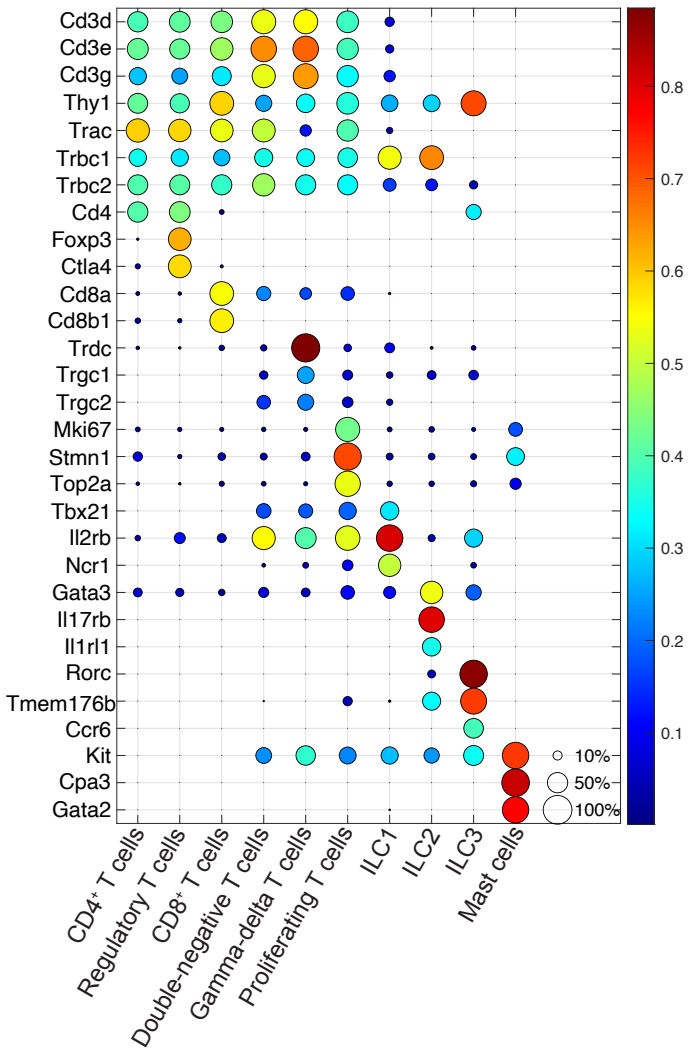

C

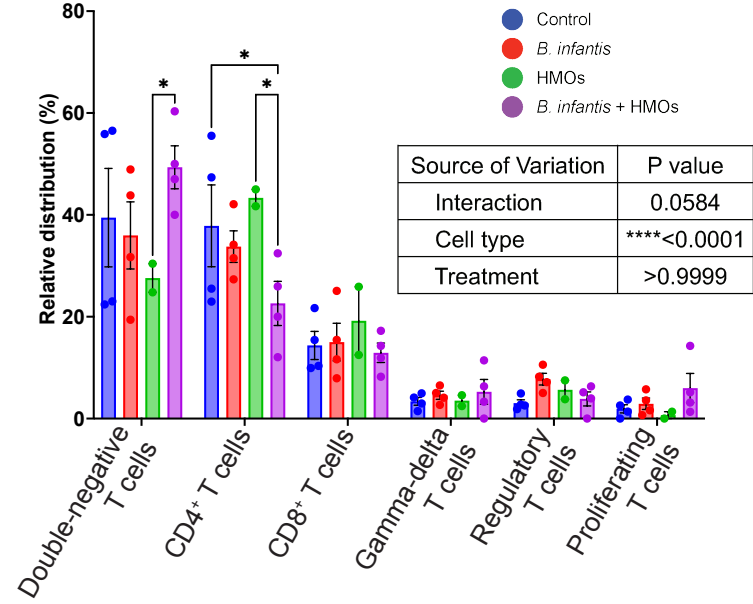

D

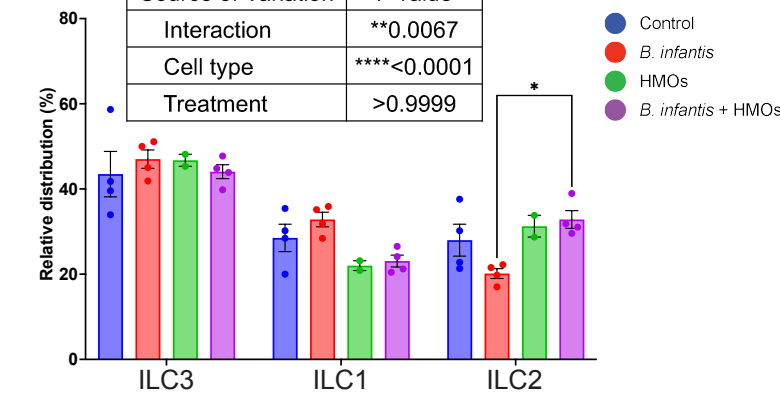

Supplement: Figure S6 — Effect of BI and HMO supplementation on T cell, mast cell, and ILC populations. [file msystems.00392-26-s0006.pdf]
