# Supplementary material for: Bifidobacteria infantis and human milk oligosaccharides have independent and synergistic effects on immune response and amino acid metabolism in germ-free mouse models
Source: mSystems. 2026 Jun 15;11(7):e00392-26. doi: 10.1128/msystems.00392-26 (PMC13386997; doi:10.1128/msystems.00392-26)
Supplement: Supplemental Text — Supplemental methods. [file msystems.00392-26-s0007.docx]

**Experimental design**

The animal studies were approved by the Institutional Animal Care and Use Committee at the University of Arkansas for Medical Sciences. Healthy, C57BL/6J male and female germ-free mice, weaned at 21 d, were transferred to a germ-free Innovive mouse cage system (San Diego, CA) and were maintained under 12-h light cycle (lights on at 0700h and off at 1900h) at 23°C with *ad libitum* access to sterile food (ENVIGO 2020SX) and water.

Mice were randomly assigned to four groups [n = 10-14/group: HMO,BI, *B.* BI+HMO and control (no HMO or BI)] after blocking for male/female ratio and weights across the groups. HMO and BI+HMO groups were orally gavaged with three HMO (2′-fucosyllactose, lacto-N-tetraose and 3′-sialyllactose; DSM-Firmenich, Maastricht, Netherlands) at 15 mg/d (5 mg/HMO) in 100 µL (suspension was prepared using 1X PBS and sterilized with 0.2 µm filter) for 14 d. BI and BI+HMO groups were orally gavaged with BI ATCC 15697 (1x10^9 CFU/d) on days 1, 4, and 9 of the 14 d experimental period, while the control group received sterile 1X PBS (100 µL) throughout the experimental period. To confirm the germ-free status of the control and HMO mice, microbiological culture in agar plates and liquid media was performed. Samples (a mixture of feed, bedding, and water) as well as swabs from the cage surface of each group cage, collected during the last week of the trial, were cultured in blood agar, Sabouraud agar plates, and thioglycolate and tryptic soy broth and incubated aerobically for 24 h at 37°C. At the end of the study period, mice were exposed to isoflurane (1%–5%) until unconscious, and blood was collected via retroorbital bleeding before euthanasia by cervical dislocation. Blood samples were spun down at 3,000 rpm for 10 min at 4°C to collect serum, which was stored at 80°C until further analysis. Tissues were collected from each mouse for analyses described below.

**Flow cytometry analysis**

Spleen and MLN were removed, placed into ice-cold Roswell Park Memorial Institute (RPMI) media (Invitrogen, catalog #11875093) with 20% FBS, and processed to obtain single-cell suspensions as described previously^1^. Single-cells were suspending in the flow cytometry staining buffer (ThermoFisher, catalog #00-4222-26) and counted using Vi-Cell Blu Cell Viability Analyzer (Beckman Coulter, catalog #C19196). Approximately 2x10^6 live spleen cells and 6x10^5 live MLN cells per sample were stained with a live/dead marker, followed by staining with specific antibodies^1^. Cells were fixed in 1% PFA and data were acquired using a BD LSR Fortessa at the University of Arkansas for Medical Sciences flow cytometry core facility. The acquired flow data were analyzed using FlowJo software (v10.2). Monocytes (CD90^–^/CD19^–^/CD11c^–^/LY6C^+^), neutrophils (CD90^–^/CD19^–^/CD11c^–^/LY6G^+^), macrophages (CD90^–^/CD19^–^/CD11c^–^/CD64^+^), B cells (CD90^–^/CD19^+^), dendritic cells (CD19^–^/CD11c^+^), helper T cells (CD90^+^/T cell alpha-beta receptor^+^/CD4^+^), cytotoxic T cells (CD90^+^/T cell alpha-beta receptor^+^/CD8^+^), innate lymphoid cells (ILC) 1 (CD90^+^/T cell alpha-beta receptor^–^/T-BET^+^), ILC2 (CD90^+^/T cell alpha-beta receptor^–^/GATA3^+^), ILC3 (CD90^+^/T cell alpha-beta receptor^–^/RORGAMMA^+^), T helper type 1 cells (Th1) (CD90^+^/T cell alpha-beta receptor^+^/CD4^+^/T-BET^+^), Th2 (CD90^+^/T cell alpha-beta receptor^+^/CD4^+^/GATA3^+^), Th17 (CD90^+^/T cell alpha-beta receptor^+^/CD4^+^/RORGAMMA^+^), and regulatory T cells (CD90^+^/T cell alpha-beta receptor^+^/ CD4^+^/FOXP3^+^) were identified based on CD markers and gating with parent cell population. See Table **S1** for antibody details.

**Histomorphometric analysis**

After recording the length of the small intestine, large intestine, and the weight of the cecum with contents, 2 cm segments from the proximal (duodenum), middle (jejunum), and distal (ileum) portions of the small intestine, along with the distal portion of cecum, were fixed in 10% formalin for histological analysis. After measuring the length of the colon, the tissue was divided into three parts. The middle part of the colon from each mouse was cut open and rinsed with Ca and Mg-free 1X PBS to remove the remaining gut contents. After rinsing, tissue was blotted to remove excess PBS, weighed (25-35 mg), and flash-frozen using liquid nitrogen before storing at -80°C for single-cell RNA sequencing (scRNA-seq). Additionally, cecal contents collected were flash frozen using liquid nitrogen and stored at -80°C until further analysis.

Formalin-fixed tissues were, paraffin-embedded, cut to 5-micron thickness, placed onto glass slides, and stained with hematoxylin and eosin. A board-certified pathologist performed the morphological analysis (intestine crypt depth, villus height, and cecum gland depth) using Aperio Imaging software as previously described^1^.

**DNA extraction and *B. infantis* qPCR**

DNA was extracted from the fecal samples collected on d 14 using QIAamp Power Fecal Pro DNA Kit (QIAGEN, Hilden, Germany) according to the manufacturer’s protocol. DNA concentration was measured in a Qubit 4^TM^ Fluorometer (ThermoFisher Scientific, Wilmington, DE, USA) using 1X dsDNA HS Assay Kit^TM^ for Qubit. To evaluate the abundance of BI colonization, we used a BI-specific primer set targeting the Blon_0915 region in a probe-based qPCR analysis as previously described^2^. PCR primers/probe sequences and cycling parameters can be found in **Table S2**.

**Metabolomics**

Serum and cecal contents were shipped to The Metabolomics Innovation Center (TMIC), University of Victoria, BC, Canada for screening of over 600 metabolites (MEGA assay) in the sample matrices. The assay was performed by a combination of liquid chromatography (LC) for separating molecules and tandem mass spectrometry (MS/MS) for detecting and quantifying the metabolites in the sample matrix.

**Single-cell RNA sequencing sample processing**

Flash frozen mouse colon samples were minced on dry ice and fixed according to 10X Genomics Tissue Fixation and Dissociation for Chromium Fixed RNA Profiling protocol (CG000553, Rev B). All samples were fixed for 18 h and kept on ice while cell concentration was determined with an automated cell counter (Nexcelom Cellometer K2) using ViaStain AOPI Staining Solution (Nexcelom, CS2-0106-5mL). Samples were subsequently processed for the Fixed RNA Profiling (Flex) assay according to the 10X Genomics Chromium Fixed RNA Profiling Reagent Kits for Multiplexed Samples user guide (CG000527, Rev E). Four samples were combined per pool (4 pools total). A 1:80 dilution of the sample pools was tested for quality control utilizing an Agilent Bioanalyzer High Sensitivity chip (Agilent, 5067-4626, Santa Clara, CA). Library quantification was performed with the KAPA Library Quantification Kit for Illumina Platforms (Roche, KK4824). Libraries were pooled based on the 10X Genomics Chromium Connect Pooling Worksheet (CG000156) and prepared for Illumina sequencing platforms as recommended by 10X Genomics. Single-cell libraries were sequenced by Admera Health (South Plainfield, NJ) using a NovaSeq X Plus sequencer (Illumina, San Diego, CA). For the mapping of transcripts and cells, sample demultiplexing, barcode processing, and unique molecular identifier (UMI) counts were performed using the 10X Genomics pipeline CellRanger v.8.0.0^3^. Sequences were aligned to the mouse reference genome (GRCm39-2024-A) using the Chromium mouse transcriptome probe set v1.0.1 to generate the gene-barcode matrix.

**Single-cell RNA sequencing data quality control and cell annotation**

A gene-by-cell matrix was generated from the filtered feature gene-barcode matrix utilizing the scGEAToolbox (v25.7.1) from GitHub: (<https://github.com/jamesjcai/scGEAToolbox>)^4^. Specifically, to filter out low-quality cells, cells expressing: 1) less than 500 UMIs or more than the upper 99% quantile of UMIs per sample; 2) greater than 25% mitochondrial reads; and/or, 3) less than 200 unique genes, were excluded utilizing the QC filtering feature of the scGEAToolbox. Sample matrices were merged, doublets (via scrublet v0.2.3) and ambient RNA contamination (via decontX v1.2.0)^5^ were removed. Dimensionality reduction of expression matrices was performed using a Seurat wrapper function (*npcs* = 50) to generate a 2D uniform manifold approximation and projection (UMAP)^6^ embedding (*dims* = 30). Cell clustering was then conducted by the same Seurat wrapper function utilizing the Leiden^7^ clustering algorithm (*r* = 4). Following cell clustering, cell type annotation was performed with each cluster independently, after which clusters of cells with the same annotation were subsequently merged. These analyses were carried out utilizing the embedding and cell clustering wrapper function features of scGEAToolbox in an automatic manner. PanglaoDB, containing a community-curated cell-type marker database, along with well-known marker genes, were utilized for identifying cell types^8^. Cell populations were initially classified as epithelial cells (*Epcam^+^*) or non-epithelial cells (*Epcam^-^*) populations. After identifying and selecting the epithelial cell populations, we re-performed the embedding and clustering steps before annotating colonocyte subtypes utilizing known marker genes followed by downstream analysis. Similarly, after isolating the non-epithelial cell populations, we repeated the embedding and clustering steps before annotating the mesenchymal (fibroblasts, smooth muscle cells, pericytes, adipocytes), neural (enteric neurons, enteric glial cells), endothelial (vascular, lymphatic), and immune (T cells, B cells, plasma B cells, macrophages, dendritic cells) cell populations followed by downstream analysis. The embedding and clustering process was repeated once more for the T cell population which contained T cells, mast cells, and innate lymphoid cells (ILCs).

**Gene expression and normalization**

Gene expression data were normalized by library size, which scales single-cell gene expression counts, by dividing each gene count by the total expression of its respective cell and multiplying by the mean library size across all cells. This ensured comparability across samples while accounting for differences in sequencing depth. Library size normalization was then followed by a log1p transformation prior to downstream analyses, unless stated otherwise.

**Cell function and activation status scores**

The UCell package in R was used for evaluating the gene signature enrichment scores for a predefined gene set in single-cell datasets^9^. The Molecular Signatures Database (MSigDB) v2023.2 was used as the source of annotated gene sets for analysis. Briefly, the UCell package allowed the estimation of the gene signature enrichment scores, i.e., UCell scores (Mann–Whitney U statistic), for a given predefined gene set based on the expression of mRNA in a respective cell utilizing raw gene counts.

**Statistical analysis**

Histological data, immune cell composition as well as body and organ weights were analyzed by a two-way ANOVA followed by Tukey’s multiple comparison tests in GraphPad Prism Version 10.0. The effect of BI and HMO supplementation and their interaction (BI × HMO) were considered significant with adj P < 0.05.

To investigate the effects of HMO and BI on gene expression, a comprehensive two-way analysis of variance (ANOVA) was performed. The analysis focused specifically on gene expression within any cell type of interest derived from a scRNA-seq dataset. scRNA-seq data were processed using the scGEAToolbox MATLAB package. Initially, the dataset was filtered to remove lowly expressed genes and partitioned to include only cells identified as the specific cell type of interest (e.g., intestinal stem cells) based on cell type annotations. For each gene in a cell type, expression data were extracted from these selected cells. Gene expression values were then normalized using library size normalization, followed by a log1p-transformation to stabilize variance and make the data more suitable for linear modeling. The unique batch identifiers were converted into categorical variables to represent the experimental conditions. For every gene, a two-way ANOVA was conducted to assess the independent and interactive effects of the BI and HMO conditions on gene expression. The experimental conditions, “BI” and “HMO”, were identified from the corresponding batch identifiers, which encoded the specific treatment combinations (e.g., combinations of BI presence/absence and HMO presence/absence). The linear model for the ANOVA was formulated as follows:

*Expression ~* BI *+ HMOs + B.infantis:HMOs*

Here, “Expression” represents the normalized gene expression level, “BI” denotes the presence or absence of the BI condition, and “HMO” indicates the presence or absence of the HMO condition. The interaction term “BI:HMOs” was explicitly included in the model to evaluate if the joint effect of the conditions on gene expression was dependent on the condition interaction. This analysis was performed gene-wise across all genes in the dataset. To enhance computational efficiency, analyses for gene sets larger than 500 were executed in parallel. This model provided p-values associated with the significance of the contribution of each term to changes in gene expression (i.e., the independent effect of HMO, BI, and their interaction). A p-value associated with each of these terms in the expression model was generated. Following the ANOVA tests, the resulting p-values for the main effects of BI or HMO, and their interaction, as well as the complete ANOVA tables, were compiled. These statistical summaries, along with the raw expression data and treatment information for each gene, were then processed and exported. Individual gene expression profiles were extracted and visualized (e.g., using violin or box plots) to observe the effects of the conditions and their associated p-values.

MetaboAnalyst 6.0 software (accessed on January 1, 2025) was employed for metabolite data normalization and pathway analysis. Data was processed by removing features that had more than 50% missing values and were estimated using KNN (feature-wise). Next, data were normalized by the media and scaled by pareto scaling. Normalized metabolite data (cecum and serum) were analyzed by two-way ANOVA in R studio version 4.4.1 (R code can be found in supplemental material) followed by FDR correction. The effect of BI and HMO supplementation and their interaction (BI × HMO) for gene expression analysis and metabolites were considered significant with adjusted P < 0.05. Next, pathway enrichment analysis of differentially abundant metabolites among the groups was performed using MetaboAnalyst 6.0 software and considered significant at FDR < 0.05.

**Funding:** This work was supported by USDA-ARS (6026-51000-012-000D, 6026-10700-001-000D (LY), and USDA-ARS (55-3091-1-015) (LY, SMD and RSC), Texas A&M Institute for Advancing Health Through Agriculture (RSC), at Texas A&M University, Texas A&M Hagler Institute for Advanced Study (SMD), Allen Endowed Chair in Nutrition & Chronic Disease Prevention (RSC) and the Cancer Prevention & Research Institute of Texas (RP230204) (MLS, JJC, SR, RSC).

1. Gurung M, Schlegel BT, Rajasundaram D, Fox R, Bode L, Yao T, et al. Microbiota from human infants consuming secretors or non-secretors mothers' milk impacts the gut and immune system in mice. mSystems 2024; 9:e0029424.

2. Frese SA, Hutton AA, Contreras LN, Shaw CA, Palumbo MC, Casaburi G, et al. Persistence of Supplemented Bifidobacterium longum subsp. infantis EVC001 in Breastfed Infants. mSphere 2017; 2.

3. Zheng GX, Terry JM, Belgrader P, Ryvkin P, Bent ZW, Wilson R, et al. Massively parallel digital transcriptional profiling of single cells. Nat Commun 2017; 8:14049.

4. Cai JJ. scGEAToolbox: a Matlab toolbox for single-cell RNA sequencing data analysis. Bioinformatics 2019; btz830.

5. Yang S, Corbett SE, Koga Y, Wang Z, Johnson WE, Yajima M, et al. Decontamination of ambient RNA in single-cell RNA-seq with DecontX. Genome Biol 2020; 21:57.

6. Sainburg T, McInnes L, Gentner TQ. Parametric UMAP Embeddings for Representation and Semisupervised Learning. Neural Comput 2021; 33:2881-907.

7. Traag VA, Waltman L, van Eck NJ. From Louvain to Leiden: guaranteeing well-connected communities. Sci Rep 2019; 9:5233.

8. Franzen O, Gan LM, Bjorkegren JLM. PanglaoDB: a web server for exploration of mouse and human single-cell RNA sequencing data. Database (Oxford) 2019; baz046.

9. Andreatta M, Carmona SJ. UCell: Robust and scalable single-cell gene signature scoring. Comput Struct Biotechnol J 2021; 19:3796-8.
