# Supplementary material for: Bifidobacteria infantis and human milk oligosaccharides have independent and synergistic effects on immune response and amino acid metabolism in germ-free mouse models
Source: mSystems. 2026 Jun 15;11(7):e00392-26. doi: 10.1128/msystems.00392-26 (PMC13386997; doi:10.1128/msystems.00392-26)
Supplement: Legends — Supplemental figure legends. [file msystems.00392-26-s0008.pdf]

Figure S1. Confirmation of germ-free status of treatment groups. Representative images of microbial cultures taken during the trial to validate germ-free status. A mixture of feed, bedding, and water collected on days 7 and 14 was cultured in thioglycolate and tryptic soy broth as well as blood and Sabouraud agar plates. Treatment groups: control – orally gavaged with PBS for 14 d; human milk oligosaccharides group (HMO) - orally gavaged with pooled HMO for 14 d; BI group - orally gavaged with *Bifidobacterium longum subsp. infantis* ATCC 15697 on days 1, 4, and 9 of the 14 d experimental period; BI+HMO group - orally gavaged with HMO for all 14 d and BI on days 1, 4, and 9 of the 14 d experimental period.

Figure S2. Molecular validation of germ-free status in the treatment groups. (A) Electrophoresis of PCR products amplified from colonies grown on Sabouraud agar from the BI+HMO group using 16S and 18S primers. (B) The amplification products after using specific fungal primers (ITS2) on DNA extracted from the Sabouraud agar colonies and thioglycolate broth from the BI+HMO group and on day 14 fecal DNA from treatment groups. (C) Species-specific PCR amplification products using BI primers on DNA from Sabouraud agar colonies and thioglycolate broth from the BI+HMO group. (D) PCR amplification products using 16S specific primers on day 14 fecal DNA from treatment groups. Pos ctrl – Positive control; Pos 18S ctrl - Positive 18S control; Pos 16S ctrl - Positive 16S control; Sab col-BI+HMO - Sabouraud agar colonies from the BI +HMO group; TGB -BI+HMO – Thioglycolate broth from the BI+HMO group; Rep - Replicate; Ctrl – pooled DNA within the control group; HMO – Pooled DNA within the HMO group; BI - Pooled DNA within the BI group; BI+HMO - Pooled DNA within the BI+HMO group.

Figure S3. Effect of BI and HMO supplementation on body weight, organ weight and gut tissue morphology of germ-free mice collected on day 14. (A) body weight, (B) brain weight, (C) kidney weight, (D) duodenum crypt depth, (E) duodenum villi height, (F) jejunum villi height, (G) jejunum crypt depth, (H) colon crypt depth (I) cecum crypt depth. Treatment groups: control – orally gavaged with PBS for 14 d; human milk oligosaccharides group (HMO) - orally gavaged with pooled HMO for 14 d; BI group - orally gavaged with *Bifidobacterium longum subsp. infantis* ATCC 15697 on days 1, 4, and 9 of the 14 d experimental period; BI+HMO group - orally gavaged with HMO for all 14 d and BI on days 1, 4, and 9 of the 14 d experimental period. Statistical significance was calculated using two-way ANOVA with Tukey's multiple comparison tests in GraphPad Prism version 10.5.0. Means ± SEM are plotted.

Figure S4. Effect of BI and HMO supplementation on colonocyte populations in germ-free mice as assessed by single-cell RNA seq. (A) UMAP of 10 colonocyte subtype populations and (B) dot plot depicting colonocyte subtype-specific marker genes. (C) Relative distribution of colonocyte subtype populations. Statistical significance was calculated using two-way ANOVA with Tukey's multiple comparison tests in GraphPad Prism version 10.5.0. Means ± SEM are plotted (**** $P \leq 0.0001$).

Figure S5. Effect of BI and HMO supplementation on non-epithelial cell populations in germ-free mice as assessed by single-cell RNA seq. (A) UMAP of 13 non-epithelial cell populations and (B) dot plot depicting cell type specific marker genes. (C) Relative distribution of non-epithelial cell type populations. Statistical significance was calculated using two-way ANOVA with Tukey's multiple comparison tests in GraphPad Prism version 10.5.0. Means ± SEM are plotted (**** $P \leq 0.0001$).

Figure S6. Effect of BI and HMO supplementation on T cell, mast cell, and innate lymphoid cell (ILC) populations in germ-free mice as assessed by single-cell RNA seq. (A) UMAP of 10 T cell, mast cell, and ILC subtype populations and (B) dot plot depicting T cell, mast cell, and ILC subtype specific marker genes.
