# Supplementary material for: Bifidobacteria infantis and human milk oligosaccharides have independent and synergistic effects on immune response and amino acid metabolism in germ-free mouse models
Source: mSystems. 2026 Jun 15;11(7):e00392-26. doi: 10.1128/msystems.00392-26 (PMC13386997; doi:10.1128/msystems.00392-26)
Supplement: Supplemental Tables — Tables S1 to S8. [file msystems.00392-26-s0009.docx]

| **Antibody** | **Company** | **Catalog #** | **Clone** |
| --- | --- | --- | --- |
| AF488 ant-mouse GATA3 | BioLegend | 653808 | 16E10A23 |
| PerCP/Cy5.5 anti-mouse TCR b | BioLegend | 109228 | H57-597 |
| APC ROR gamma (t) | Invitrogen | 17-6981-82 | B2D |
| APC/Cy7 anti-mouse CD90.2 | BioLegend | 105328 | 30-H12 |
| efluor450 Rat anti-mouse FOXP3 | Invitrogen | 48-5773-82 | FJK-16s |
| BV711 anti-mouse CD3 | BioLegend | 100241 | 17A2 |
| BV 605 anti-mouse CD44 | BioLegend | 103047 | IM7 |
| BV786 Rat anti-mouse CD4 | BD Biosciences | 563727 | RM4-5 |
| PE anti-mouse anti-T-bet | BioLegend | 644810 | 4B10 |
| PE-CF594 Hamster anti-mouse gd TCR | BD Biosciences | 563532 | GL3 |
| PE-Cy7 Rat anti-mouse CD8b | Invitrogen | 25-0083-82 | H35-17.2 |
| FITC anti-mouse CD19 | BioLegend | 152404 | 1D3 |
| PerCP anti-mouse CD45.2 | BioLegend | 109826 | 104 |
| APC Hamster Anti-Mouse CD11c | BD Biosciences | 550261 | HL3 |
| BV 421 anti-mouse Ly6C | BioLegend | 128032 | HK1.4 |
| BV 605 anti-mouse CD64 | BioLegend | 139323 | X54-5/7.1 |
| BV 785 anti-mouse I-A/I-E, MHC-II | BioLegend | 107645 | M5/114.15.2 |
| PE-CF594 Rat anti-mouse Ly-6G (1A8) | BD Biosciences | 562710 | 1A8 |
| PE-Cy7 anti-mouse/human CD11b | BioLegend | 101216 | M1/70 |
| PE anti-mouse CD138 | BioLegend | 142504 | 281-2 |

**Table S1.** Antibodies used in quantification of mesenteric lymph node and spleen immune cell composition by flow cytometry assay.

| **Gene locus** | **Primer** | **Sequence** | **Cycling parameters** |
| --- | --- | --- | --- |
| BLON0915 | Blon0915_fw | CGTATTGGCTTTGTACGCATTT | Number of cycles - 40; Denaturation - 3 min, 95 °C;  Annealing-extension - 15 sec, 60 °C |
|  | Blon0915_rev | ATCGTGCCGGTGAGATTTAC |  |
|  | Blon0915_probe | FAM–CCAGTATGG–ZEN-CTGGTAAAGTTCACTGCA–3IABkFQ |  |

**Table S2.** qPCR primers, probes and conditions used to quantify *Bifidobacteria infantis*.

**Table S3.** Confirmation of germ-free status of treatment groups. A mixture of feed, bedding, and water collected on days 7 and 14 (n = 1-2 cages/group; 5 mice/cage) of the trial period was cultured in thioglycolate and tryptic soy broth as well as blood and Sabouraud agar plates. Treatment groups: control – orally gavaged with PBS for 14 d; human milk oligosaccharides group (HMO) - orally gavaged with pooled HMO for 14 d; BI group - orally gavaged with *Bifidobacterium longum subsp. infantis* ATCC 15697 on days 1, 4, and 9 of the 14 d experimental period; BI+HMO group - orally gavaged with HMO for all 14 d and BI on days 1, 4, and 9 of the 14 d experimental period. X = growth not seen; ✓ = growth seen.

|  | **Pre-trial** | | | | **Post-week 1 (Day 7)** | | | | **Post-week 2 (Day 14)** | | | |
| --- | --- | --- | --- | --- | --- | --- | --- | --- | --- | --- | --- | --- |
| Groups | Blood Agar | SABDEX Agar | Thioglycolate broth | Tryptic soy broth | Blood Agar | SABDEX Agar | Thioglycolate broth | Tryptic soy broth | Blood Agar | SABDEX Agar | Thioglycolate broth | Tryptic soy broth |
| **Control** | x | x | x | x | x | x | x | x | x | x | x | x |
| **HMO** | x | x | x | x | x | x | x | x | x | x | x | x |
| **HMO+BI** | x | x | x | x | x | x | ✓ | ✓ | ✓ | ✓ | ✓ | ✓ |
| **BI** | x | x | x | x | x | x | ✓ | x | x | x | ✓ | x |

**Table S4**. Average *C*_t_ values from quantitative polymerase chain reaction of BI in DNA isolated from the stool of germ-free mice (d 14; n = 10-14 mice/group) gavaged with BI and HMO. Treatment groups: human milk oligosaccharides group (HMO) - orally gavaged with pooled HMO for 14 d; BI group - orally gavaged with *Bifidobacterium longum subsp. infantis* ATCC 15697 on days 1, 4, and 9 of the 14 d experimental period; BI+HMO group - orally gavaged with HMO for all 14 d and BI on days 1, 4, and 9 of the 14 d experimental period. Control group – orally gavaged with PBS for 14 d.

| **Group** | **Average** *C*_t_ **value** |
| --- | --- |
| Control | 40 |
| HMO | 40 |
| BI | 15.8 |
| BI+HMO | 15.9 |

**Table S5.** Primer sequences used for molecular validation of germ-free status.

| **Target gene** | **Primer name** | **Sequence** | **Expected size** |
| --- | --- | --- | --- |
| Bacteria 16S | Fba/Cdf | TGACGACAACCATGCACC  GAATTGACGGGGGCCCGCACAAG | ~150 |
| 18S | 545 F /545 R | F: GAG GCC CTTG TAA TTG GAA TGA G  R: CGC TAT TGG AGG TGG AAT TAC C | ~104 |
| Fungal specific (ITS2) | ITS3-2024F / ITS4-2409R | GCATCGATGAAGAACGCAGC  TCCTCCGCTTATTGATATGC | ~200-400 |

**Table S6.** Number of epithelial and non-epithelial cells sequenced in germ-free mouse colon tissue per treatment, generated by scRNA-seq.

| **Treatment** | **Total number**  **of cells** | **Number of epithelial cells** | **Number of non-epithelial cells** |
| --- | --- | --- | --- |
| Control (n = 4) | 37,243 | 24,892 | 12,351 |
| HMO (n = 3) | 22,609 | 14,132 | 8,477 |
| BI (n = 4) | 33,303 | 19,413 | 13,890 |
| BI +HMO (n = 4) | 28,737 | 19,094 | 9,643 |
| **Total** | 121,892 | 77,531 (63.6%) | 44,361 (36.4%) |

HMO - human milk oligosaccharides; orally gavaged with pooled HMO for 14 d.

BI - orally gavaged with *Bifidobacterium longum* subsp. *infantis* ATCC 15697 on days 1, 4, and 9 of the 14 d experimental period.

BI+HMO group - orally gavaged with HMOs for all 14 d and BI on days 1, 4, and 9 of the 14 d experimental period.

Control group - orally gavaged with PBS for 14 d.

**Table S7**. Serum metabolites that are differentially regulated in BI-gavaged mice (BI and BI+HMO groups) compared with those not receiving BI (HMO and control groups) (n = 10-14 mice/group). Values represent the normalized abundance levels and may contain negative values due to normalization (median and pareto scaling). All data processing and normalization were performed using MetaboAnalyst 6.0 software with default parameters unless otherwise described. Treatment groups: HMO (human milk oligosaccharides) group - orally gavaged with pooled 2′-fucosyllactose, lacto-N-tetraose and 3′-sialyllactose at 15 mg/d (5 mg/HMO) for 14 d; BI group - orally gavaged with *Bifidobacterium longum subsp. infantis* ATCC 15697 (1x10^9 CFU/d) on days 1, 4, and 9 of the 14 d experimental period; BI+HMO group - orally gavaged with HMO for all 14 d and BI on days 1, 4, and 9 of the 14 d experimental period; Control group – orally gavaged with PBS for 14 d. Adjusted P-values were calculated using two-way ANOVA with Tukey’s multiple comparison tests in R studio version 4.4.1. Mean values in the same row with different letters differ (P < 0.05). HMD - Human Metabolome Database.

| **Metabolites** | | **Class** | | **HMD IDs** | | **BI** | | | | **No BI** | | | |  | | **Adjusted P value** | | | | | |
| --- | --- | --- | --- | --- | --- | --- | --- | --- | --- | --- | --- | --- | --- | --- | --- | --- | --- | --- | --- | --- | --- |
|  | |  | |  | | HMO | | No HMO | | HMO | | No HMO | |  | | HMO | | BI | | BI × HMO | |
| **Up regulated** | |  | |  | |  | |  | |  | |  | |  | |  | |  | |  |  |
| 1,3-Diaminopropane | | Organic acids | | HMDB0000002 | | 0.092 ^b^ | | 1.144 ^c^ | | -0.543 ^ab^ | | -0.693 ^a^ | |  | | 0.457 | | <0.001 | | 0.561 |  |
| 2-Hydroxy-2-methylbutyric acid | | Organic acids | | HMDB0001987 | | 0.198 ^b^ | | 1.325 ^c^ | | -0.845 ^a^ | | -0.678 ^a^ | |  | | 0.004 | | <0.001 | | 0.052 |  |
| 2-Hydroxy-3-methylvaleric acid | | Organic acids | | HMDB0000317 | | 0.275 ^ab^ | | 0.759 ^b^ | | -0.514 ^a^ | | -0.519 ^a^ | |  | | 0.005 | | <0.001 | | 0.077 |  |
| 2-Hydroxybutyric acid | | Organic acids | | HMDB0000008 | | 0.155 ^b^ | | 1.224 ^c^ | | -0.979 ^a^ | | -0.399 ^ab^ | |  | | 0.006 | | <0.001 | | 0.091 |  |
| 2-Hydroxyisobutyric acid | | Organic acids | | HMDB0000729 | | 0.225 ^b^ | | 1.100 ^c^ | | -0.638 ^a^ | | -0.687 ^a^ | |  | | 0.004 | | <0.001 | | 0.052 |  |
| 2-Hydroxyglutaric acid | | Organic acids | | HMDB0059655 | | -0.054 ^ab^ | | 0.830 ^b^ | | -0.305 ^a^ | | -0.470 ^a^ | |  | | 0.003 | | <0.001 | | 0.548 |  |
| 2-Hydroxyisovaleric acid | | Organic acids | | HMDB0000407 | | 0.216 ^ab^ | | 0.826 ^b^ | | -0.529 ^a^ | | -0.513 ^a^ | |  | | 0.004 | | <0.001 | | 0.134 |  |
| 3-Aminoisobutyric acid | | Organic acids | | HMDB0003911 | | 0.269 ^b^ | | 1.172 ^c^ | | -0.744 ^a^ | | -0.697 ^a^ | |  | | 0.004 | | <0.001 | | 0.118 |  |
| 3-Indoleacetic acid | | Organic acids | | HMDB0000197 | | 0.140 ^a^ | | 0.967 ^b^ | | -0.505 ^a^ | | -0.603 ^a^ | |  | | 0.007 | | <0.001 | | 0.052 |  |
| 3-Hydroxyisobutyric acid | | Organic acids | | HMDB0000336 | | 0.356 ^bc^ | | 0.801 ^c^ | | -0.819 ^a^ | | -0.338 ^ab^ | |  | | 0.008 | | <0.001 | | 0.061 |  |
| 3-Hydroxyisovaleric acid | | Organic acids | | HMDB0000754 | | 0.183 ^b^ | | 1.195 ^c^ | | -0.825 ^a^ | | -0.554 ^a^ | |  | | 0.012 | | <0.001 | | 0.211 |  |
| 3-Hydroxybutyric acid | | Organic acids | | HMDB0000011 | | -0.112 ^a^ | | 1.027 ^b^ | | -0.672 ^a^ | | -0.244 ^a^ | |  | | 0.006 | | <0.001 | | 0.357 |  |
| 4-Hydroxyphenylacetic acid | | Organic acids | | HMDB0000020 | | 0.187 ^ab^ | | 0.534 ^b^ | | -0.136 ^ab^ | | -0.584 ^a^ | |  | | 0.012 | | <0.001 | | 0.061 |  |
| 3-Methyladipic acid | | Organic acids | | HMDB0000555 | | -0.281 ^a^ | | 0.781 ^b^ | | -0.180 ^a^ | | -0.320 ^a^ | |  | | 0.039 | | <0.001 | | 0.114 |  |
| 4-Hydroxyphenylpyruvic acid | | Organic acids | | HMDB0000707 | | 0.200 ^ab^ | | 0.781 ^b^ | | -0.401 ^a^ | | -0.580 ^a^ | |  | | 0.011 | | <0.001 | | 0.052 |  |
| 5-Hydroxyindoleacetic acid | | Organic acids | | HMDB0000763 | | -0.034 ^a^ | | 0.878 ^b^ | | -0.373 ^a^ | | -0.471 ^a^ | |  | | 0.009 | | <0.001 | | 0.052 |  |
| 5-Oxoproline | | Organic acids | | HMDB0000267 | | -0.388 ^a^ | | 0.913 ^b^ | | -0.176 ^a^ | | -0.350 ^a^ | |  | | 0.004 | | <0.001 | | 0.052 |  |
| Adenine | | Organic acids | | HMDB0000034 | | 0.137 ^b^ | | 1.099 ^c^ | | -0.508 ^ab^ | | -0.729 ^a^ | |  | | 0.008 | | <0.001 | | 0.056 |  |
| 7-Methylguanine | | Organic acids | | HMDB0000897 | | -0.054 ^a^ | | 0.947 ^b^ | | -0.465 ^a^ | | -0.429 ^a^ | |  | | 0.006 | | <0.001 | | 0.061 |  |
| Adenosine | | Nucleobases | | HMDB0000050 | | 0.038 ^ab^ | | 0.787 ^b^ | | -0.117 ^a^ | | -0.708 ^a^ | |  | | 0.004 | | <0.001 | | 0.623 |  |
| Alanine | | Amino acid | | HMDB0000161 | | 0.396 ^ab^ | | 0.498 ^b^ | | -0.426 ^ab^ | | -0.468 ^a^ | |  | | 0.014 | | <0.001 | | 0.054 |  |
| Allantoin | | Alkaloids | | HMDB0000462 | | 0.283 ^b^ | | 1.223 ^c^ | | -0.813 ^a^ | | -0.693 ^a^ | |  | | 0.003 | | <0.001 | | 0.586 |  |
| alpha-Aminoadipic acid | | Amino acid - related | | HMDB0000510 | | 0.317 ^b^ | | 0.973 ^b^ | | -0.599 ^a^ | | -0.690 ^a^ | |  | | 0.003 | | <0.001 | | 0.111 |  |
| alpha-Ketoglutaric acid | | Organic acids | | HMDB0000208 | | 0.112 ^ab^ | | 0.879 ^b^ | | -0.560 ^a^ | | -0.431 ^a^ | |  | | 0.006 | | <0.001 | | 0.252 |  |
| alpha-Ketoisovaleric acid | | Organic acids | | HMDB0000019 | | 0.079 ^b^ | | 1.338 ^c^ | | -0.860 ^a^ | | -0.557 ^a^ | |  | | 0.011 | | <0.001 | | 0.057 |  |
| Arginine | | Amino acid | | HMDB0000517 | | 0.079 ^a^ | | 0.351 ^a^ | | 0.155 ^a^ | | -0.585 ^a^ | |  | | 0.012 | | <0.001 | | 0.061 |  |
| Argininic acid | | Organic acids | | HMDB0003148 | | 0.045 ^a^ | | -0.442 ^a^ | | 0.373 ^a^ | | 0.024 ^a^ | |  | | 0.007 | | <0.001 | | 0.053 |  |
| Asparagine | | Amino acid | | HMDB0000168 | | 0.437 ^bc^ | | 0.777 ^c^ | | -0.859 ^a^ | | -0.355 ^ab^ | |  | | 0.013 | | <0.001 | | 0.892 |  |
| Aspartic acid | | Amino acid | | HMDB0000191 | | -0.060 ^a^ | | 0.299 ^a^ | | -0.236 ^a^ | | -0.003 ^a^ | |  | | 0.066 | | <0.001 | | 0.226 |  |
| beta-Alanine | | Amino acid - related | | HMDB0000056 | | -0.044 ^a^ | | 0.574 ^a^ | | -0.274 ^a^ | | -0.256 ^a^ | |  | | 0.015 | | <0.001 | | 0.130 |  |
| Betaine | | Amino acid - related | | HMDB0000043 | | 0.214 ^bc^ | | 0.950 ^c^ | | -0.533 ^ab^ | | -0.631 ^a^ | |  | | 0.007 | | <0.001 | | 0.196 |  |
| Butyric acid | | Short chain fatty acid | | HMDB0000039 | | 0.150 ^b^ | | 1.253 ^c^ | | -0.724 ^a^ | | -0.679 ^a^ | |  | | 0.116 | | <0.001 | | 0.191 |  |
| C12 | | Acylcarnitines | | HMDB0002250 | | 0.224 ^b^ | | 1.069 ^c^ | | -0.702 ^a^ | | -0.592 ^a^ | |  | | 0.003 | | <0.001 | | 0.208 |  |
| C12:1 | | Acylcarnitines | | HMDB0013326 | | 0.177 ^b^ | | 1.007 ^c^ | | -0.862 ^a^ | | -0.322 ^ab^ | |  | | 0.016 | | <0.001 | | 0.112 |  |
| C12DC | | Acylcarnitines | | HMDB0013327 | | -0.220 ^a^ | | 0.565 ^a^ | | -0.093 ^a^ | | -0.252 ^a^ | |  | | 0.009 | | <0.001 | | 0.386 |  |
| C14:1 | | Acylcarnitines | | HMDB0002014 | | -0.151 ^a^ | | 1.165 ^b^ | | -0.484 ^a^ | | -0.530 ^a^ | |  | | 0.028 | | <0.001 | | 0.348 |  |
| C14:2 | | Acylcarnitines | | HMDB0013331 | | 0.185 ^b^ | | 1.222 ^c^ | | -0.779 ^a^ | | -0.628 ^a^ | |  | | 0.017 | | <0.001 | | 0.924 |  |
| C14:2OH | | Acylcarnitines | | HMDB0013332 | | 0.350 ^b^ | | 1.084 ^c^ | | -0.618 ^a^ | | -0.816 ^a^ | |  | | 0.027 | | <0.001 | | 0.390 |  |
| C18:1 | | Acylcarnitines | | HMDB0006464 | | 0.148 ^b^ | | 1.235 ^c^ | | -0.907 ^a^ | | -0.476 ^a^ | |  | | 0.018 | | <0.001 | | 0.054 |  |
| C18 | | Acylcarnitines | | HMDB0000848 | | 0.406 ^b^ | | 1.062 ^c^ | | -0.940 ^a^ | | -0.528 ^a^ | |  | | 0.023 | | <0.001 | | 0.094 |  |
| C18:2 | | Acylcarnitines | | HMDB0006469 | | -0.026 ^a^ | | 1.094 ^b^ | | -0.602 ^a^ | | -0.466 ^a^ | |  | | 0.029 | | <0.001 | | 0.218 |  |
| C16 | | Acylcarnitines | | HMDB0000222 | | 0.380 ^b^ | | 1.209 ^c^ | | -1.004 ^a^ | | -0.585 ^a^ | |  | | 0.003 | | <0.001 | | 0.536 |  |
| C3 | | Acylcarnitines | | HMDB0000824 | | 0.404 ^b^ | | 0.882 ^b^ | | -0.761 ^a^ | | -0.526 ^a^ | |  | | 0.005 | | <0.001 | | 0.078 |  |
| C4 | | Acylcarnitines | | HMDB0002013 | | 0.398 ^b^ | | 0.825 ^b^ | | -0.636 ^a^ | | -0.586 ^a^ | |  | | 0.025 | | <0.001 | | 0.112 |  |
| C4:1 | | Acylcarnitines | | HMDB0013126 | | 0.415 ^b^ | | 0.863 ^b^ | | -0.836 ^a^ | | -0.442 ^a^ | |  | | 0.065 | | <0.001 | | 0.265 |  |
| C4OH | | Acylcarnitines | | HMDB0013127 | | 0.114 ^b^ | | 1.315 ^c^ | | -0.839 ^a^ | | -0.589 ^a^ | |  | | 0.003 | | <0.001 | | 0.056 |  |
| C5 | | Acylcarnitines | | HMDB0013128 | | 0.296 ^b^ | | 1.037 ^c^ | | -0.671 ^a^ | | -0.662 ^a^ | |  | | 0.025 | | <0.001 | | 0.063 |  |
| C5:1DC | | Acylcarnitines | | HMDB0013129 | | 0.378 ^bc^ | | 0.826 ^c^ | | -0.866 ^a^ | | -0.339 ^ab^ | |  | | 0.036 | | <0.001 | | 0.211 |  |
| C5OH | | Acylcarnitines | | HMDB0013132 | | 0.330 ^bc^ | | 0.743 ^c^ | | -0.760 ^a^ | | -0.313 ^ab^ | |  | | 0.032 | | <0.001 | | 0.061 |  |
| C6 | | Acylcarnitines | | HMDB0000756 | | 0.330 ^b^ | | 0.661 ^b^ | | -0.800 ^a^ | | -0.191 ^ab^ | |  | | 0.012 | | <0.001 | | 0.203 |  |
| C6:1 | | Acylcarnitines | | HMDB0013161 | | 0.232 ^b^ | | 1.063 ^c^ | | -0.777 ^a^ | | -0.519 ^a^ | |  | | 0.069 | | <0.001 | | 0.052 |  |
| C8 | | Acylcarnitines | | HMDB0000791 | | 0.355 ^bc^ | | 0.904 ^c^ | | -0.876 ^a^ | | -0.383 ^ab^ | |  | | 0.003 | | <0.001 | | 0.292 |  |
| C9 | | Acylcarnitines | | HMDB0006320 | | 0.397 ^b^ | | 0.891 ^b^ | | -0.672 ^a^ | | -0.616 ^a^ | |  | | 0.003 | | <0.001 | | 0.427 |  |
| Caprylic acid | | Organic acids | | HMDB0000482 | | 0.397 ^a^ | | 0.259 ^a^ | | -0.460 ^a^ | | -0.196 ^a^ | |  | | 0.015 | | <0.001 | | 0.128 |  |
| Carnosine | | Amino acid - related | | HMDB0000033 | | -0.126 ^a^ | | 0.950 ^b^ | | -0.458 ^a^ | | -0.366 ^a^ | |  | | 0.004 | | <0.001 | | 0.061 |  |
| CE(18:2) | | Cholesterol esters | | HMDB05192 | | 0.479 ^a^ | | -0.064 ^a^ | | -0.013 ^a^ | | -0.402 ^a^ | |  | | 0.023 | | <0.001 | | 0.352 |  |
| CE(18:3) | | Cholesterol esters | | HMDB10369 | | 0.411 ^a^ | | -0.092 ^a^ | | 0.011 ^a^ | | -0.330 ^a^ | |  | | 0.069 | | <0.001 | | 0.221 |  |
| CE(20:4) | | Acylcarnitines | | HMDB06726 | | 0.652 ^b^ | | 0.587 ^b^ | | -0.622 ^a^ | | -0.617 ^a^ | |  | | 0.018 | | <0.001 | | 0.061 |  |
| CE(20:3) | | Acylcarnitines | | HMDB06736 | | 0.669 ^b^ | | 0.116 ^ab^ | | -0.348 ^a^ | | -0.437 ^a^ | |  | | 0.018 | | <0.001 | | 0.246 |  |
| CE(20:5) | | Acylcarnitines | | HMDB06731 | | 0.197 ^a^ | | 0.263 ^a^ | | -0.035 ^a^ | | -0.425 ^a^ | |  | | 0.047 | | <0.001 | | 0.159 |  |
| CE(22:5) | | Acylcarnitines | | HMDB10374 | | 0.611 ^a^ | | 0.064 ^a^ | | -0.337 ^a^ | | -0.339 ^a^ | |  | | 0.004 | | <0.001 | | 0.289 |  |
| Cer(d18:1/16:0) | | Ceramides | | HMDB00790 | | -0.050 ^a^ | | 1.004 ^b^ | | -0.344 ^a^ | | -0.610 ^a^ | |  | | 0.033 | | <0.001 | | 0.823 |  |
| Cer(d18:1/22:0) | | Ceramides | | HMDB04952 | | 0.332 ^ab^ | | 0.675 ^b^ | | -0.568 ^a^ | | -0.439 ^a^ | |  | | 0.051 | | <0.001 | | 0.059 |  |
| Cer(d18:1/24:0) | | Ceramides | | HMDB04956 | | 0.122 ^b^ | | 1.087 ^c^ | | -0.556 ^ab^ | | -0.654 ^a^ | |  | | 0.022 | | <0.001 | | 0.083 |  |
| Cer(d18:1/24:1) | | Ceramides | | HMDB04953 | | 0.270 ^bc^ | | 0.894 ^c^ | | -0.555 ^ab^ | | -0.610 ^a^ | |  | | 0.077 | | <0.001 | | 0.319 |  |
| Cer(d18:2/24:0) | | Ceramides | | HMDB0341520 | | 0.065 ^a^ | | 0.127 ^a^ | | 0.194 ^a^ | | -0.386 ^a^ | |  | | 0.076 | | <0.001 | | 0.512 |  |
| Cer(d18:2/24:1) | | Ceramides | | HMDB0240680 | | 0.102 ^ab^ | | 0.802 ^b^ | | -0.427 ^a^ | | -0.477 ^a^ | |  | | 0.900 | | <0.001 | | 0.106 |  |
| Choline | | Vitamins | | HMDB0000097 | | 0.056 ^b^ | | 1.237 ^c^ | | -0.806 ^a^ | | -0.488 ^ab^ | |  | | 0.141 | | <0.001 | | 0.579 |  |
| Citrulline | | Amino acid - related | | HMDB0000904 | | 0.244 ^bc^ | | 0.890 ^c^ | | -0.487 ^ab^ | | -0.647 ^a^ | |  | | 0.019 | | <0.001 | | 0.208 |  |
| Creatine | | Biogenic Amines | | HMDB0000064 | | 0.174 ^b^ | | 1.078 ^c^ | | -0.713 ^a^ | | -0.540 ^ab^ | |  | | 0.030 | | <0.001 | | 0.819 |  |
| Creatinine | | Biogenic Amines | | HMDB0000562 | | -0.140 ^a^ | | 0.837 ^b^ | | -0.347 ^a^ | | -0.351 ^a^ | |  | | 0.111 | | <0.001 | | 0.182 |  |
| Deoxycytidine | | Nucleoside | | HMDB0000014 | | 0.771 ^c^ | | 0.834 ^c^ | | -1.206 ^a^ | | -0.399 ^b^ | |  | | 0.026 | | <0.001 | | 0.077 |  |
| Deoxyuridine | | Nucleoside | | HMDB0000012 | | 0.446 ^b^ | | 1.060 ^b^ | | -0.885 ^a^ | | -0.620 ^a^ | |  | | 0.026 | | <0.001 | | 0.422 |  |
| DG(14:0_20:0) | | Diglycerides | | HMDB07020 | | 0.669 ^b^ | | -0.646 ^a^ | | -0.117 ^ab^ | | 0.093 ^ab^ | |  | | 0.003 | | <0.001 | | 0.340 |  |
| Dimethylamine | | Amino acid - related | | HMDB0000087 | | -0.119 ^ab^ | | 0.657 ^b^ | | 0.100 ^ab^ | | -0.638 ^a^ | |  | | 0.033 | | <0.001 | | 0.061 |  |
| Ethanolamine | | Amino acid - related | | HMDB0000149 | | 0.066 ^a^ | | 1.017 ^b^ | | -0.655 ^a^ | | -0.427 ^a^ | |  | | 0.048 | | <0.001 | | 0.061 |  |
| Dimethylglycine | | Amino acid - related | | HMDB0000092 | | 0.216 ^c^ | | 1.224 ^d^ | | -1.034 ^a^ | | -0.406 ^b^ | |  | | 0.022 | | <0.001 | | 0.111 |  |
| Ethylmalonic acid | | Organic acids | | HMDB0000622 | | -0.113 ^a^ | | 1.265 ^b^ | | -0.737 ^a^ | | -0.415 ^a^ | |  | | 0.021 | | <0.001 | | 0.053 |  |
| gamma-Aminobutyric acid | | Amino acid - related | | HMDB0000112 | | 0.226 ^c^ | | 1.220 ^d^ | | -1.075 ^a^ | | -0.371 ^b^ | |  | | 0.076 | | <0.001 | | 0.063 |  |
| Glucose | | Sugars | | HMDB0000122 | | 0.382 ^b^ | | 1.136 ^c^ | | -0.814 ^a^ | | -0.704 ^a^ | |  | | 0.156 | | <0.001 | | 0.637 |  |
| Glutamic acid | | Amino acid | | HMDB0000148 | | -0.151 ^ab^ | | 0.686 ^b^ | | -0.241 ^ab^ | | -0.293 ^a^ | |  | | 0.033 | | <0.001 | | 0.218 |  |
| Glutaric acid | | Organic acids | | HMDB0000661 | | 0.142 ^b^ | | 1.080 ^c^ | | -0.702 ^a^ | | -0.519 ^ab^ | |  | | 0.038 | | <0.001 | | 0.052 |  |
| Guanidinopropionic acid | | Organic acids | | HMDB0013222 | | 0.108 ^ab^ | | 0.936 ^b^ | | -0.667 ^a^ | | -0.376 ^a^ | |  | | 0.007 | | <0.001 | | 0.927 |  |
| Hex2Cer(d18:1/14:0) | | Glycosylceramides | | HMDB0341522 | | 0.001 ^a^ | | 0.898 ^b^ | | -0.486 ^a^ | | -0.414 ^a^ | |  | | 0.213 | | <0.001 | | 0.061 |  |
| Hex2Cer(d18:1/16:0) | | Glycosylceramides | | HMDB0006750 | | 0.281 ^b^ | | 1.156 ^c^ | | -0.821 ^a^ | | -0.616 ^a^ | |  | | 0.004 | | <0.001 | | 0.953 |  |
| Hex2Cer(d18:1/18:0) | | Glycosylceramides | | HMDB0011591 | | 0.342 ^bc^ | | 0.835 ^c^ | | -0.731 ^a^ | | -0.446 ^ab^ | |  | | 0.003 | | <0.001 | | 0.723 |  |
| Hex2Cer(d18:1/20:0) | | Glycosylceramides | | HMDB0011593 | | 0.285 ^a^ | | 0.176 ^a^ | | -0.498 ^a^ | | 0.038 ^a^ | |  | | 0.168 | | <0.001 | | 0.104 |  |
| Hex2Cer(d18:1/22:0) | | Glycosylceramides | | HMDB0011594 | | 0.358 ^b^ | | 0.965 ^b^ | | -0.608 ^a^ | | -0.715 ^a^ | |  | | 0.023 | | <0.001 | | 0.111 |  |
| Hex2Cer(d18:1/24:1) | | Glycosylceramides | | HMDB04872 | | 0.345 ^b^ | | 1.121 ^c^ | | -0.776 ^a^ | | -0.690 ^a^ | |  | | 0.016 | | <0.001 | | 0.578 |  |
| Hex3Cer(d18:1/16:0) | | Glycosylceramides | | HMDB0004879 | | 0.438 ^bc^ | | 0.879 ^c^ | | -0.259 ^b^ | | -1.057 ^a^ | |  | | 0.016 | | <0.001 | | 0.061 |  |
| Hex3Cer(d18:1/24:1) | | Glycosylceramides | | HMDB0004883 | | 0.512 ^a^ | | 0.356 ^a^ | | -0.426 ^a^ | | -0.442 ^a^ | |  | | 0.277 | | <0.001 | | 0.114 |  |
| HexCer(d16:1/22:0) | | Glycosylceramides | | HMDB0341523 | | 0.311 ^a^ | | -0.143 ^a^ | | -0.378 ^a^ | | 0.210 ^a^ | |  | | 0.019 | | <0.001 | | 0.196 |  |
| HexCer(d18:1/16:0) | | Glycosylceramides | | HMDB0004971 | | 0.305 ^b^ | | 1.193 ^c^ | | -0.864 ^a^ | | -0.633 ^a^ | |  | | 0.017 | | <0.001 | | 0.192 |  |
| HexCer(d18:1/18:0) | | Glycosylceramides | | HMDB0004972 | | 0.209 ^b^ | | 1.157 ^c^ | | -0.849 ^a^ | | -0.516 ^a^ | |  | | 0.004 | | <0.001 | | 0.091 |  |
| HexCer(d18:1/18:1) | | Glycosylceramides | | HMDB0004970 | | 0.389 ^bc^ | | 0.898 ^c^ | | -0.939 ^a^ | | -0.348 ^ab^ | |  | | 0.025 | | <0.001 | | 0.711 |  |
| HexCer(d18:1/20:0) | | Glycosylceramides | | HMDB0004973 | | 0.333 ^b^ | | 0.884 ^b^ | | -0.638 ^a^ | | -0.579 ^a^ | |  | | 0.018 | | <0.001 | | 0.689 |  |
| HexCer(d18:1/23:0) | | Glycosylceramides | | HMDB0341524 | | 0.236 ^b^ | | 1.185 ^c^ | | -0.756 ^a^ | | -0.665 ^a^ | |  | | 0.022 | | <0.001 | | 0.051 |  |
| HexCer(d18:1/22:0) | | Glycosylceramides | | HMDB0004974 | | 0.287 ^bc^ | | 0.822 ^c^ | | -0.693 ^a^ | | -0.416 ^ab^ | |  | | 0.004 | | <0.001 | | 0.124 |  |
| HexCer(d18:1/24:0) | | Glycosylceramides | | HMDB0004978 | | 0.322 ^b^ | | 1.175 ^c^ | | -0.797 ^a^ | | -0.699 ^a^ | |  | | 0.028 | | <0.001 | | 0.217 |  |
| HexCer(d18:1/24:1) | | Glycosylceramides | | HMDB0004975 | | 0.310 ^b^ | | 1.158 ^c^ | | -0.809 ^a^ | | -0.659 ^a^ | |  | | 0.025 | | <0.001 | | 0.998 |  |
| HexCer(d18:1/26:1) | | Glycosylceramides | | HMDB0004976 | | 0.404 ^bc^ | | 0.542 ^c^ | | -0.534 ^a^ | | -0.411 ^ab^ | |  | | 0.023 | | <0.001 | | 0.128 |  |
| HexCer(d18:2/16:0) | | Glycosylceramides | | HMDB0341525 | | 0.261 ^ab^ | | 0.684 ^b^ | | -0.552 ^a^ | | -0.393 ^a^ | |  | | 0.023 | | <0.001 | | 0.192 |  |
| HexCer(d18:2/18:0) | | Glycosylceramides | | HMDB0341526 | | 0.292 ^ab^ | | 0.559 ^b^ | | -0.497 ^a^ | | -0.354 ^ab^ | |  | | 0.046 | | <0.001 | | 0.103 |  |
| HexCer(d18:2/20:0) | | Glycosylceramides | | HMDB0341527 | | 0.469 ^b^ | | 0.582 ^b^ | | -0.831 ^a^ | | -0.220 ^ab^ | |  | | 0.009 | | <0.001 | | 0.148 |  |
| HexCer(d18:2/23:0) | | Glycosylceramides | | HMDB0341529 | | 0.212 ^bc^ | | 0.875 ^c^ | | -0.662 ^a^ | | -0.425 ^ab^ | |  | | 0.019 | | <0.001 | | 0.272 |  |
| Hippuric acid | | Organic acids | | HMDB0000714 | | -0.023 ^a^ | | 1.193 ^b^ | | -0.541 ^a^ | | -0.628 ^a^ | |  | | 0.188 | | <0.001 | | 0.221 |  |
| Homoarginine | | Amino acid-related | | HMDB0000670 | | 0.265 ^bc^ | | 0.903 ^c^ | | -0.634 ^a^ | | -0.534 ^ab^ | |  | | 0.041 | | <0.001 | | 0.621 |  |
| Indole | | Short chain fatty acid | | HMDB0000738 | | 0.292 ^b^ | | 1.158 ^c^ | | -0.804 ^a^ | | -0.646 ^a^ | |  | | 0.091 | | <0.001 | | 0.388 |  |
| Indole-3-propionic acid | | Indole derivatives | | HMDB0002302 | | 0.084 ^ab^ | | 0.803 ^b^ | | -0.144 ^a^ | | -0.743 ^a^ | |  | | 0.511 | | <0.001 | | 0.128 |  |
| Indoxyl sulfate | | Indole derivatives | | HMDB0000682 | | -0.307 ^a^ | | 1.077 ^b^ | | -0.347 ^a^ | | -0.424 ^a^ | |  | | 0.140 | | <0.001 | | 0.561 |  |
| Indolelactic acid | | Indole derivatives | | HMDB0000671 | | 0.795 ^b^ | | 1.013 ^b^ | | -0.909 ^a^ | | -0.898 ^a^ | |  | | 0.014 | | <0.001 | | 0.622 |  |
| Isoleucine | | Amino acids | | HMDB0000172 | | 0.242 ^b^ | | 1.089 ^c^ | | -0.854 ^a^ | | -0.478 ^a^ | |  | | 0.007 | | <0.001 | | 0.131 |  |
| Kynurenic acid | | Organic acids | | HMDB0000715 | | -0.328 ^ab^ | | 0.583 ^b^ | | 0.150 ^ab^ | | -0.404 ^a^ | |  | | 0.132 | | <0.001 | | 0.272 |  |
| Lactic acid | | Organic acids | | HMDB0000190 | | 0.117 ^ab^ | | 0.890 ^b^ | | -0.482 ^a^ | | -0.526 ^a^ | |  | | 0.099 | | <0.001 | | 0.061 |  |
| Leucine | | Amino acids | | HMDB0000687 | | 0.307 ^b^ | | 0.986 ^b^ | | -0.752 ^a^ | | -0.541 ^a^ | |  | | 0.033 | | <0.001 | | 0.710 |  |
| LysoPC a C14:0 | | Glycerophospholipids | | HMDB0010379 | | 0.386 ^b^ | | 1.139 ^c^ | | -0.856 ^a^ | | -0.669 ^a^ | |  | | 0.214 | | <0.001 | | 0.637 |  |
| Lysine | | Amino acids | | HMDB0000182 | | 0.352 ^b^ | | 1.028 ^b^ | | -0.788 ^a^ | | -0.592 ^a^ | |  | | 0.063 | | <0.001 | | 0.808 |  |
| LysoPC a C16:0 | | Phosphatidylcholines | | HMDB0010382 | | 0.443 ^b^ | | 1.132 ^c^ | | -0.795 ^a^ | | -0.780 ^a^ | |  | | 0.096 | | <0.001 | | 0.510 |  |
| LysoPC a C16:1 | | Glycerophospholipids | | HMDB0010383 | | 0.403 ^b^ | | 1.140 ^c^ | | -0.925 ^a^ | | -0.619 ^a^ | |  | | 0.101 | | <0.001 | | 0.651 |  |
| LysoPC a C17:0 | | Glycerophospholipids | | HMDB0012108 | | 0.355 ^b^ | | 1.124 ^c^ | | -0.746 ^a^ | | -0.733 ^a^ | |  | | 0.028 | | <0.001 | | 0.953 |  |
| LysoPC a C18:0 | | Glycerophospholipids | | HMDB0010384 | | 0.440 ^b^ | | 1.144 ^c^ | | -0.848 ^a^ | | -0.735 ^a^ | |  | | 0.110 | | <0.001 | | 0.425 |  |
| LysoPC a C18:2 | | Glycerophospholipids | | HMDB0010386 | | 0.417 ^b^ | | 1.129 ^c^ | | -0.846 ^a^ | | -0.699 ^a^ | |  | | 0.033 | | <0.001 | | 0.723 |  |
| LysoPC a C20:3 | | Glycerophospholipids | | HMDB0010393 | | 0.534 ^b^ | | 0.970 ^b^ | | -0.966 ^a^ | | -0.538 ^a^ | |  | | 0.092 | | <0.001 | | 0.386 |  |
| LysoPC a C20:4 | | Glycerophospholipids | | HMDB0010395 | | 0.389 ^c^ | | 1.207 ^d^ | | -1.079 ^a^ | | -0.517 ^b^ | |  | | 0.062 | | <0.001 | | 0.157 |  |
| LysoPC a C28:0 | | Glycerophospholipids | | HMDB0029206 | | 0.198 ^b^ | | 1.136 ^c^ | | -0.703 ^a^ | | -0.631 ^a^ | |  | | 0.021 | | <0.001 | | 0.061 |  |
| Maleic acid | | Organic acids | | HMDB0000176 | | -0.150 ^a^ | | 0.472 ^a^ | | 0.084 ^a^ | | -0.406 ^a^ | |  | | 0.024 | | <0.001 | | 0.819 |  |
| Malic acid | | Organic acids | | HMDB0000156 | | -0.233 ^a^ | | 0.781 ^b^ | | -0.188 ^a^ | | -0.360 ^a^ | |  | | 0.106 | | <0.001 | | 0.174 |  |
| Malonic acid | | Organic acids | | HMDB0000691 | | -0.218 ^a^ | | 1.256 ^b^ | | -0.614 ^a^ | | -0.424 ^a^ | |  | | 0.029 | | <0.001 | | 0.968 |  |
| Methionine | | Amino acids | | HMDB0000696 | | 0.471 ^b^ | | 0.526 ^b^ | | -0.450 ^a^ | | -0.547 ^a^ | |  | | 0.019 | | <0.001 | | 0.394 |  |
| Methionine sulfoxide | | Amino acid - related | | HMDB0002005 | | 0.254 ^a^ | | 0.264 ^a^ | | 0.099 ^a^ | | -0.618 ^a^ | |  | | 0.129 | | <0.001 | | 0.138 |  |
| Methylhistidine | | Amino acid-related | | HMDB0000479 | | 0.172 ^b^ | | 1.076 ^c^ | | -0.645 ^a^ | | -0.603 ^a^ | |  | | 0.435 | | <0.001 | | 0.565 |  |
| Methylmalonic acid | | Organic acids | | HMDB0000202 | | -0.045 ^a^ | | 1.218 ^b^ | | -0.615 ^a^ | | -0.558 ^a^ | |  | | 0.495 | | <0.001 | | 0.055 |  |
| N1-Acetyl-Lysine | | Amino acid - related | | HMDB0000446 | | -0.154 ^a^ | | 0.793 ^b^ | | -0.104 ^ab^ | | -0.534 ^a^ | |  | | 0.070 | | <0.001 | | 0.071 |  |
| N-Acetyl-Alanine | | Amino acid - related | | HMDB0000766 | | 0.072 ^a^ | | 0.957 ^b^ | | -0.528 ^a^ | | -0.502 ^a^ | |  | | 0.228 | | <0.001 | | 0.174 |  |
| N-Acetyl-Asparagine | | Amino acid - related | | HMDB0006028 | | 0.313 ^b^ | | 0.943 ^b^ | | -0.684 ^a^ | | -0.572 ^a^ | |  | | 0.028 | | <0.001 | | 0.422 |  |
| N-Acetyl-Aspartic acid | | Amino acid - related | | HMDB0000812 | | -0.051 ^a^ | | 0.473 ^a^ | | -0.435 ^a^ | | 0.013 ^a^ | |  | | 0.130 | | <0.001 | | 0.360 |  |
| N-Acetyl-Glutamic acid | | Amino acid - related | | HMDB0001138 | | 0.426 ^b^ | | 0.808 ^b^ | | -0.576 ^a^ | | -0.658 ^a^ | |  | | 0.063 | | <0.001 | | 0.510 |  |
| N-Acetyl-Glycine | | Amino acid - related | | HMDB0000532 | | 0.044 ^b^ | | 1.268 ^c^ | | -0.844 ^a^ | | -0.468 ^ab^ | |  | | 0.136 | | <0.001 | | 0.157 |  |
| N-Acetyl-Isoleucine | | Amino acid - related | | HMDB0061684 | | 0.153 ^b^ | | 1.042 ^c^ | | -0.758 ^a^ | | -0.436 ^ab^ | |  | | 0.057 | | <0.001 | | 0.357 |  |
| N-Acetyl-Leucine | | Amino acid - related | | HMDB0011756 | | 0.110 ^b^ | | 1.090 ^c^ | | -0.653 ^a^ | | -0.547 ^ab^ | |  | | 0.092 | | <0.001 | | 0.237 |  |
| N-Acetyl-Methionine | | Amino acid - related | | HMDB0011745 | | 0.089 ^ab^ | | 0.771 ^b^ | | -0.238 ^a^ | | -0.623 ^a^ | |  | | 0.368 | | <0.001 | | 0.265 |  |
| N-Acetyl-Proline | | Amino acid - related | | HMDB0094701 | | 0.158 ^a^ | | 0.562 ^a^ | | -0.328 ^a^ | | -0.392 ^a^ | |  | | 0.076 | | <0.001 | | 0.474 |  |
| N-Acetylputrescine | | Amino acid - related | | HMDB0002064 | | 0.137 ^ab^ | | 0.631 ^b^ | | -0.006 ^ab^ | | -0.762 ^a^ | |  | | 0.214 | | <0.001 | | 0.376 |  |
| Nudifloramide | | Pyridines | | HMDB0004193 | | 0.106 ^b^ | | 1.228 ^c^ | | -0.818 ^a^ | | -0.516 ^ab^ | |  | | 0.077 | | <0.001 | | 0.197 |  |
| Orotic acid | | Organic acids | | HMDB0000226 | | -0.280 ^a^ | | 0.395 ^a^ | | 0.064 ^a^ | | -0.179 ^a^ | |  | | 0.060 | | <0.001 | | 0.908 |  |
| PC aa C30:0 | | Glycerophospholipids | | HMDB0007869 | | 0.211 ^b^ | | 1.238 ^c^ | | -0.778 ^a^ | | -0.671 ^a^ | |  | | 0.124 | | <0.001 | | 0.465 |  |
| PC aa C32:1 | | Glycerophospholipids | | HMDB0007872 | | 0.253 ^b^ | | 1.192 ^c^ | | -0.854 ^a^ | | -0.590 ^a^ | |  | | 0.105 | | <0.001 | | 0.061 |  |
| PC aa C32:2 | | Glycerophospholipids | | HMDB0007874 | | 0.340 ^b^ | | 1.279 ^c^ | | -0.921 ^a^ | | -0.699 ^a^ | |  | | 0.004 | | <0.001 | | 0.073 |  |
| PC aa C34:1 | | Glycerophospholipids | | HMDB0007879 | | 0.307 ^b^ | | 1.124 ^c^ | | -0.650 ^a^ | | -0.781 ^a^ | |  | | 0.998 | | <0.001 | | 0.057 |  |
| PC aa C32:3 | | Glycerophospholipids | | HMDB0007875 | | 0.200 ^b^ | | 1.306 ^c^ | | -0.828 ^a^ | | -0.678 ^a^ | |  | | 0.316 | | <0.001 | | 0.248 |  |
| PC aa C34:2 | | Glycerophospholipids | | HMDB0007880 | | 0.287 ^b^ | | 1.136 ^c^ | | -0.590 ^a^ | | -0.833 ^a^ | |  | | 0.062 | | <0.001 | | 0.246 |  |
| PC aa C34:3 | | Glycerophospholipids | | HMDB0007881 | | 0.338 ^b^ | | 1.110 ^c^ | | -0.889 ^a^ | | -0.560 ^a^ | |  | | 0.181 | | <0.001 | | 0.066 |  |
| PC aa C34:4 | | Glycerophospholipids | | HMDB0007883 | | 0.315 ^b^ | | 1.079 ^c^ | | -0.965 ^a^ | | -0.430 ^a^ | |  | | 0.101 | | <0.001 | | 0.191 |  |
| PC aa C36:1 | | Glycerophospholipids | | HMDB0007887 | | 0.249 ^b^ | | 1.241 ^c^ | | -0.748 ^a^ | | -0.742 ^a^ | |  | | 0.022 | | <0.001 | | 0.057 |  |
| PC aa C36:3 | | Glycerophospholipids | | HMDB0007921 | | 0.193 ^b^ | | 1.230 ^c^ | | -0.749 ^a^ | | -0.674 ^a^ | |  | | 0.365 | | <0.001 | | 0.122 |  |
| PC aa C36:4 | | Glycerophospholipids | | HMDB0007889 | | 0.268 ^b^ | | 1.156 ^c^ | | -0.815 ^a^ | | -0.610 ^a^ | |  | | 0.217 | | <0.001 | | 0.722 |  |
| PC aa C36:5 | | Glycerophospholipids | | HMDB0007890 | | 0.158 ^b^ | | 1.204 ^c^ | | -0.768 ^a^ | | -0.594 ^a^ | |  | | 0.338 | | <0.001 | | 0.506 |  |
| PC aa C36:6 | | Glycerophospholipids | | HMDB0008690 | | 0.318 ^b^ | | 1.230 ^c^ | | -0.967 ^a^ | | -0.581 ^a^ | |  | | 0.155 | | <0.001 | | 0.939 |  |
| PC aa C38:0 | | Glycerophospholipids | | HMDB0007893 | | 0.246 ^b^ | | 1.286 ^c^ | | -0.825 ^a^ | | -0.707 ^a^ | |  | | 0.062 | | <0.001 | | 0.121 |  |
| PC aa C38:1 | | Glycerophospholipids | | HMDB0007894 | | 0.263 ^b^ | | 1.293 ^c^ | | -0.822 ^a^ | | -0.734 ^a^ | |  | | 0.089 | | <0.001 | | 0.228 |  |
| PC aa C38:3 | | Glycerophospholipids | | HMDB0008020 | | 0.282 ^b^ | | 1.286 ^c^ | | -0.862 ^a^ | | -0.706 ^a^ | |  | | 0.079 | | <0.001 | | 0.939 |  |
| PC aa C38:4 | | Glycerophospholipids | | HMDB0007988 | | 0.279 ^b^ | | 1.288 ^c^ | | -0.940 ^a^ | | -0.627 ^a^ | |  | | 0.050 | | <0.001 | | 0.561 |  |
| PC aa C38:5 | | Glycerophospholipids | | HMDB0007989 | | 0.224 ^b^ | | 1.269 ^c^ | | -0.914 ^a^ | | -0.579 ^a^ | |  | | 0.045 | | <0.001 | | 0.052 |  |
| PC aa C38:6 | | Glycerophospholipids | | HMDB0007991 | | 0.380 ^b^ | | 1.218 ^c^ | | -0.935 ^a^ | | -0.663 ^a^ | |  | | 0.057 | | <0.001 | | 0.236 |  |
| PC aa C40:1 | | Glycerophospholipids | | HMDB0007993 | | 0.118 ^b^ | | 1.261 ^c^ | | -0.707 ^a^ | | -0.673 ^a^ | |  | | 0.214 | | <0.001 | | 0.575 |  |
| PC aa C40:3 | | Glycerophospholipids | | HMDB0008086 | | 0.193 ^b^ | | 1.178 ^c^ | | -0.799 ^a^ | | -0.571 ^a^ | |  | | 0.156 | | <0.001 | | 0.707 |  |
| PC aa C40:5 | | Glycerophospholipids | | HMDB0008055 | | 0.304 ^b^ | | 1.280 ^c^ | | -0.953 ^a^ | | -0.631 ^a^ | |  | | 0.203 | | <0.001 | | 0.429 |  |
| PC aa C40:6 | | Glycerophospholipids | | HMDB0008057 | | 0.336 ^b^ | | 1.288 ^c^ | | -0.918 ^a^ | | -0.706 ^a^ | |  | | 0.131 | | <0.001 | | 0.111 |  |
| PC aa C42:1 | | Glycerophospholipids | | HMDB0008059 | | 0.102 ^b^ | | 1.258 ^c^ | | -0.731 ^a^ | | -0.629 ^a^ | |  | | 0.821 | | <0.001 | | 0.860 |  |
| PC aa C42:5 | | Glycerophospholipids | | HMDB0008257 | | 0.275 ^b^ | | 1.216 ^c^ | | -0.895 ^a^ | | -0.596 ^a^ | |  | | 0.129 | | <0.001 | | 0.420 |  |
| PC aa C42:6 | | Glycerophospholipids | | HMDB0008288 | | 0.289 ^b^ | | 1.020 ^b^ | | -0.817 ^a^ | | -0.491 ^a^ | |  | | 0.090 | | <0.001 | | 0.793 |  |
| PC ae C30:0 | | Glycerophospholipids | | HMDB0013341 | | 0.180 ^b^ | | 1.235 ^c^ | | -0.722 ^a^ | | -0.694 ^a^ | |  | | 0.043 | | <0.001 | | 0.388 |  |
| PC ae C30:1 | | Glycerophospholipids | | HMDB0013402 | | 0.175 ^b^ | | 1.165 ^c^ | | -0.608 ^a^ | | -0.731 ^a^ | |  | | 0.025 | | <0.001 | | 0.651 |  |
| PC ae C34:0 | | Glycerophospholipids | | HMDB0013405 | | 0.201 ^b^ | | 1.267 ^c^ | | -0.740 ^a^ | | -0.728 ^a^ | |  | | 0.135 | | <0.001 | | 0.078 |  |
| PC ae C34:1 | | Glycerophospholipids | | HMDB0013412 | | 0.250 ^b^ | | 1.244 ^c^ | | -0.774 ^a^ | | -0.720 ^a^ | |  | | 0.105 | | <0.001 | | 0.703 |  |
| PC ae C34:2 | | Glycerophospholipids | | HMDB0011151 | | 0.203 ^b^ | | 1.212 ^c^ | | -0.692 ^a^ | | -0.723 ^a^ | |  | | 0.614 | | <0.001 | | 0.497 |  |
| PC ae C34:3 | | Glycerophospholipids | | HMDB0013413 | | 0.230 ^b^ | | 1.189 ^c^ | | -0.670 ^a^ | | -0.749 ^a^ | |  | | 0.164 | | <0.001 | | 0.823 |  |
| PC ae C36:3 | | Glycerophospholipids | | HMDB0013425 | | 0.191 ^b^ | | 1.261 ^c^ | | -0.772 ^a^ | | -0.680 ^a^ | |  | | 0.432 | | <0.001 | | 0.585 |  |
| PC ae C36:4 | | Glycerophospholipids | | HMDB0013407 | | 0.257 ^b^ | | 1.275 ^c^ | | -0.830 ^a^ | | -0.702 ^a^ | |  | | 0.025 | | <0.001 | | 0.242 |  |
| PC ae C36:5 | | Glycerophospholipids | | HMDB0013415 | | 0.244 ^b^ | | 1.221 ^c^ | | -0.706 ^a^ | | -0.758 ^a^ | |  | | 0.405 | | <0.001 | | 0.530 |  |
| PC ae C38:0 | | Glycerophospholipids | | HMDB0013408 | | 0.275 ^b^ | | 1.241 ^c^ | | -0.926 ^a^ | | -0.591 ^a^ | |  | | 0.006 | | <0.001 | | 0.052 |  |
| PC ae C38:2 | | Glycerophospholipids | | HMDB0013431 | | -0.084 ^a^ | | 1.050 ^b^ | | -0.307 ^a^ | | -0.659 ^a^ | |  | | 0.188 | | <0.001 | | 0.600 |  |
| PC ae C38:4 | | Glycerophospholipids | | HMDB0013420 | | 0.197 ^b^ | | 1.301 ^c^ | | -0.824 ^a^ | | -0.674 ^a^ | |  | | 0.450 | | <0.001 | | 0.276 |  |
| PC ae C38:5 | | Glycerophospholipids | | HMDB0013432 | | 0.241 ^b^ | | 1.298 ^c^ | | -0.861 ^a^ | | -0.678 ^a^ | |  | | 0.183 | | <0.001 | | 0.203 |  |
| PC ae C38:6 | | Glycerophospholipids | | HMDB0013409 | | 0.273 ^b^ | | 1.278 ^c^ | | -0.870 ^a^ | | -0.682 ^a^ | |  | | 0.042 | | <0.001 | | 0.157 |  |
| PC ae C40:1 | | Glycerophospholipids | | HMDB0013433 | | 0.292 ^b^ | | 1.237 ^c^ | | -0.865 ^a^ | | -0.664 ^a^ | |  | | 0.105 | | <0.001 | | 0.855 |  |
| PC ae C40:6 | | Glycerophospholipids | | HMDB0013422 | | 0.251 ^b^ | | 1.308 ^c^ | | -0.843 ^a^ | | -0.716 ^a^ | |  | | 0.050 | | <0.001 | | 0.196 |  |
| PC ae C42:0 | | Glycerophospholipids | | HMDB0013423 | | 0.198 ^b^ | | 1.188 ^c^ | | -0.823 ^a^ | | -0.563 ^a^ | |  | | 0.348 | | <0.001 | | 0.334 |  |
| PC ae C42:2 | | Glycerophospholipids | | HMDB0013438 | | 0.248 ^b^ | | 1.235 ^c^ | | -0.842 ^a^ | | -0.641 ^a^ | |  | | 0.200 | | <0.001 | | 0.114 |  |
| PC ae C42:1 | | Glycerophospholipids | | HMDB0013434 | | 0.169 ^b^ | | 1.293 ^c^ | | -0.860 ^a^ | | -0.603 ^a^ | |  | | 0.140 | | <0.001 | | 0.958 |  |
| PC ae C42:3 | | Glycerophospholipids | | HMDB0013458 | | 0.359 ^b^ | | 1.169 ^c^ | | -0.849 ^a^ | | -0.678 ^a^ | |  | | 0.221 | | <0.001 | | 0.291 |  |
| PC ae C44:4 | | Glycerophospholipids | | HMDB0013453 | | 0.179 ^b^ | | 1.228 ^c^ | | -0.712 ^a^ | | -0.695 ^a^ | |  | | 0.431 | | <0.001 | | 0.252 |  |
| PC ae C44:5 | | Glycerophospholipids | | HMDB0013456 | | 0.274 ^b^ | | 1.223 ^c^ | | -0.689 ^a^ | | -0.807 ^a^ | |  | | 0.265 | | <0.001 | | 0.318 |  |
| p-Cresol sulfate | | Organic acids | | HMDB0011635 | | 0.149 ^b^ | | 1.236 ^c^ | | -0.666 ^a^ | | -0.720 ^a^ | |  | | 0.226 | | <0.001 | | 0.196 |  |
| Phenylacetic acid | | Organic acids | | HMDB0000209 | | 0.133 ^a^ | | 0.976 ^b^ | | -0.568 ^a^ | | -0.542 ^a^ | |  | | 0.332 | | <0.001 | | 0.226 |  |
| Phenylalanine | | Biogenic Amines | | HMDB0000159 | | 0.319 ^b^ | | 1.183 ^c^ | | -0.870 ^a^ | | -0.632 ^a^ | |  | | 0.036 | | <0.001 | | 0.793 |  |
| Picolinic acid | | Organic acids | | HMDB0002243 | | 0.501 ^b^ | | 1.014 ^b^ | | -0.863 ^a^ | | -0.652 ^a^ | |  | | 0.514 | | <0.001 | | 0.983 |  |
| Pipecolic acid | | Organic acids | | HMDB0000070 | | -0.348 ^a^ | | 0.909 ^b^ | | -0.177 ^a^ | | -0.383 ^a^ | |  | | 0.061 | | <0.001 | | 0.289 |  |
| Proline | | Amnio acid | | HMDB0000162 | | 0.467 ^b^ | | 0.490 ^b^ | | -0.402 ^ab^ | | -0.556 ^a^ | |  | | 0.175 | | <0.001 | | 0.386 |  |
| Propionic acid | | Short chain fatty acid | | HMDB0000237 | | 0.172 ^bc^ | | 0.909 ^c^ | | -0.734 ^a^ | | -0.347 ^ab^ | |  | | 0.446 | | <0.001 | | 0.257 |  |
| Putrescine | | Biogenic Amines | | HMDB0001414 | | 0.184 ^b^ | | 1.115 ^c^ | | -0.863 ^a^ | | -0.436 ^ab^ | |  | | 0.181 | | <0.001 | | 0.413 |  |
| Pyruvic acid | | Organic acids | | HMDB0000243 | | 0.470 ^b^ | | 0.682 ^b^ | | -0.673 ^a^ | | -0.479 ^a^ | |  | | 0.007 | | <0.001 | | 0.939 |  |
| Quinolinic acid | | Organic acids | | HMDB0000232 | | 0.204 ^ab^ | | 0.939 ^b^ | | -0.574 ^a^ | | -0.569 ^a^ | |  | | 0.034 | | <0.001 | | 0.053 |  |
| Serine | | Amino acid | | HMDB0000187 | | 0.359 ^b^ | | 0.908 ^b^ | | -0.721 ^a^ | | -0.546 ^a^ | |  | | 0.063 | | <0.001 | | 0.209 |  |
| Serotonin | | Biogenic Amines | | HMDB0000259 | | 0.273 ^ab^ | | 0.781 ^b^ | | -0.491 ^a^ | | -0.564 ^a^ | |  | | 0.526 | | <0.001 | | 0.425 |  |
| SM C16:0 | | Sphingomyelins | | HMDB0010169 | | 0.266 ^b^ | | 1.284 ^c^ | | -0.816 ^a^ | | -0.734 ^a^ | |  | | 0.450 | | <0.001 | | 0.218 |  |
| SM C18:0 | | Sphingomyelins | | HMDB0001348 | | 0.273 ^b^ | | 1.242 ^c^ | | -0.845 ^a^ | | -0.671 ^a^ | |  | | 0.312 | | <0.001 | | 0.233 |  |
| SM C20:2 | | Sphingomyelins | | HMDB0013465 | | 0.189 ^b^ | | 1.277 ^c^ | | -0.979 ^a^ | | -0.486 ^a^ | |  | | 0.079 | | <0.001 | | 0.621 |  |
| SM C24:0 | | Sphingomyelins | | HMDB0011697 | | 0.260 ^b^ | | 1.318 ^c^ | | -0.874 ^a^ | | -0.704 ^a^ | |  | | 0.212 | | <0.001 | | 0.866 |  |
| SM C24:1 | | Sphingomyelins | | HMDB0012107 | | 0.284 ^b^ | | 1.327 ^c^ | | -0.901 ^a^ | | -0.709 ^a^ | |  | | 0.551 | | <0.001 | | 0.394 |  |
| SM C26:1 | | Sphingomyelins | | HMDB0013461 | | 0.172 ^b^ | | 1.216 ^c^ | | -0.766 ^a^ | | -0.622 ^a^ | |  | | 0.161 | | <0.001 | | 0.556 |  |
| SM(OH) C14:1 | | Sphingomyelins | | HMDB0013462 | | 0.125 ^b^ | | 1.167 ^c^ | | -0.616 ^a^ | | -0.677 ^a^ | |  | | 0.171 | | <0.001 | | 0.340 |  |
| SM(OH) C16:1 | | Sphingomyelins | | HMDB0013463 | | 0.252 ^b^ | | 1.298 ^c^ | | -0.829 ^a^ | | -0.721 ^a^ | |  | | 0.105 | | <0.001 | | 0.265 |  |
| SM(OH) C22:1 | | Sphingomyelins | | HMDB0013466 | | 0.071 ^b^ | | 1.264 ^c^ | | -0.672 ^a^ | | -0.663 ^a^ | |  | | 0.155 | | <0.001 | | 0.786 |  |
| SM(OH) C24:1 | | Sphingomyelins | | HMDB0013469 | | 0.211 ^b^ | | 1.274 ^c^ | | -0.773 ^a^ | | -0.712 ^a^ | |  | | 0.102 | | <0.001 | | 0.857 |  |
| Succinic acid | | Organic acids | | HMDB0000254 | | -0.443 ^a^ | | 0.593 ^b^ | | -0.008 ^ab^ | | -0.142 ^ab^ | |  | | 0.312 | | <0.001 | | 0.228 |  |
| Taurine | | Amino acid - related | | HMDB0000251 | | 0.075 ^a^ | | 0.981 ^b^ | | -0.592 ^a^ | | -0.464 ^a^ | |  | | 0.161 | | <0.001 | | 0.819 |  |
| Symmetric dimethylarginine | | Amino acid - related | | HMDB0001539 | | 0.202 ^ab^ | | 0.911 ^b^ | | -0.621 ^a^ | | -0.493 ^a^ | |  | | 0.183 | | <0.001 | | 0.961 |  |
| TCDCA | | Bile acid | | Not available | | -0.216 ^a^ | | 0.330 ^a^ | | -0.369 ^a^ | | 0.255 ^a^ | |  | | 0.307 | | <0.001 | | 0.174 |  |
| **Down regulated** | |  | |  | |  | |  | |  | |  | |  | |  | |  | |  |  |
| Benzoic acid | | Organic acids | | HMDB0001870 | | -0.588 ^a^ | | 0.467 ^b^ | | 0.352 ^ab^ | | -0.231 ^ab^ | |  | | 0.007 | | <0.001 | | 0.055 |  |
| Caproic acid | | Organic acids | | HMDB0000535 | | -0.249 ^a^ | | 0.134 ^a^ | | -0.044 ^a^ | | 0.158 ^a^ | |  | | 0.041 | | <0.001 | | 0.905 |  |
| CE(14:0) | | Cholesterol Esters | | HMDB06725 | | 0.154 ^a^ | | -0.545 ^a^ | | 0.330 ^a^ | | 0.061 ^a^ | |  | | 0.023 | | <0.001 | | 0.052 |  |
| CE(16:0) | | Cholesterol Esters | | HMDB05188 | | 0.187 ^a^ | | -0.644 ^a^ | | 0.299 ^a^ | | 0.158 ^a^ | |  | | 0.004 | | <0.001 | | 0.490 |  |
| CE(16:1) | | Cholesterol Esters | | HMDB05197 | | 0.207 ^a^ | | -0.432 ^a^ | | 0.050 ^a^ | | 0.175 ^a^ | |  | | 0.048 | | <0.001 | | 0.269 |  |
| CE(17:1) | | Cholesterol Esters | | HMDB60060 | | -0.027 ^b^ | | -1.042 ^a^ | | 0.607 ^b^ | | 0.462 ^b^ | |  | | 0.023 | | <0.001 | | 0.221 |  |
| CE(18:0) | | Cholesterol Esters | | HMDB10368 | | -0.031 ^b^ | | -1.099 ^a^ | | 0.721 ^b^ | | 0.409 ^b^ | |  | | 0.022 | | <0.001 | | 0.203 |  |
| CE(20:1) | | Cholesterol Esters | | HMDB05194 | | -0.139 ^a^ | | -0.776 ^a^ | | 0.969 ^b^ | | -0.054 ^a^ | |  | | 0.031 | | <0.001 | | 0.213 |  |
| Cystathionine | | Organic acids | | HMDB0000099 | | -0.145 ^a^ | | 0.059 ^a^ | | 0.365 ^a^ | | -0.279 ^a^ | |  | | 0.014 | | <0.001 | | 0.561 |  |
| DG(16:0_16:0) | | Diglycerides | | HMDB07098 | | 0.174 ^b^ | | -0.949 ^a^ | | 0.462 ^b^ | | 0.314 ^b^ | |  | | 0.033 | | <0.001 | | 0.440 |  |
| DG(16:0_16:1) | | Diglycerides | | HMDB07099 | | 0.140 ^ab^ | | -0.616 ^a^ | | 0.078 ^ab^ | | 0.399 ^b^ | |  | | 0.029 | | <0.001 | | 0.061 |  |
| DG(16:0_18:1) | | Diglycerides | | HMDB07101 | | 0.497 ^b^ | | -0.942 ^a^ | | 0.361 ^b^ | | 0.084 ^b^ | |  | | 0.032 | | <0.001 | | 0.252 |  |
| DG(16:1_18:2) | | Diglycerides | | HMDB07132 | | -0.241 ^a^ | | -0.188 ^a^ | | 0.467 ^a^ | | -0.038 ^a^ | |  | | 0.162 | | <0.001 | | 0.183 |  |
| DG(16:1_18:1) | | Diglycerides | | HMDB07130 | | 0.605 ^b^ | | -0.645 ^a^ | | -0.035 ^ab^ | | 0.075 ^ab^ | |  | | 0.041 | | <0.001 | | 0.202 |  |
| DG(16:0_18:2) | | Diglycerides | | HMDB07103 | | 0.083 ^a^ | | -0.476 ^a^ | | 0.445 ^a^ | | -0.052 ^a^ | |  | | 0.036 | | <0.001 | | 0.656 |  |
| DG(16:1_20:0) | | Diglycerides | | HMDB07136 | | 0.387 ^b^ | | -0.631 ^a^ | | -0.005 ^ab^ | | 0.249 ^ab^ | |  | | 0.023 | | <0.001 | | 0.057 |  |
| DG(17:0_17:1) | | Diglycerides | | Not available | | 0.682 ^b^ | | -0.682 ^a^ | | -0.135 ^ab^ | | 0.136 ^ab^ | |  | | 0.084 | | <0.001 | | 0.213 |  |
| DG(18:1_20:3) | | Diglycerides | | HMDB07197 | | 0.363 ^b^ | | -0.835 ^a^ | | 0.326 ^b^ | | 0.147 ^b^ | |  | | 0.004 | | <0.001 | | 0.213 |  |
| DG(18:1_20:4) | | Diglycerides | | HMDB07199 | | 0.530 ^b^ | | -0.867 ^a^ | | 0.433 ^b^ | | -0.097 ^ab^ | |  | | 0.033 | | <0.001 | | 0.221 |  |
| DG(18:2_18:2) | | Diglycerides | | HMDB07248 | | -0.016 ^a^ | | -0.189 ^a^ | | 0.405 ^a^ | | -0.200 ^a^ | |  | | 0.033 | | <0.001 | | 0.422 |  |
| Fumaric acid | | Organic acids | | HMDB0000134 | | -0.050 ^a^ | | 0.125 ^a^ | | 0.295 ^a^ | | -0.370 ^a^ | |  | | 0.039 | | <0.001 | | 0.128 |  |
| Hypoxanthine | | Nucleobase | | HMDB0000157 | | -0.187 ^b^ | | -1.026 ^a^ | | 0.364 ^bc^ | | 0.849 ^c^ | |  | | 0.033 | | <0.001 | | 0.122 |  |
| Inosine | | Nucleoside | | HMDB0000195 | | -0.132 ^ab^ | | -0.762 ^a^ | | 0.251 ^b^ | | 0.644 ^b^ | |  | | 0.023 | | <0.001 | | 0.291 |  |
| Shikimic acid | | Organic acids | | HMDB0003070 | | -0.248 ^a^ | | 0.188 ^a^ | | 0.297 ^a^ | | -0.237 ^a^ | |  | | 0.061 | | <0.001 | | 0.329 |  |
| TCA | | Organic acids | | HMDB0042048 | | -0.210 ^a^ | | 0.104 ^a^ | | -0.287 ^a^ | | 0.393 ^a^ | |  | | 0.084 | | <0.001 | | 0.449 |  |
| TG(14:0_34:3) | | Triglycerides | | HMDB0042073 | | -0.134 ^ab^ | | -0.886 ^a^ | | 0.543 ^b^ | | 0.477 ^b^ | |  | | 0.229 | | <0.001 | | 0.840 |  |
| TG(14:0_36:2) | | Triglycerides | | HMDB0042081 | | -0.309 ^b^ | | -1.044 ^a^ | | 0.637 ^c^ | | 0.716 ^c^ | |  | | 0.220 | | <0.001 | | 0.138 |  |
| TG(14:0_36:3) | | Triglycerides | | HMDB0042133 | | -0.341 ^a^ | | -0.974 ^a^ | | 0.688 ^b^ | | 0.627 ^b^ | |  | | 0.220 | | <0.001 | | 0.340 |  |
| TG(14:0_36:4) | | Triglycerides | | HMDB0042082 | | -0.518 ^a^ | | -0.657 ^a^ | | 0.658 ^b^ | | 0.518 ^b^ | |  | | 0.155 | | <0.001 | | 0.228 |  |
| TG(16:0_32:2) | | Triglycerides | | HMDB0005376 | | -0.219 ^ab^ | | -0.912 ^a^ | | 0.559 ^b^ | | 0.572 ^b^ | |  | | 0.161 | | <0.001 | | 0.394 |  |
| TG(16:0_34:1) | | Triglycerides | | HMDB0005360 | | -0.453 ^a^ | | -0.938 ^a^ | | 0.787 ^b^ | | 0.605 ^b^ | |  | | 0.257 | | <0.001 | | 0.494 |  |
| TG(16:0_34:2) | | Triglycerides | | HMDB0005362 | | -0.298 ^b^ | | -1.152 ^a^ | | 0.843 ^c^ | | 0.607 ^c^ | |  | | 0.331 | | <0.001 | | 0.211 |  |
| TG(16:0_34:3) | | Triglycerides | | HMDB0005379 | | -0.198 ^ab^ | | -0.921 ^a^ | | 0.493 ^b^ | | 0.626 ^b^ | |  | | 0.499 | | <0.001 | | 0.429 |  |
| TG(16:0_34:4) | | Triglycerides | | HMDB0043846 | | -0.051 ^b^ | | -0.929 ^a^ | | 0.424 ^b^ | | 0.556 ^b^ | |  | | 0.330 | | <0.001 | | 0.422 |  |
| TG(16:0_35:3) | | Triglycerides | | HMDB0043867 | | -0.232 ^b^ | | -1.043 ^a^ | | 0.771 ^c^ | | 0.504 ^bc^ | |  | | 0.195 | | <0.001 | | 0.052 |  |
| TG(16:0_36:2) | | Triglycerides | | HMDB0005369 | | -0.356 ^b^ | | -1.106 ^a^ | | 0.898 ^c^ | | 0.564 ^c^ | |  | | 0.204 | | <0.001 | | 0.352 |  |
| TG(16:0_36:3) | | Triglycerides | | HMDB0005384 | | -0.314 ^ab^ | | -0.872 ^a^ | | 0.797 ^c^ | | 0.389 ^bc^ | |  | | 0.120 | | <0.001 | | 0.364 |  |
| TG(16:0_36:4) | | Triglycerides | | HMDB0005363 | | -0.352 ^ab^ | | -0.718 ^a^ | | 0.807 ^c^ | | 0.262 ^bc^ | |  | | 0.510 | | <0.001 | | 0.334 |  |
| TG(16:0_36:5) | | Triglycerides | | HMDB0005380 | | -0.301 ^ab^ | | -0.873 ^a^ | | 0.804 ^c^ | | 0.369 ^bc^ | |  | | 0.462 | | <0.001 | | 0.510 |  |
| TG(16:0_38:3) | | Triglycerides | | HMDB0005389 | | -0.319 ^b^ | | -1.126 ^a^ | | 0.970 ^c^ | | 0.475 ^c^ | |  | | 0.252 | | <0.001 | | 0.265 |  |
| TG(16:0_38:4) | | Triglycerides | | HMDB0005370 | | -0.211 ^b^ | | -1.084 ^a^ | | 0.841 ^c^ | | 0.455 ^bc^ | |  | | 0.291 | | <0.001 | | 0.179 |  |
| TG(16:0_38:5) | | Triglycerides | | HMDB0005385 | | -0.070 ^b^ | | -1.003 ^a^ | | 0.664 ^b^ | | 0.409 ^b^ | |  | | 0.121 | | <0.001 | | 0.422 |  |
| TG(16:0_38:6) | | Triglycerides | | HMDB0005391 | | -0.062 ^ab^ | | -0.745 ^a^ | | 0.473 ^b^ | | 0.335 ^b^ | |  | | 0.205 | | 0.001 | | 0.070 |  |
| TG(16:0_40:6) | | Triglycerides | | HMDB0043939 | | -0.141 ^ab^ | | -0.901 ^a^ | | 0.594 ^b^ | | 0.449 ^b^ | |  | | 0.235 | | 0.001 | | 0.183 |  |
| TG(16:0_40:7) | | Triglycerides | | HMDB0044107 | | -0.162 ^ab^ | | -0.908 ^a^ | | 0.476 ^b^ | | 0.593 ^b^ | |  | | 0.171 | | 0.001 | | 0.388 |  |
| TG(16:0_40:8) | | Triglycerides | | HMDB0005392 | | -0.189 ^a^ | | -0.360 ^a^ | | 0.201 ^a^ | | 0.348 ^a^ | |  | | 0.392 | | 0.001 | | 0.494 |  |
| TG(16:1_34:1) | | Triglycerides | | HMDB0010423 | | -0.376 ^b^ | | -1.099 ^a^ | | 0.718 ^c^ | | 0.757 ^c^ | |  | | 0.556 | | 0.001 | | 0.927 |  |
| TG(16:1_34:2) | | Triglycerides | | HMDB0048464 | | -0.299 ^a^ | | -0.971 ^a^ | | 0.531 ^b^ | | 0.739 ^b^ | |  | | 0.160 | | 0.001 | | 0.720 |  |
| TG(16:1_34:3) | | Triglycerides | | HMDB0048465 | | -0.230 ^ab^ | | -0.846 ^a^ | | 0.339 ^bc^ | | 0.737 ^c^ | |  | | 0.448 | | 0.001 | | 0.394 |  |
| TG(16:1_36:1) | | Triglycerides | | HMDB0048460 | | -0.492 ^a^ | | -0.889 ^a^ | | 0.707 ^b^ | | 0.673 ^b^ | |  | | 0.020 | | 0.001 | | 0.307 |  |
| TG(16:1_36:2) | | Triglycerides | | HMDB0005425 | | -0.330 ^b^ | | -1.058 ^a^ | | 0.733 ^c^ | | 0.655 ^c^ | |  | | 0.477 | | 0.001 | | 0.793 |  |
| TG(16:1_36:3) | | Triglycerides | | HMDB0048487 | | -0.322 ^ab^ | | -0.807 ^a^ | | 0.519 ^bc^ | | 0.611 ^c^ | |  | | 0.016 | | 0.001 | | 0.578 |  |
| TG(16:1_36:4) | | Triglycerides | | HMDB0048495 | | -0.297 ^ab^ | | -0.657 ^a^ | | 0.395 ^b^ | | 0.559 ^b^ | |  | | 0.536 | | 0.001 | | 0.318 |  |
| TG(16:1_38:3) | | Triglycerides | | HMDB0048620 | | -0.345 ^b^ | | -1.054 ^a^ | | 0.799 ^c^ | | 0.601 ^c^ | |  | | 0.951 | | 0.002 | | 0.857 |  |
| TG(16:1_38:4) | | Triglycerides | | HMDB0048615 | | -0.087 ^b^ | | -1.127 ^a^ | | 0.624 ^b^ | | 0.590 ^b^ | |  | | 0.212 | | 0.002 | | 0.388 |  |
| TG(16:1_38:5) | | Triglycerides | | HMDB0048622 | | -0.173 ^b^ | | -1.021 ^a^ | | 0.562 ^bc^ | | 0.631 ^c^ | |  | | 0.773 | | 0.002 | | 0.497 |  |
| TG(17:0_36:3) | | Triglycerides | | HMDB0050323 | | -0.327 ^b^ | | -1.172 ^a^ | | 0.848 ^c^ | | 0.651 ^c^ | |  | | 0.858 | | 0.003 | | 0.836 |  |
| TG(17:0_36:4) | | Triglycerides | | HMDB0055545 | | -0.439 ^a^ | | -1.022 ^a^ | | 1.191 ^c^ | | 0.270 ^b^ | |  | | 0.391 | | 0.003 | | 0.742 |  |
| TG(17:1_36:3) | | Triglycerides | | HMDB0055545 | | -0.352 ^a^ | | -1.036 ^a^ | | 0.834 ^b^ | | 0.554 ^b^ | |  | | 0.119 | | 0.003 | | 0.201 |  |
| TG(17:1_36:4) | | Triglycerides | | HMDB0043044 | | -0.263 ^ab^ | | -0.870 ^a^ | | 0.775 ^c^ | | 0.359 ^bc^ | |  | | 0.167 | | 0.003 | | 0.357 |  |
| TG(18:0_34:2) | | Triglycerides | | HMDB0044682 | | -0.207 ^b^ | | -1.306 ^a^ | | 0.940 ^c^ | | 0.574 ^c^ | |  | | 0.150 | | 0.003 | | 0.267 |  |
| TG(18:0_34:3) | | Triglycerides | | HMDB0010432 | | -0.217 ^b^ | | -1.082 ^a^ | | 0.630 ^c^ | | 0.669 ^c^ | |  | | 0.900 | | 0.003 | | 0.471 |  |
| TG(18:0_36:2) | | Triglycerides | | HMDB0005397 | | -0.467 ^a^ | | -0.941 ^a^ | | 0.798 ^b^ | | 0.610 ^b^ | |  | | 0.701 | | 0.003 | | 0.985 |  |
| TG(18:0_36:3) | | Triglycerides | | HMDB0005405 | | -0.338 ^b^ | | -1.202 ^a^ | | 1.081 ^d^ | | 0.459 ^c^ | |  | | 0.861 | | 0.003 | | 0.087 |  |
| TG(18:0_36:4) | | Triglycerides | | HMDB0005411 | | -0.527 ^b^ | | -1.064 ^a^ | | 1.211 ^d^ | | 0.380 ^c^ | |  | | 0.068 | | 0.003 | | 0.184 |  |
| TG(18:0_36:5) | | Triglycerides | | HMDB0044687 | | -0.200 ^b^ | | -1.233 ^a^ | | 0.970 ^c^ | | 0.463 ^c^ | |  | | 0.330 | | 0.003 | | 0.334 |  |
| TG(18:0_38:6) | | Triglycerides | | HMDB0005412 | | -0.037 ^ab^ | | -0.825 ^a^ | | 0.522 ^b^ | | 0.340 ^b^ | |  | | 0.721 | | 0.004 | | 0.196 |  |
| TG(18:1_30:2) | | Triglycerides | | HMDB0042543 | | 0.126 ^b^ | | -0.983 ^a^ | | 0.481 ^b^ | | 0.376 ^b^ | |  | | 0.942 | | 0.004 | | 0.276 |  |
| TG(18:1_32:1) | | Triglycerides | | HMDB0010434 | | -0.358 ^b^ | | -1.058 ^a^ | | 0.699 ^c^ | | 0.718 ^c^ | |  | | 0.558 | | 0.005 | | 0.616 |  |
| TG(18:1_32:2) | | Triglycerides | | HMDB0010436 | | -0.283 ^b^ | | -1.044 ^a^ | | 0.605 ^c^ | | 0.721 ^c^ | |  | | 0.156 | | 0.005 | | 0.569 |  |
| TG(18:1_32:3) | | Triglycerides | | HMDB0049079 | | -0.164 ^b^ | | -1.000 ^a^ | | 0.562 ^b^ | | 0.602 ^b^ | |  | | 0.159 | | 0.005 | | 0.427 |  |
| TG(18:1_33:2) | | Triglycerides | | HMDB0049099 | | -0.283 ^a^ | | -1.017 ^a^ | | 0.793 ^b^ | | 0.506 ^b^ | |  | | 0.652 | | 0.005 | | 0.183 |  |
| TG(18:1_34:1) | | Triglycerides | | HMDB0010437 | | -0.336 ^b^ | | -1.122 ^a^ | | 0.905 ^c^ | | 0.553 ^c^ | |  | | 0.937 | | 0.005 | | 0.866 |  |
| TG(18:1_34:2) | | Triglycerides | | HMDB0010439 | | -0.355 ^a^ | | -0.906 ^a^ | | 0.777 ^b^ | | 0.484 ^b^ | |  | | 0.083 | | 0.005 | | 0.196 |  |
| TG(18:1_34:3) | | Triglycerides | | HMDB0049121 | | -0.371 ^a^ | | -0.840 ^a^ | | 0.623 ^b^ | | 0.588 ^b^ | |  | | 0.217 | | 0.005 | | 0.196 |  |
| TG(18:1_34:4) | | Triglycerides | | HMDB0049129 | | -0.133 ^ab^ | | -0.900 ^a^ | | 0.439 ^b^ | | 0.594 ^b^ | |  | | 0.043 | | 0.006 | | 0.053 |  |
| TG(18:1_35:2) | | Triglycerides | | HMDB0049101 | | -0.366 ^a^ | | -1.018 ^a^ | | 0.822 ^b^ | | 0.563 ^b^ | |  | | 0.815 | | 0.006 | | 0.087 |  |
| TG(18:1_35:3) | | Triglycerides | | HMDB0049096 | | -0.419 ^a^ | | -0.990 ^a^ | | 0.816 ^b^ | | 0.593 ^b^ | |  | | 0.466 | | 0.007 | | 0.793 |  |
| TG(18:1_36:1) | | Triglycerides | | HMDB0049116 | | -0.491 ^a^ | | -0.988 ^a^ | | 0.854 ^b^ | | 0.624 ^b^ | |  | | 0.814 | | 0.007 | | 0.869 |  |
| TG(18:1_36:2) | | Triglycerides | | HMDB0010447 | | -0.354 ^b^ | | -1.177 ^a^ | | 0.867 ^c^ | | 0.665 ^c^ | |  | | 0.552 | | 0.008 | | 0.887 |  |
| TG(18:1_36:3) | | Triglycerides | | HMDB0049142 | | -0.401 ^a^ | | -0.987 ^a^ | | 0.852 ^b^ | | 0.537 ^b^ | |  | | 0.062 | | 0.009 | | 0.201 |  |
| TG(18:1_36:4) | | Triglycerides | | HMDB0049150 | | -0.397 ^a^ | | -1.005 ^a^ | | 1.004 ^b^ | | 0.398 ^b^ | |  | | 0.861 | | 0.010 | | 0.473 |  |
| TG(18:1_36:5) | | Triglycerides | | HMDB0049276 | | -0.335 ^a^ | | -1.032 ^a^ | | 0.919 ^b^ | | 0.448 ^b^ | |  | | 0.229 | | 0.010 | | 0.490 |  |
| TG(18:1_36:6) | | Triglycerides | | HMDB0049402 | | -0.038 ^b^ | | -1.231 ^a^ | | 0.677 ^c^ | | 0.593 ^bc^ | |  | | 0.556 | | 0.011 | | 0.840 |  |
| TG(18:1_38:5) | | Triglycerides | | HMDB0049390 | | -0.151 ^ab^ | | -0.924 ^a^ | | 0.584 ^b^ | | 0.491 ^b^ | |  | | 0.286 | | 0.012 | | 0.192 |  |
| TG(18:1_38:6) | | Triglycerides | | HMDB0049397 | | -0.046 ^ab^ | | -0.659 ^a^ | | 0.370 ^b^ | | 0.334 ^b^ | |  | | 0.698 | | 0.013 | | 0.121 |  |
| TG(18:1_38:7) | | Triglycerides | | HMDB0049423 | | -0.066 ^ab^ | | -0.579 ^a^ | | 0.228 ^ab^ | | 0.418 ^b^ | |  | | 0.478 | | 0.013 | | 0.265 |  |
| TG(18:2_32:0) | | Triglycerides | | HMDB0052410 | | -0.331 ^b^ | | -1.185 ^a^ | | 0.970 ^c^ | | 0.546 ^c^ | |  | | 0.361 | | 0.014 | | 0.565 |  |
| TG(18:2_32:1) | | Triglycerides | | HMDB0052515 | | -0.296 ^a^ | | -0.895 ^a^ | | 0.522 ^b^ | | 0.670 ^b^ | |  | | 0.264 | | 0.017 | | 0.470 |  |
| TG(18:2_32:2) | | Triglycerides | | HMDB0010470 | | -0.215 ^ab^ | | -0.890 ^a^ | | 0.448 ^bc^ | | 0.657 ^c^ | |  | | 0.162 | | 0.024 | | 0.214 |  |
| TG(18:2_33:1) | | Triglycerides | | HMDB0011701 | | -0.234 ^b^ | | -1.003 ^a^ | | 0.815 ^c^ | | 0.422 ^bc^ | |  | | 0.893 | | 0.025 | | 0.334 |  |
| TG(18:2_33:2) | | Triglycerides | | HMDB0052411 | | -0.526 ^a^ | | -0.985 ^a^ | | 1.048 ^b^ | | 0.463 ^b^ | |  | | 0.633 | | 0.027 | | 0.571 |  |
| TG(18:2_34:1) | | Triglycerides | | HMDB0052530 | | -0.289 ^ab^ | | -0.917 ^a^ | | 0.784 ^c^ | | 0.422 ^bc^ | |  | | 0.362 | | 0.029 | | 0.478 |  |
| TG(18:2_34:2) | | Triglycerides | | HMDB0052426 | | -0.388 ^ab^ | | -0.720 ^a^ | | 0.784 ^c^ | | 0.324 ^bc^ | |  | | 0.235 | | 0.035 | | 0.137 |  |
| TG(18:2_34:3) | | Triglycerides | | HMDB0052427 | | -0.394 ^ab^ | | -0.625 ^a^ | | 0.585 ^c^ | | 0.434 ^bc^ | |  | | 0.417 | | 0.036 | | 0.618 |  |
| TG(18:2_34:4) | | Triglycerides | | HMDB0052435 | | -0.253 ^ab^ | | -0.649 ^a^ | | 0.449 ^b^ | | 0.453 ^b^ | |  | | 0.448 | | 0.038 | | 0.506 |  |
| TG(18:2_35:1) | | Triglycerides | | HMDB0050323 | | -0.472 ^b^ | | -1.096 ^a^ | | 1.061 ^c^ | | 0.507 ^c^ | |  | | 0.448 | | 0.038 | | 0.089 |  |
| TG(18:2_35:2) | | Triglycerides | | HMDB0052413 | | -0.302 ^b^ | | -1.191 ^a^ | | 1.009 ^c^ | | 0.484 ^c^ | |  | | 0.866 | | 0.048 | | 0.631 |  |
| TG(18:2_36:0) | | Triglycerides | | HMDB0052455 | | -0.494 ^a^ | | -1.046 ^a^ | | 1.074 ^c^ | | 0.466 ^b^ | |  | | 0.652 | | 0.048 | | 0.397 |  |

**Table S8.** Serum metabolites that are differentially regulated in HMO-gavaged mice (HMO and BI+HMO groups) compared with those not receiving HMO (BI and control groups) (n = 10-14 mice/group). Values represent the normalized abundance levels and may contain negative values due to normalization (median and pareto scaling). All data processing and normalization were performed using MetaboAnalyst 6.0 software with default parameters unless otherwise described. Treatment groups: HMO (human milk oligosaccharides) group - orally gavaged with pooled 2′-fucosyllactose, lacto-N-tetraose and 3′-sialyllactose at 15 mg/day (5 mg/HMO) for 14 d; BI group - orally gavaged with *Bifidobacterium longum subsp. infantis* ATCC 15697 (1x10^9 CFU/d) on days 1, 4, and 9 of the 14 d experimental period; BI+HMO group - orally gavaged with HMO for all 14 d and BI on days 1, 4, and 9 of the 14 d experimental period; Control group – orally gavaged with PBS for 14 d. Adjusted P-values were calculated using two-way ANOVA with Tukey’s multiple comparison tests in R studio version 4.4.1. Mean values in the same row with different superscripts differ (P < 0.05). HMD - Human Metabolome Database.

| **Metabolites** | **Class** | **HMD IDs** | **HMO** | | **No HMO** | |  | **Adjusted P value** | | |
| --- | --- | --- | --- | --- | --- | --- | --- | --- | --- | --- |
|  |  |  | BI | No BI | BI | No BI |  | HMOs | BI | BI × HMO |
| **Up regulated** |  |  |  |  |  |  |  |  |  |  |
| CE(18:0) | Cholesterol Esters | HMDB10368 | -0.031 ^b^ | 0.409 ^b^ | 0.721 ^b^ | -1.099 ^a^ |  | 0.025 | <0.001 | 0.242 |
| CE(20:1) | Cholesterol Esters | HMDB05194 | -0.139 ^a^ | -0.054 ^a^ | 0.969 ^b^ | -0.776 ^a^ |  | 0.016 | 0.001 | 0.578 |
| DG(16:0_18:1) | Diglycerides | HMDB07101 | 0.497 ^b^ | 0.084 ^b^ | 0.361 ^b^ | -0.942 ^a^ |  | 0.018 | 0.121 | 0.128 |
| DG(18:1_18:1) | Diglycerides | HMDB07188 | -0.335 ^a^ | 0.448 ^b^ | 0.919 ^b^ | -1.032 ^a^ |  | 0.033 | <0.001 | 0.710 |
| DG(18:1_20:4) | Diglycerides | HMDB07199 | 0.530 ^b^ | -0.097 ^ab^ | 0.433 ^b^ | -0.867 ^a^ |  | 0.009 | 0.244 | 0.245 |
| TG(16:0_34:2) | Triglycerides | HMDB0005362 | -0.298 ^b^ | 0.607 ^c^ | 0.843 ^c^ | -1.152 ^a^ |  | 0.032 | <0.001 | 0.252 |
| TG(16:0_36:2) | Triglycerides | HMDB0005369 | -0.356 ^b^ | 0.564 ^c^ | 0.898 ^c^ | -1.106 ^a^ |  | 0.033 | <0.001 | 0.440 |
| TG(16:0_38:3) | Triglycerides | HMDB0005389 | -0.319 ^b^ | 0.475 ^c^ | 0.970 ^c^ | -1.126 ^a^ |  | 0.014 | <0.001 | 0.561 |
| TG(16:0_38:4) | Triglycerides | HMDB0005370 | -0.211 ^b^ | 0.455 ^bc^ | 0.841 ^c^ | -1.084 ^a^ |  | 0.028 | <0.001 | 0.422 |
| TG(17:0_36:3) | Triglycerides | HMDB0050323 | -0.327 ^b^ | 0.651 ^c^ | 0.848 ^c^ | -1.172 ^a^ |  | 0.031 | <0.001 | 0.213 |
| TG(17:0_36:4) | Triglycerides | HMDB0055545 | -0.439 ^a^ | 0.270 ^b^ | 1.191 ^c^ | -1.022 ^a^ |  | 0.004 | <0.001 | 0.490 |
| TG(18:0_34:2) | Triglycerides | HMDB0044682 | -0.207 ^b^ | 0.574 ^c^ | 0.940 ^c^ | -1.306 ^a^ |  | 0.003 | <0.001 | 0.111 |
| TG(18:0_36:3) | Triglycerides | HMDB0005405 | -0.338 ^b^ | 0.459 ^c^ | 1.081 ^d^ | -1.202 ^a^ |  | 0.003 | <0.001 | 0.586 |
| TG(18:0_36:4) | Triglycerides | HMDB0005411 | -0.527 ^b^ | 0.380 ^c^ | 1.211 ^d^ | -1.064 ^a^ |  | 0.003 | <0.001 | 0.490 |
| TG(18:0_36:5) | Triglycerides | HMDB0044687 | -0.200 ^b^ | 0.463 ^c^ | 0.970 ^c^ | -1.233 ^a^ |  | 0.004 | <0.001 | 0.289 |
| TG(18:1_34:1) | Triglycerides | HMDB0010437 | -0.336 ^b^ | 0.553 ^c^ | 0.905 ^c^ | -1.122 ^a^ |  | 0.026 | <0.001 | 0.422 |
| TG(18:1_36:2) | Triglycerides | HMDB0010447 | -0.354 ^b^ | 0.665 ^c^ | 0.867 ^c^ | -1.177 ^a^ |  | 0.029 | <0.001 | 0.218 |
| TG(18:1_36:4) | Triglycerides | HMDB0049150 | -0.397 ^a^ | 0.398 ^b^ | 1.004 ^b^ | -1.005 ^a^ |  | 0.025 | <0.001 | 0.998 |
| TG(18:1_36:6) | Triglycerides | HMDB0049402 | -0.038 ^b^ | 0.593 ^bc^ | 0.677 ^c^ | -1.231 ^a^ |  | 0.021 | <0.001 | 0.061 |
| TG(18:2_32:0) | Triglycerides | HMDB0052410 | -0.331 ^b^ | 0.546 ^c^ | 0.970 ^c^ | -1.185 ^a^ |  | 0.009 | <0.001 | 0.386 |
| TG(18:2_35:1) | Triglycerides | HMDB0050323 | -0.472 ^b^ | 0.507 ^c^ | 1.061 ^c^ | -1.096 ^a^ |  | 0.013 | <0.001 | 0.892 |
| TG(18:2_35:2) | Triglycerides | HMDB0052413 | -0.302 ^b^ | 0.484 ^c^ | 1.009 ^c^ | -1.191 ^a^ |  | 0.004 | <0.001 | 0.449 |
| TG(18:2_36:0) | Triglycerides | HMDB0052455 | -0.494 ^a^ | 0.466 ^b^ | 1.074 ^c^ | -1.046 ^a^ |  | 0.017 | <0.001 | 0.924 |
| TG(18:2_36:1) | Triglycerides | HMDB0052545 | -0.376 ^b^ | 0.485 ^c^ | 1.074 ^d^ | -1.183 ^a^ |  | 0.004 | <0.001 | 0.623 |
| TG(18:2_36:2) | Triglycerides | HMDB0052441 | -0.429 ^a^ | 0.497 ^b^ | 0.981 ^b^ | -1.048 ^a^ |  | 0.030 | <0.001 | 0.819 |
| TG(18:2_36:3) | Triglycerides | HMDB0052442 | -0.458 ^a^ | 0.350 ^b^ | 1.060 ^c^ | -0.952 ^a^ |  | 0.025 | <0.001 | 0.711 |
| TG(18:2_36:4) | Triglycerides | HMDB0052450 | -0.463 ^a^ | 0.209 ^b^ | 1.240 ^c^ | -0.986 ^a^ |  | 0.003 | <0.001 | 0.292 |
| TG(18:2_36:5) | Triglycerides | HMDB0052540 | -0.277 ^b^ | 0.262 ^b^ | 1.101 ^c^ | -1.086 ^a^ |  | 0.004 | <0.001 | 0.953 |
| TG(18:3_32:0) | Triglycerides | HMDB0052858 | -0.346 ^b^ | 0.525 ^c^ | 0.910 ^c^ | -1.089 ^a^ |  | 0.031 | <0.001 | 0.512 |
| TG(18:3_34:0) | Triglycerides | HMDB0052886 | -0.360 ^ab^ | 0.348 ^bc^ | 0.979 ^c^ | -0.967 ^a^ |  | 0.029 | <0.001 | 0.968 |
| TG(18:3_36:1) | Triglycerides | HMDB0052998 | -0.380 ^b^ | 0.452 ^c^ | 1.053 ^d^ | -1.125 ^a^ |  | 0.007 | <0.001 | 0.786 |
| TG(18:3_36:3) | Triglycerides | HMDB0052887 | -0.361 ^a^ | 0.380 ^b^ | 0.976 ^b^ | -0.995 ^a^ |  | 0.028 | <0.001 | 0.953 |
| TG(18:3_36:4) | Triglycerides | HMDB0052895 | -0.318 ^b^ | 0.347 ^c^ | 1.049 ^d^ | -1.078 ^a^ |  | 0.007 | <0.001 | 0.927 |
| TG(20:1_34:2) | Triglycerides | HMDB0050342 | -0.456 ^a^ | 0.303 ^b^ | 1.107 ^c^ | -0.954 ^a^ |  | 0.016 | <0.001 | 0.578 |
| TG(20:3_34:1) | Triglycerides | HMDB0051030 | -0.210 ^b^ | 0.512 ^c^ | 0.837 ^c^ | -1.140 ^a^ |  | 0.023 | <0.001 | 0.291 |
| **Down regulated** |  |  |  |  |  |  |  |  |  |  |
| 2-Hydroxy-2-methylbutyric acid | Organic acids | HMDB0001987 | 0.198 ^b^ | -0.678 ^a^ | -0.845 ^a^ | 1.325 ^c^ |  | 0.004 | <0.001 | 0.052 |
| 2-Hydroxybutyric acid | Organic acids | HMDB0000008 | 0.155 ^b^ | -0.399 ^ab^ | -0.979 ^a^ | 1.224 ^c^ |  | 0.003 | <0.001 | 0.340 |
| 3-Hydroxybutyric acid | Organic acids | HMDB0000011 | -0.112 ^a^ | -0.244 ^a^ | -0.672 ^a^ | 1.027 ^b^ |  | 0.020 | 0.001 | 0.307 |
| 3-Hydroxyisovaleric acid | Organic acids | HMDB0000754 | 0.183 ^b^ | -0.554 ^a^ | -0.825 ^a^ | 1.195 ^c^ |  | 0.017 | <0.001 | 0.192 |
| Allantoin | Alkaloids | HMDB0000462 | 0.283 ^b^ | -0.693 ^a^ | -0.813 ^a^ | 1.223 ^c^ |  | 0.025 | <0.001 | 0.112 |
| alpha-Aminobutyric acid | Amino acid - related | HMDB0000452 | 0.267 ^b^ | -0.173 ^b^ | -1.162 ^a^ | 1.067 ^c^ |  | 0.003 | <0.001 | 0.723 |
| alpha-Ketoisovaleric acid | Amino acid - related | HMDB0000019 | 0.079 ^b^ | -0.557 ^a^ | -0.860 ^a^ | 1.338 ^c^ |  | 0.003 | <0.001 | 0.056 |
| Butyric acid | Short chain fatty acid | HMDB0000039 | 0.150 ^b^ | -0.679 ^a^ | -0.724 ^a^ | 1.253 ^c^ |  | 0.023 | <0.001 | 0.057 |
| C12:1 | Acylcarnitines | HMDB0013326 | 0.177 ^b^ | -0.322 ^ab^ | -0.862 ^a^ | 1.007 ^c^ |  | 0.025 | <0.001 | 0.651 |
| C14 | Acylcarnitines | HMDB0005066 | 0.052 ^b^ | -0.079 ^b^ | -0.946 ^a^ | 0.973 ^c^ |  | 0.007 | <0.001 | 0.939 |
| C14:1 | Acylcarnitines | HMDB0002014 | -0.151 ^a^ | -0.530 ^a^ | -0.484 ^a^ | 1.165 ^b^ |  | 0.034 | <0.001 | 0.053 |
| C14:2 | Acylcarnitines | HMDB0013331 | 0.185 ^b^ | -0.628 ^a^ | -0.779 ^a^ | 1.222 ^c^ |  | 0.022 | <0.001 | 0.111 |
| C16 | Acylcarnitines | HMDB0000222 | 0.380 ^b^ | -0.585 ^a^ | -1.004 ^a^ | 1.209 ^c^ |  | 0.006 | <0.001 | 0.357 |
| C18 | Acylcarnitines | HMDB0000848 | 0.406 ^b^ | -0.528 ^a^ | -0.940 ^a^ | 1.062 ^c^ |  | 0.036 | <0.001 | 0.656 |
| C18:1 | Acylcarnitines | HMDB0006464 | 0.148 ^b^ | -0.476 ^a^ | -0.907 ^a^ | 1.235 ^c^ |  | 0.004 | <0.001 | 0.213 |
| C18:2 | Acylcarnitines | HMDB0006469 | -0.026 ^a^ | -0.466 ^a^ | -0.602 ^a^ | 1.094 ^b^ |  | 0.041 | <0.001 | 0.152 |
| C4OH | Acylcarnitines | HMDB0013127 | 0.114 ^b^ | -0.589 ^a^ | -0.839 ^a^ | 1.315 ^c^ |  | 0.004 | <0.001 | 0.061 |
| Choline | Vitamins & cofactors | HMDB0000097 | 0.056 ^b^ | -0.488 ^ab^ | -0.806 ^a^ | 1.237 ^c^ |  | 0.007 | <0.001 | 0.131 |
| Cytidine | Nucleoside | HMDB0000089 | 0.392 ^bc^ | -0.252 ^b^ | -1.093 ^a^ | 0.953 ^c^ |  | 0.014 | <0.001 | 0.622 |
| Deoxycytidine | Nucleoside | HMDB0000014 | 0.771 ^c^ | -0.399 ^b^ | -1.206 ^a^ | 0.834 ^c^ |  | 0.039 | <0.001 | 0.114 |
| Dimethylglycine | Amino Acids Derivatives | HMDB0000092 | 0.216 ^c^ | -0.406 ^b^ | -1.034 ^a^ | 1.224 ^d^ |  | 0.003 | <0.001 | 0.427 |
| Ethylmalonic acid | Organic acids | HMDB0000622 | -0.113 ^a^ | -0.415 ^a^ | -0.737 ^a^ | 1.265 ^b^ |  | 0.004 | <0.001 | 0.073 |
| gamma-Aminobutyric acid | Amino acid - related | HMDB0000112 | 0.226 ^c^ | -0.371 ^b^ | -1.075 ^a^ | 1.220 ^d^ |  | 0.003 | <0.001 | 0.536 |
| Glutamine | Amino acid | HMDB0000641 | 0.166 ^b^ | -0.620 ^a^ | -0.731 ^a^ | 1.185 ^c^ |  | 0.033 | <0.001 | 0.122 |
| Hex2Cer(d18:1/16:0) | Glycosylceramides | HMDB0006750 | 0.281 ^b^ | -0.616 ^a^ | -0.821 ^a^ | 1.156 ^c^ |  | 0.033 | <0.001 | 0.221 |
| HexCer(d18:1/16:0) | Glycosylceramides | HMDB0004971 | 0.305 ^b^ | -0.633 ^a^ | -0.864 ^a^ | 1.193 ^c^ |  | 0.022 | <0.001 | 0.203 |
| HexCer(d18:1/18:0) | Glycosylceramides | HMDB0004972 | 0.209 ^b^ | -0.516 ^a^ | -0.849 ^a^ | 1.157 ^c^ |  | 0.019 | <0.001 | 0.272 |
| HexCer(d18:1/23:0) | Glycosylceramides | HMDB0341524 | 0.236 ^b^ | -0.665 ^a^ | -0.756 ^a^ | 1.185 ^c^ |  | 0.039 | <0.001 | 0.128 |
| HexCer(d18:1/24:0) | Glycosylceramides | HMDB0004978 | 0.322 ^b^ | -0.699 ^a^ | -0.797 ^a^ | 1.175 ^c^ |  | 0.047 | <0.001 | 0.159 |
| HexCer(d18:1/24:1) | Glycosylceramides | HMDB0004975 | 0.310 ^b^ | -0.659 ^a^ | -0.809 ^a^ | 1.158 ^c^ |  | 0.043 | <0.001 | 0.202 |
| HexCer(d18:2/24:0) | Glycosylceramides | HMDB0341530 | 0.034 ^b^ | -0.493 ^ab^ | -0.824 ^a^ | 1.283 ^c^ |  | 0.004 | <0.001 | 0.091 |
| Hippuric acid | Organic acids | HMDB0000714 | -0.023 ^a^ | -0.628 ^a^ | -0.541 ^a^ | 1.193 ^b^ |  | 0.045 | <0.001 | 0.052 |
| Histidine | Amino acid | HMDB0000177 | 0.225 ^b^ | -0.575 ^a^ | -0.808 ^a^ | 1.159 ^c^ |  | 0.028 | <0.001 | 0.217 |
| Indole | Indoles & Derivatives | HMDB0000738 | 0.292 ^b^ | -0.646 ^a^ | -0.804 ^a^ | 1.158 ^c^ |  | 0.041 | <0.001 | 0.202 |
| Indoxyl sulfate | Indoles & Derivatives | HMDB0000682 | -0.307 ^a^ | -0.424 ^a^ | -0.347 ^a^ | 1.077 ^b^ |  | 0.043 | 0.005 | 0.053 |
| Isoleucine | Amino acid | HMDB0000172 | 0.242 ^b^ | -0.478 ^a^ | -0.854 ^a^ | 1.089 ^c^ |  | 0.029 | <0.001 | 0.427 |
| LysoPC a C14:0 | Glycerophospholipids | HMDB0010379 | 0.386 ^b^ | -0.669 ^a^ | -0.856 ^a^ | 1.139 ^c^ |  | 0.048 | <0.001 | 0.269 |
| LysoPC a C16:1 | Glycerophospholipids | HMDB0010383 | 0.403 ^b^ | -0.619 ^a^ | -0.925 ^a^ | 1.140 ^c^ |  | 0.027 | <0.001 | 0.390 |
| LysoPC a C18:1 | Glycerophospholipids | HMDB0002815 | 0.395 ^b^ | -0.631 ^a^ | -0.912 ^a^ | 1.148 ^c^ |  | 0.028 | <0.001 | 0.348 |
| LysoPC a C20:4 | Glycerophospholipids | HMDB0010395 | 0.389 ^c^ | -0.517 ^b^ | -1.079 ^a^ | 1.207 ^d^ |  | 0.003 | <0.001 | 0.548 |
| Malonic acid | Organic acids | HMDB0000691 | -0.218 ^a^ | -0.424 ^a^ | -0.614 ^a^ | 1.256 ^b^ |  | 0.006 | <0.001 | 0.052 |
| Methylmalonic acid | Organic acids | HMDB0000202 | -0.045 ^a^ | -0.558 ^a^ | -0.615 ^a^ | 1.218 ^b^ |  | 0.022 | <0.001 | 0.057 |
| N-Acetyl-Glycine | Amino Acids Derivatives | HMDB0000532 | 0.044 ^b^ | -0.468 ^ab^ | -0.844 ^a^ | 1.268 ^c^ |  | 0.004 | <0.001 | 0.124 |
| N-Acetyl-Isoleucine | Amino Acids Derivatives | HMDB0061684 | 0.153 ^b^ | -0.436 ^ab^ | -0.758 ^a^ | 1.042 ^c^ |  | 0.043 | <0.001 | 0.388 |
| Nudifloramide | Pyridines | HMDB0004193 | 0.106 ^b^ | -0.516 ^ab^ | -0.818 ^a^ | 1.228 ^c^ |  | 0.009 | <0.001 | 0.148 |
| Ornithine | Amino acid | HMDB0000214 | 0.374 ^bc^ | -0.301 ^b^ | -1.020 ^a^ | 0.947 ^c^ |  | 0.024 | <0.001 | 0.819 |
| PC aa C30:0 | Glycerophospholipids | HMDB0007869 | 0.211 ^b^ | -0.671 ^a^ | -0.778 ^a^ | 1.238 ^c^ |  | 0.022 | <0.001 | 0.083 |
| PC aa C32:1 | Glycerophospholipids | HMDB0007872 | 0.253 ^b^ | -0.590 ^a^ | -0.854 ^a^ | 1.192 ^c^ |  | 0.019 | <0.001 | 0.208 |
| PC aa C32:2 | Glycerophospholipids | HMDB0007874 | 0.340 ^b^ | -0.699 ^a^ | -0.921 ^a^ | 1.279 ^c^ |  | 0.006 | <0.001 | 0.091 |
| PC aa C32:3 | Glycerophospholipids | HMDB0007875 | 0.200 ^b^ | -0.678 ^a^ | -0.828 ^a^ | 1.306 ^c^ |  | 0.007 | <0.001 | 0.053 |
| PC aa C34:3 | Glycerophospholipids | HMDB0007881 | 0.338 ^b^ | -0.560 ^a^ | -0.889 ^a^ | 1.110 ^c^ |  | 0.033 | <0.001 | 0.422 |
| PC aa C34:4 | Glycerophospholipids | HMDB0007883 | 0.315 ^b^ | -0.430 ^a^ | -0.965 ^a^ | 1.079 ^c^ |  | 0.018 | <0.001 | 0.689 |
| PC aa C36:0 | Glycerophospholipids | HMDB0007886 | 0.273 ^b^ | -0.698 ^a^ | -0.809 ^a^ | 1.234 ^c^ |  | 0.023 | <0.001 | 0.094 |
| PC aa C36:1 | Glycerophospholipids | HMDB0007887 | 0.249 ^b^ | -0.742 ^a^ | -0.748 ^a^ | 1.241 ^c^ |  | 0.032 | <0.001 | 0.061 |
| PC aa C36:3 | Glycerophospholipids | HMDB0007921 | 0.193 ^b^ | -0.674 ^a^ | -0.749 ^a^ | 1.230 ^c^ |  | 0.026 | <0.001 | 0.077 |
| PC aa C36:4 | Glycerophospholipids | HMDB0007889 | 0.268 ^b^ | -0.610 ^a^ | -0.815 ^a^ | 1.156 ^c^ |  | 0.033 | <0.001 | 0.218 |
| PC aa C36:5 | Glycerophospholipids | HMDB0007890 | 0.158 ^b^ | -0.594 ^a^ | -0.768 ^a^ | 1.204 ^c^ |  | 0.023 | <0.001 | 0.128 |
| PC aa C36:6 | Glycerophospholipids | HMDB0008690 | 0.318 ^b^ | -0.581 ^a^ | -0.967 ^a^ | 1.230 ^c^ |  | 0.006 | <0.001 | 0.252 |
| PC aa C38:0 | Glycerophospholipids | HMDB0007893 | 0.246 ^b^ | -0.707 ^a^ | -0.825 ^a^ | 1.286 ^c^ |  | 0.011 | <0.001 | 0.057 |
| PC aa C38:1 | Glycerophospholipids | HMDB0007894 | 0.263 ^b^ | -0.734 ^a^ | -0.822 ^a^ | 1.293 ^c^ |  | 0.011 | <0.001 | 0.052 |
| PC aa C38:3 | Glycerophospholipids | HMDB0008020 | 0.282 ^b^ | -0.706 ^a^ | -0.862 ^a^ | 1.286 ^c^ |  | 0.008 | <0.001 | 0.061 |
| PC aa C38:4 | Glycerophospholipids | HMDB0007988 | 0.279 ^b^ | -0.627 ^a^ | -0.940 ^a^ | 1.288 ^c^ |  | 0.004 | <0.001 | 0.118 |
| PC aa C38:5 | Glycerophospholipids | HMDB0007989 | 0.224 ^b^ | -0.579 ^a^ | -0.914 ^a^ | 1.269 ^c^ |  | 0.004 | <0.001 | 0.146 |
| PC aa C38:6 | Glycerophospholipids | HMDB0007991 | 0.380 ^b^ | -0.663 ^a^ | -0.935 ^a^ | 1.218 ^c^ |  | 0.012 | <0.001 | 0.211 |
| PC aa C40:1 | Glycerophospholipids | HMDB0007993 | 0.118 ^b^ | -0.673 ^a^ | -0.707 ^a^ | 1.261 ^c^ |  | 0.021 | <0.001 | 0.053 |
| PC aa C40:3 | Glycerophospholipids | HMDB0008086 | 0.193 ^b^ | -0.571 ^a^ | -0.799 ^a^ | 1.178 ^c^ |  | 0.023 | <0.001 | 0.192 |
| PC aa C40:4 | Glycerophospholipids | HMDB0008054 | 0.297 ^b^ | -0.594 ^a^ | -0.889 ^a^ | 1.186 ^c^ |  | 0.018 | <0.001 | 0.246 |
| PC aa C40:5 | Glycerophospholipids | HMDB0008055 | 0.304 ^b^ | -0.631 ^a^ | -0.953 ^a^ | 1.280 ^c^ |  | 0.004 | <0.001 | 0.134 |
| PC aa C40:6 | Glycerophospholipids | HMDB0008057 | 0.336 ^b^ | -0.706 ^a^ | -0.918 ^a^ | 1.288 ^c^ |  | 0.005 | <0.001 | 0.077 |
| PC aa C42:1 | Glycerophospholipids | HMDB0008059 | 0.102 ^b^ | -0.629 ^a^ | -0.731 ^a^ | 1.258 ^c^ |  | 0.016 | <0.001 | 0.061 |
| PC aa C42:4 | Glycerophospholipids | HMDB0008191 | 0.188 ^b^ | -0.665 ^a^ | -0.706 ^a^ | 1.184 ^c^ |  | 0.046 | <0.001 | 0.103 |
| PC aa C42:5 | Glycerophospholipids | HMDB0008257 | 0.275 ^b^ | -0.596 ^a^ | -0.895 ^a^ | 1.216 ^c^ |  | 0.012 | <0.001 | 0.203 |
| PC ae C30:0 | Glycerophospholipids | HMDB0013341 | 0.180 ^b^ | -0.694 ^a^ | -0.722 ^a^ | 1.235 ^c^ |  | 0.029 | <0.001 | 0.061 |
| PC ae C32:1 | Glycerophospholipids | HMDB0013404 | 0.286 ^b^ | -0.725 ^a^ | -0.840 ^a^ | 1.279 ^c^ |  | 0.012 | <0.001 | 0.061 |
| PC ae C34:0 | Glycerophospholipids | HMDB0013405 | 0.201 ^b^ | -0.728 ^a^ | -0.740 ^a^ | 1.267 ^c^ |  | 0.023 | <0.001 | 0.052 |
| PC ae C34:1 | Glycerophospholipids | HMDB0013412 | 0.250 ^b^ | -0.720 ^a^ | -0.774 ^a^ | 1.244 ^c^ |  | 0.025 | <0.001 | 0.063 |
| PC ae C34:2 | Glycerophospholipids | HMDB0011151 | 0.203 ^b^ | -0.723 ^a^ | -0.692 ^a^ | 1.212 ^c^ |  | 0.048 | <0.001 | 0.061 |
| PC ae C36:3 | Glycerophospholipids | HMDB0013425 | 0.191 ^b^ | -0.680 ^a^ | -0.772 ^a^ | 1.261 ^c^ |  | 0.018 | <0.001 | 0.061 |
| PC ae C36:4 | Glycerophospholipids | HMDB0013407 | 0.257 ^b^ | -0.702 ^a^ | -0.830 ^a^ | 1.275 ^c^ |  | 0.012 | <0.001 | 0.061 |
| PC ae C38:0 | Glycerophospholipids | HMDB0013408 | 0.275 ^b^ | -0.591 ^a^ | -0.926 ^a^ | 1.241 ^c^ |  | 0.007 | <0.001 | 0.196 |
| PC ae C38:4 | Glycerophospholipids | HMDB0013420 | 0.197 ^b^ | -0.674 ^a^ | -0.824 ^a^ | 1.301 ^c^ |  | 0.007 | <0.001 | 0.055 |
| PC ae C38:5 | Glycerophospholipids | HMDB0013432 | 0.241 ^b^ | -0.678 ^a^ | -0.861 ^a^ | 1.298 ^c^ |  | 0.006 | <0.001 | 0.061 |
| PC ae C38:6 | Glycerophospholipids | HMDB0013409 | 0.273 ^b^ | -0.682 ^a^ | -0.870 ^a^ | 1.278 ^c^ |  | 0.008 | <0.001 | 0.078 |
| PC ae C40:1 | Glycerophospholipids | HMDB0013433 | 0.292 ^b^ | -0.664 ^a^ | -0.865 ^a^ | 1.237 ^c^ |  | 0.015 | <0.001 | 0.130 |
| PC ae C40:6 | Glycerophospholipids | HMDB0013422 | 0.251 ^b^ | -0.716 ^a^ | -0.843 ^a^ | 1.308 ^c^ |  | 0.007 | <0.001 | 0.052 |
| PC ae C42:0 | Glycerophospholipids | HMDB0013423 | 0.198 ^b^ | -0.563 ^a^ | -0.823 ^a^ | 1.188 ^c^ |  | 0.019 | <0.001 | 0.196 |
| PC ae C42:1 | Glycerophospholipids | HMDB0013434 | 0.169 ^b^ | -0.603 ^a^ | -0.860 ^a^ | 1.293 ^c^ |  | 0.005 | <0.001 | 0.078 |
| PC ae C42:2 | Glycerophospholipids | HMDB0013438 | 0.248 ^b^ | -0.641 ^a^ | -0.842 ^a^ | 1.235 ^c^ |  | 0.015 | <0.001 | 0.128 |
| PC ae C42:3 | Glycerophospholipids | HMDB0013458 | 0.359 ^b^ | -0.678 ^a^ | -0.849 ^a^ | 1.169 ^c^ |  | 0.036 | <0.001 | 0.211 |
| PC ae C44:4 | Glycerophospholipids | HMDB0013453 | 0.179 ^b^ | -0.695 ^a^ | -0.712 ^a^ | 1.228 ^c^ |  | 0.033 | <0.001 | 0.061 |
| p-Cresol sulfate | Organic acids | HMDB0011635 | 0.149 ^b^ | -0.720 ^a^ | -0.666 ^a^ | 1.236 ^c^ |  | 0.038 | <0.001 | 0.052 |
| Phenylalanine | Amino acid | HMDB0000159 | 0.319 ^b^ | -0.632 ^a^ | -0.870 ^a^ | 1.183 ^c^ |  | 0.023 | <0.001 | 0.221 |
| Putrescine | Biogenic Amines | HMDB0001414 | 0.184 ^b^ | -0.436 ^ab^ | -0.863 ^a^ | 1.115 ^c^ |  | 0.019 | <0.001 | 0.394 |
| SM C16:0 | Sphingomyelins | HMDB0010169 | 0.266 ^b^ | -0.734 ^a^ | -0.816 ^a^ | 1.284 ^c^ |  | 0.014 | <0.001 | 0.054 |
| SM C18:0 | Sphingomyelins | HMDB0001348 | 0.273 ^b^ | -0.671 ^a^ | -0.845 ^a^ | 1.242 ^c^ |  | 0.016 | <0.001 | 0.112 |
| SM C18:1 | Sphingomyelins | HMDB0012100 | 0.247 ^b^ | -0.703 ^a^ | -0.840 ^a^ | 1.296 ^c^ |  | 0.008 | <0.001 | 0.056 |
| SM C20:2 | Sphingomyelins | HMDB0013465 | 0.189 ^b^ | -0.486 ^a^ | -0.979 ^a^ | 1.277 ^c^ |  | 0.003 | <0.001 | 0.208 |
| SM C24:0 | Sphingomyelins | HMDB0011697 | 0.260 ^b^ | -0.704 ^a^ | -0.874 ^a^ | 1.318 ^c^ |  | 0.004 | <0.001 | 0.052 |
| SM C24:1 | Sphingomyelins | HMDB0012107 | 0.284 ^b^ | -0.709 ^a^ | -0.901 ^a^ | 1.327 ^c^ |  | 0.004 | <0.001 | 0.052 |
| SM C26:1 | Sphingomyelins | HMDB0013461 | 0.172 ^b^ | -0.622 ^a^ | -0.766 ^a^ | 1.216 ^c^ |  | 0.023 | <0.001 | 0.111 |
| SM(OH) C16:1 | Sphingomyelins | HMDB0013463 | 0.252 ^b^ | -0.721 ^a^ | -0.829 ^a^ | 1.298 ^c^ |  | 0.009 | <0.001 | 0.052 |
| SM(OH) C22:1 | Sphingomyelins | HMDB0013466 | 0.071 ^b^ | -0.663 ^a^ | -0.672 ^a^ | 1.264 ^c^ |  | 0.022 | <0.001 | 0.051 |
| SM(OH) C24:1 | Sphingomyelins | HMDB0013469 | 0.211 ^b^ | -0.712 ^a^ | -0.773 ^a^ | 1.274 ^c^ |  | 0.018 | <0.001 | 0.054 |
| Valine | Amino acid | HMDB0000883 | 0.351 ^b^ | -0.592 ^a^ | -0.909 ^a^ | 1.150 ^c^ |  | 0.023 | <0.001 | 0.352 |
